# Design and Synthesis of Scopoletin Sulfonate Derivatives as Potential Insecticidal Agents

**DOI:** 10.3390/molecules28020530

**Published:** 2023-01-05

**Authors:** Congmin Liu, Panyuan Zheng, Hongmei Wang, Yan Wei, Chuanping Wang, Shuanghong Hao

**Affiliations:** College of Chemistry and Pharmaceutical Sciences, Research Center of Bio-Pesticides Engineering & Tech. of Shandong Province, Qingdao Agricultural University, Qingdao 266109, China

**Keywords:** scopoletin, coumarin, acaricidal, insecticidal, AChE

## Abstract

(1) Background: Scopoletin and scoparone, two naturally occurring coumarins, have garnered considerable attention and have been introduced to the market in China due to their high insecticidal efficacy and low toxicity. To investigate the structure–activity relationship of these coumarins, a series of scopoletin derivatives with aryl sulfate at C7 and different substitutes at C3 were designed and synthesized, and their insecticidal activity was studied. (2) Methods: A total of 28 new scopoletin derivatives were designed and synthesized. Most target compounds exhibited moderate insecticidal activity against the phytophagous mite *Tetranychus cinnabarinus* and the brine shrimp *Artemia salina*. (3) Results: Among these compounds, compounds **5a** and **5j** possessed the best insecticidal activities against *T. cinnabarinus,* with LC_50_ values of 57.0 and 20.0 μg/mL, respectively, whereas that of the control drug was 15.0 μg/mL. Compound **4j** exhibited selective insecticidal activities against *A. salina*, with an LC_50_ value of 9.36 μg/mL, whereas its LC_50_ value against *T. cinnabarinus* was 93.0 μg/mL. The enzymatic inhibitory activity on acetylcholinesterase (AChE) showed a consistent tendency with the insecticidal activity. Further molecular docking analyses predicted the binding conformations of these compounds, which showed a good correlation between the insecticidal activity and the binding scores. (4) Conclusions: In general, a decreased electron cloud density of the Δ^3,4^ olefinic bond is beneficial for improving the insecticidal activity against both *T. cinnabarinus* and *A. salina*. In addition, naphthyl or benzene groups with a sulfate ester at the C7 position could further improve the insecticidal activity against *A. salina*. AChE was implied to be a site of action for potential insecticidal activity. The results provide insight into the rational design of a new generation of effective coumarin insecticides.

## 1. Introduction

Phytophagous mites are distributed in almost all crop production areas on earth. The most seriously disruptive of these mites are Tetranychidae and Eriophyidae, which consist of *T. cinnabarinus*, *Tetranychus urticae*, *Panonychus citri* and *Panonychus ulmi* [1,2]. These mite species destroy vegetables, fruits, crops, cotton, etc., by sucking the fluid from these crops [3,4,5]. Most plant pest mites are parthenogenetic, fertile, adaptive, mutagenic and resistant, and they are considered among the most difficult pests to prevent [6,7]. To date, using acaricides remains the most effective mode to control plant mites [8,9]. However, the frequent and improper application of limited species of acaricides inevitably causes mite resistance to acaricides [10,11,12,13]. Therefore, few optional chemicals are available to effectively control pest mites and the development of new acaricides with novel targets to prevent mite resistance. To this end, naturally occurring compounds could be used to fight the adverse effects of disease pathogens, insects, weeds, etc.

**Figure 1:**
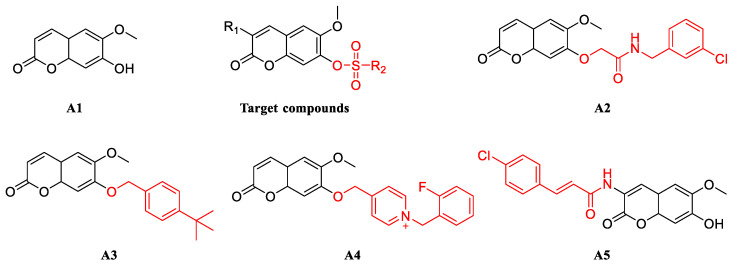
Chemical structures of scopoletin (**A1**) and its synthetic analogues (**A2**, **A3**, **A4**, **A5**).

As a typical natural insecticide, scopoletin (Figure 1(A1)) can be synthesized and accumulated in many plants when they are infested by pathogens or insects [14,15,16], and this compound has been registered as a botanical acaricide in China. Scopoletin has been demonstrated to have notable contact acaricidal and repellant activity, and it has been shown to inhibit egg laying, which could also decrease the reproductive rate and restrain population development [17,18]. Further research shows that scopoletin could suppress the activity of mite acetylcholinesterase (AChE), Ca^2+^-ATPase, etc. [19,20,21]. In recent years (from 2015 to the present), a series of excellent works on the structure–activity relationship of scopoletin have been published to further study more active insecticides. For example, Luo et al. (2018) reported that the etherification of 7-hydroxy of scopoletin could improve acaricidal activity (Figure 1(A2,A3)) [22] N. Khunnawutmanotham et al. (2016) showed that the introduction of a benzylpyridyl-4-methylene moiety to the hydroxy group at C7 of scopoletin could greatly improve the inhibition of AchE (Figure 1(A4)) [23]. Furthermore, Li et al. (2015) found that cinnamic amide at C3 of scopoletin could modify the antitumor activity (Figure 1(A5)) [24].

**Scheme 1:**
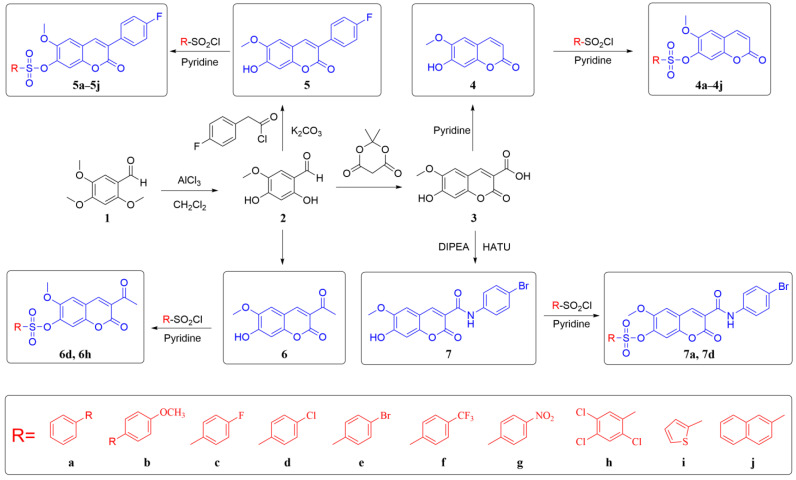
Synthesis of scopoletin aryl sulfate derivatives. Compounds **4a**−**4j** and **5a**−**5j** were synthesized from **4** and **5**, respectively. **6d** and **6h** were derived from **6**, while **7a** and **7d** were from **7**.

To identify potential green insecticidal agents and investigate the structure–activity relationship of these coumarins, a total of 28 scopoletin derivatives with aryl sulfate at C7 and different substitutes at C3 were designed and synthesized in this study (Scheme 1). The acaricidal activities of the target compounds against *A. salina* and *T. cinnabarinus* were screened, and the structure–activity relationship was summarized. The ability of these compounds to inhibit AChE was examined, and molecular docking was explored. The results provide information for designing compounds with higher acaricidal activity.

## 2. Results and Discussion

### 2.1. Insecticidal Activities against T. cinnabarinus

**Figure 2:**
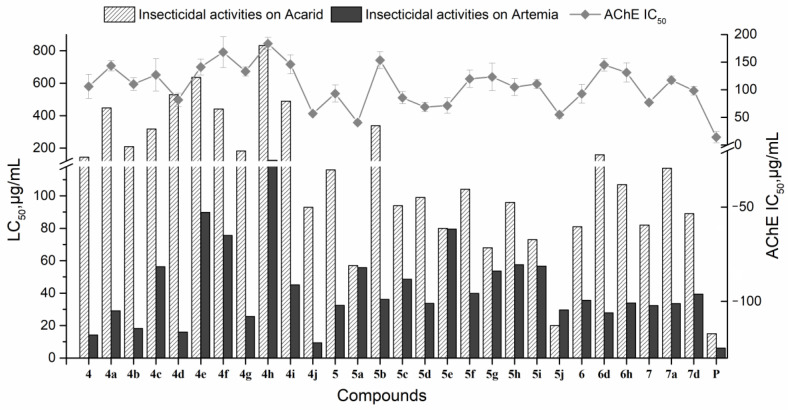
Insecticidal activities against Acarid (LC_50_, μg/mL, 72 h) and Artemia (LC_50_, μg/mL, 12 h) and the AChE inhibition (IC_50_, μg/mL) of synthetic compounds. Cyetpyrafen was used as a positive control.

The acaricidal activities (LC_50_, μg/mL) of the title compounds against the pest mite *T. cinnabarinus* are shown in Figure 2 (Supporting Information, Table S1). For scopoletin (**4**) and its sulfonate derivatives (**4a**–**4j**), all sulfonate-substituted compounds showed less potential than the original compound scopoletin, except for the naphthalenesulfonate of scopoletin (**4j**), whose LC_50_ is much lower than that of compound **4**. In the substituted benzene sulfonate derivatives **4a**–**4h**, the electro-donating substituent (4-OCH_3_) on the benzene ring appeared to be more favorable than the electro-withdrawing substituents (4-F, Cl, Br or CF_3_), but 4-NO_2_ was the exception to this trend. The LC_50_ of compound **4b** was less than those of compound **4a**, **4c**–**4f** and **4h** but greater than that of **4g,** which proves that substitution 4-NO_2_ increased toxicity. The higher acaricidal toxicity of 4-NO_2_ benzene sulfonate (**4g**) may be attributed to its lower lipophicity and higher topological polar surface area.

For 3-(4-F-phenyl)-scopoletin (**5**) and its sulfonate derivatives (compounds **5a**–**5j**), the acaricidal activities of most the sulfonate-substituted compounds (except compound **5b**) were improved. In particular, naphthalene-2-sulfonate substituted compound **5j** exhibited a toxicity that was nearly 5 times higher than that of compound **5**. Benzene sulfonate-substituted compound **5a**, 4-NO_2_-benzene sulfonate-substituted compound **5g**, and thienyl-2-sulfonate-substituted compound **5i** are approximately 2 times more toxic than compound **5**. Interestingly, electro-donating substituent (4-OCH_3_) on the benzene ring does not favor acaricidal activity compared to the electro-withdrawing substituents (4-F, Cl, Br, CF_3_ and NO_2_).

The 2 sulfonate-substituted compounds (compounds **6d**, **6h**, **7a**, and **7d**) of 3-acetyl scopoletin (**6**) and 3-(4-Br-aniline formyl) scopoletin (**7**) were less toxic than their original compounds, indicating that sulfonate substitution does not help acaricidal activity.

Overall, 3-(4-F-phenyl) and 7-aryl sulfonate substitutions on scopoletin improved the acaricidal activity of scopoletin. Naphthalene-2-sulfonate seems to be the best substitute for the acaricidal activity of scopoletin, which may be attributed to its large volume.

### 2.2. Insecticidal Activities against Artemia

The toxicity (LC_50_ values of 6 h and 12 h) of the title compounds against Artemia are shown in Figure 2 (Supporting Information, Table S2). For scopoletin (**4**) and its sulfonate derivatives (**4a**–**4j**), only the naphthalenesulfonate of scopoletin (**4j**) is more toxic than the unsubstituted compound scopoletin (**4**). The 4-OCH_3_-benzene sulfonate of scopoletin (**4b**) and the 4-Cl-benzene sulfonate of scopoletin (**4d**) are slightly less toxic to Artemia than the unsubstituted compound scopoletin (**4**) at 12 h. For 3-(4-F-phenyl)-scopoletin (**5**) and its sulfonate derivatives (**5a–5j**), only the naphthalenesulfonate-substituted compound (**5j**) was slightly more toxic to Artemia at 12 h than the original compound 3-(4-F-phenyl)-scopoletin (**5**). The 4-Cl-benzene sulfonate-substituted compound (**5d**) is nearly as toxic to Artemia as the original compound (**5**) at 12 h. For 3-acetyl-scopoletin (**6**), benzene sulfonate substitution may improve the toxicity (the LC_50_ of **6d** and **6h** at 6 h is less than that of the original compound (**6**). For 3-(*p*-Br-aniline formyl) scopoletin (**7**), benzene sulfonate substitution decreased the toxicity to Artemia (the LC_50_ values of **7a** and **7d** at 6 h and 12 h were higher than that of the original compound (**7**). Overall, naphthalenesulfonate substitution at the 7-site of scopoletin may not only improve the acaricidal activity but also increase the toxicity to Artemia compared to the lead compound Scopoletin.

### 2.3. AChE Inhibition

The in vitro AChE inhibition of the title compounds in *T. cinnabarinus* is shown in Figure 2 (Supporting Information, Table S1). The results show that the AChE inhibitory rates of the title compounds positively correlated with their acaricidal activities. Among all the tested samples, compounds **4j**, **5a**, and **5j** showed the most potent activities against AChE, with IC_50_ values of 56.75 ± 2.10, 40.60 ± 4.16, and 54.83 ± 6.39 μg/mL, respectively, while those of other compounds exhibited only moderate inhibition (Figure 2). For scopoletin (**4**) and its sulfonate derivatives (**4a**–**4j**), the 4-Cl benzene sulfonate of scopoletin (**4d**) and the naphthalenesulfonate of scopoletin (**4j**) exhibited the highest inhibition of AchE, which was even greater than that of the original compound scopoletin (**4**). However, only compound **4j** showed higher acaricidal activities than scopoletin. Moreover, compound **4b**, **4c** and **4g** also showed higher inhibition of AchE than other sulfonate derivatives of scopoletin, which is in consonance with their acaricidal activities.

For 3-(4-fluorophenyl)-scopoletin (**5**) and its sulfonate derivatives (**5a**–**5j**), compound **5c**, **5d, 5e, 5g, 5i**, and **5j** all exhibited improved AChE inhibition compared with their original compound **5**. Accordingly, these compounds also have higher acaricidal activities than the original compound 3-(4-fluorophenyl)-scopoletin (**5**), with the exception of compound **5a.** Although its acaricidal activity is much higher than that of **5,** its ability to inhibit AChE did not markedly differ from that of compound **5**. Conversely, compound **5f** and **5h** less potently inhibited AChE that compound **5** despite their higher acaricidal activities, which suggests that AChE may not be the only action site of these compounds. The 4 sulfonate-substituted compounds (**6d**, **6 h**, **7a**, and **7d**) of 3-acetyl-scopoletin (**6**) and 3-(4-bromoaniline formyl) scopoletin (**7**) showed lower AChE inhibition than their respective original compounds **6** and **7**, which is consistent with their acaricidal activities.

### 2.4. Molecular Docking

**Figure 3:**
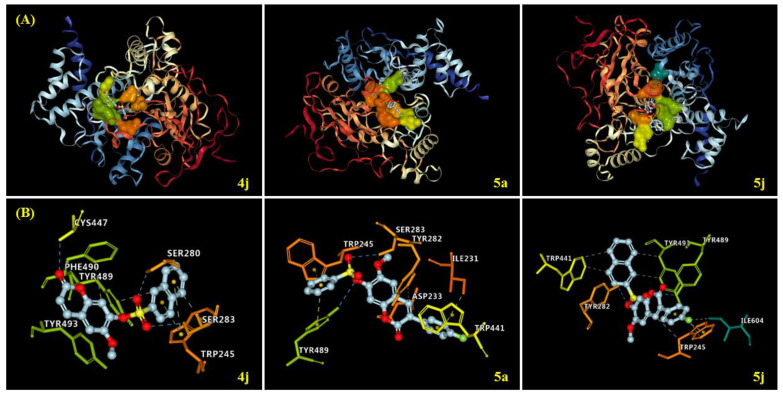
Molecular docking of compounds **4j**, **5a**, and **5j** at the binding site of acetylcholinesterase (**A**) Docking poses of **4j**, **5a**, and **5j**. (**B**) Interactions of **4j**, **5a**, and **5j**. The π–π stacking, hydrogen bonds, and hydrophobic interactions are represented by the green, blue, and gray lines, respectively.

Molecular docking between the typical compounds and target protein (6ary) was studied to elucidate their mutual effects and structure–activity relationship. The results of molecular docking are shown in Figure 3, which presents appropriate interactions with the main amino acid residues at the active site of the target protein. The docking pose analysis suggested that compounds **4j**, **5a**, and **5j** could easily fit in the catalytic pocket of AChE, showing grid scores of −64.66 (**4j**), −65.41 (**5a**) and −72.95 (**5j**), respectively. Compound **4j** formed π–π stacking with the TRP245 residue, hydrogen bonds with the CYS447, SER283, TYR489, and SER280 residues, and hydrophobic interactions with the TYR493, TYR489, and PHE490 residues. Compound **5a** mainly interacted with TRP245, TYR489, and TRP441 residues via π–π stacking, with SER283, TYR282 and TYR489 residues via hydrogen bonds and with TYR282, ILE231, and ASP233 residues via hydrophobic interactions. Similarly, compound **5j** formed π–π stacking with the TRP245 residue, hydrogen bonds with the TYR282 and TYR489 residues, and hydrophobic interactions with the ILE604, TRP245, TYR493, and TRP441 residues. This docking simulation revealed the importance of the lactone ring in the structures of compounds **4j**, **5a**, and **5j**, which could both form hydrogen bonds with TYR489. The molecular docking results are consistent with the biodetection data in vitro. Modifying the skeleton compound with a naphthalene nucleus may potently enhance the ability of these compounds to inhibit AChE.

## 3. Materials and Methods

### 3.1. General Remarks

All regular solvents and reagents were purchased from Qingdao Chemical Reagents Marketing Companies without further treatment. 2,4,5-Trimethoxybenzaldehyde, 2,2-dimethyl-1,3-dioxane-4,6-dione, 4-fluorophenylacetyl chloride, HATU (2-(7-azabenzotriazol-1-yl)-N,N,N’,N’-tetramethyluronium hexafluorophosphate) and DIPEA (N,N-diisopropylethylamine) were supplied by Saen Chemical Technology Co., Ltd.(Shanghai, China), Ethyl acetoacetate (Tianjin Damao Chemical Reagent Factory, Tianjin, China) and all sulfonyl chlorides used (Shanghai Macklin Biochemical Co., Ltd., Shanghai, China) were obtained separately from the different commercial sources listed above. Reactions were monitored by thin-layer chromatography (TLC) on silica gel GF_254_ and visualized under UV light (254 nm or 365 nm). The melting points (uncorrected) of compounds were measured using an X-4B micro melting point instrument (Yidian Physical Optical Instrument Co., Ltd. Shanghai, China). The ^1^H and ^13^C NMR spectra were obtained with a BRUKER AVANCE III HD 500 MHz (Bruker-Biospin, Billerica, MA, USA) using dimethyl sulfoxide (DMSO-*d*_6_) or chloroform (CDCl_3_) as the solvent. High-resolution mass spectra (HRESI TOF (+)) were recorded on a SolariX70FT-ICR-MS (Bruker-Biospin, Billerica, MA, USA). X-ray diffraction was measured with a Bruker D8 QUEST-X (Bruker-Biospin, Billerica, MA, USA).

### 3.2. Procedures for the Synthesis of Compounds in Scheme 1

#### 3.2.1. Preparation of 2,4-dihydroxy-5-methoxybenzaldehyde (**2**) [23]

Aluminum (III) chloride (5.4 g, 40 mmol) was added in portions over 30 min to a solution of 2,4,5-trimethoxybenzaldehyde (2.0 g, 10 mmol) in dichloromethane (120 mL). After stirring at room temperature for 24 h, the reaction mixture was poured into ice water (200 mL); concentrated hydrochloric acid was added to adjust the pH to 1. The dichloromethane layer was separated and washed with water. The organic layer was dried with anhydrous sodium sulfate, filtered, and concentrated to produce 2,4-dihydroxy-5-methoxybenzaldehyde. A white acicular crystal with the following characteristics was obtained: yield 86.3%, m.p. 203–205 °C, ^1^H NMR (500 MHz, CDCl_3_) δ: 11.25 (s, 1H), 9.60 (s, 1H), 6.94 (s, 1H), 6.82 (s, 1H), 6.45 (s, 1H), and 3.84 (s, 3H).

#### 3.2.2. Preparation of 7-hydroxy-6-methoxy-2-oxo-2H-chrom-ene-3-carboxylic Acid (**3**) [25]

A mixture of 2,4-dihydroxy-5-methoxybenzaldehyde (0.34 g, 2 mmol), 2,2-dimethyl-1,3-dioxane-4,6-dione (0.29 g, 2 mmol), glycine (0.05 g, 0.7 mmol) and 5 mL ethanol was heated to reflux for 2.5 h. After the reaction was complete, the mixture was cooled to room temperature and then poured into ice-cold water. The resulting solid product was filtered, washed with water and recrystallized (in methanol) to obtain the pure product 7-hydroxy-6-methoxy-2-oxo-2H-chrom-ene-3-carboxylic acid, which had the following characteristics: green solid, yield 92.5%, m.p. 188–190 °C, ^1^H NMR (500 MHz, DMSO-*d*_6_): *δ* 12.82 (s, 1H), 10.91 (s, 1H), 8.67 (s, 1H), 7.44 (s, 1H), 6.82 (s, 1H), and 3.83 (s, 3H).

#### 3.2.3. Preparation of 7-hydroxy-6-methoxy-2H-chromen-2-one (**4**) [26]

7-Hydroxy-6-methoxy-2-oxo-2H-chrom-ene-3-carboxylic acid (**3**, 0.47 g, 2 mmol) was heated to reflux in a mixture of 4 mL of ethylene glycol and 5 mL of pyridine for 3.5 h. After cooling to room temperature, the reaction mixture was acidified to pH = 4 using HCl (10 M). The acid mixture was then extracted with CH_2_Cl_2_ (50 mL × 3), dried with Na_2_SO_4_, and evaporated to yield the product, which was recrystallized from ethanol. The product had the following characteristics: yellow solid, yield 67.9%, m.p. 204–206 °C, ^1^H NMR (DMSO-*d*_6_): δ 10.3 (br, 1H), 7.88 (d, 1H, *J* = 8.0 Hz), 7.19 (s, 1H), 6.77 (s, 1H), 6.20 (d, 1H, *J* = 8.0 Hz), and 3.81 (s, 3H).

#### 3.2.4. Preparation of 3-(4-fluorophenyl)-7-hydroxy-6-methoxy-2H-chromen-2-one (**5**) [27]

2,4-dihydroxy-5-methoxybenzaldehyde (0.34 g, 2 mmol) was dissolved in dry acetone (10 mL), and 4-fluorophenylacetyl chloride (0.38 g, 2.2 mmol) was then added dropwise. The mixture was heated to reflux with anhydrous K_2_CO_3_ (1.10 g, 8.0 mmol) for 4 h. Acetone was then removed under reduced pressure, and ice water (20 mL) was added. The resulting mixture was extracted with EtOAc (100 mL × 3). The EtOAc layers were combined, dried (Na_2_SO_4_), and evaporated under reduced pressure to give the crude product. The crude solid was recrystallized from methanol, resulting in the corresponding 3-(4-fluorophenyl)-7-hydroxy-6-methoxy-2H-chromen-2-one with the following characteristics: yellow solid, yield 82.4%, m.p. 205–208 °C, ^1^H NMR (500 MHz, CDCl_3_): δ 7.71–7.65 (m, 3H), 7.11 (t, *J* = 8.7 Hz, 2H), 6.94 (s, 1H), 6.90 (s, 1H), 6.24 (s, 1H), and 3.96 (s, 3H).

#### 3.2.5. Preparation of 3-acetyl-7-hydroxy-6-methoxy-2H-chromen-2-one (**6**) [28]

To a cold mixture of 2,4-dihydroxy-5-methoxybenzaldehyde (0.34 g, 2 mmol) and ethyl acetoacetate (0.26 g, 2 mmol), 2 mL piperidine was added by rapid stirring. After 30 min, the yellowish precipitate was filtered and subsequently washed with EtOH. This precipitate had the following characteristics: yellow solid, yield 46.8%, m.p. 118–120 °C, ^1^H NMR (500 MHz, DMSO-*d*_6_): δ 9.64 (s, 1H), 8.57 (s, 1H), 7.23 (s, 1H), 7.10 (s, 1H), 3.92 (s, 3H), and 2.55 (s, 3H).

#### 3.2.6. Preparation of 3-(4-bromoaniline formyl)-7-hydroxy-6-methoxy-2H-chromen-2-one (**7**) [29]

HATU (0.27 g, 0.72 mmol) was added to a solution of 7-hydroxy-6-methoxy-2-oxo-2H-chrom-ene-3-carboxylic acid (0.11 g, 0.48 mmol) in anhydrous DMF at 0 °C under nitrogen. The resulting solution was stirred at 0 °C for half an hour. Thereafter, the corresponding *p*-bromoaniline (0.083 g, 0.48 mmol) and DIPEA (0.19 g, 1.4 mmol) were added, and the resulting solution incubated at room temperature overnight while stirring. Once the completion of the reaction was verified by TLC, the reaction mixture was poured into ice water and extracted twice with ethyl acetate (100 mL). The ethyl acetate layers were combined, washed with brine, dried over sodium sulfate and subjected to rotary evaporation under reduced pressure. This process yielded a product with the following characteristics: orange solid, yield 89.6%, m.p. 247–249 °C, ^1^H NMR (500 MHz, DMSO-*d*_6_): δ 10.78 (d, *J* = 10.4 Hz, 1H), 8.82 (s, 1H), 7.68 (d, *J* = 8.8 Hz, 2H), 7.55–7.49 (m, 3H), 6.89 (s, 1H), and 3.85 (s, 3H).

#### 3.2.7. General Procedure for the Synthesis of Compounds **4a**–**4j**, **5a**–**5j**, **6d**, **6h**, **7a**, and **7d** [30]

Benzenesulfonyl chloride with different substituents (**a**–**j**, 3.0 mmol) was added dropwise to a solution of compounds **4**–**7** (1.0 mmol) in 5 mL of anhydrous pyridine. The reaction mixture was heated to reflux for 2 h, cooled to room temperature, poured into ice water and acidified with HCl (10 M). The resulting precipitate was collected by filtration and then purified by flash silica column chromatography using EA/PE as the eluent. As a result, compounds **4a**–**4j**, **5a**–**5j**, **6d**, **6h**, **7a**, and **7d** were prepared via a similar method.

**Figure 4:**
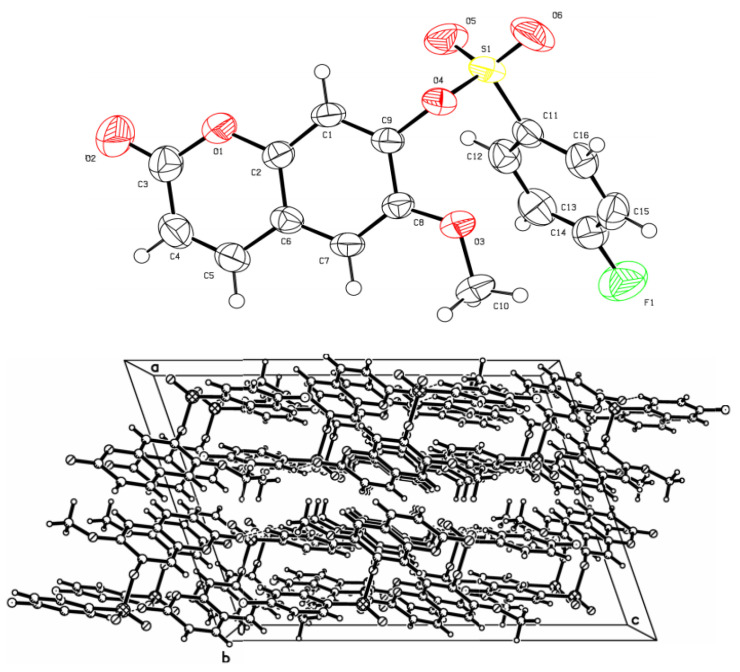
The X-ray diffraction structure of target compound **4c** (CCDC number: 2020935).

The ^1^H and ^13^C NMR spectra and the HRESIMS data of the targeted compounds are detailed in the Supporting Materials. The X-ray diffraction structure of target compound **4c** is shown in Figure 4, and the Cambridge Crystallographic Data Centre (CCDC) number is 2020935.

### 3.3. Acaricidal Activities against T. cinnabarinus

An acaricidal activity assay of the test compounds against mites was conducted according to procedures outlined in the literature [31]. Each title compound (10 mg) was dissolved in 2 mL DMSO to give a 5 mg/mL stock solution. The stock solution was then diluted to a series of working concentrations with 1% Tween 80 to obtain the test solutions. Subsequently, the leaves of two-leaf-stage broad beans were inoculated with 150–200 female adult *T. cinnabarinus* mites and reared in a greenhouse (26 ± 1 °C, 75–80% RH, 16 h light and 8 h dark alternatively) for 8 h. The test solution of the compound was then evenly sprayed on these leaves, which were then cultivated in the greenhouse for 72 h. In the blank control group, Tween 80 (1%) was used instead of the treatment solution. Cyetpyrafen (CAS NO. 1253429-01-4) was used as a positive control. Each treatment was repeated three times. The dead insects were recorded under a stereoscope, and the corrected mortalities of mites treated with the compounds were calculated. Toxicity regression equations with a correlation coefficient and lethal concentration that killed *T. cinnabarinus* by 50% (LC_50_) were obtained using toxicity regression equation software. The experimental data are detailed in the Supplementary Materials (Supporting Information, Table S1).

### 3.4. Toxicity to Artemia

The toxicity of the title compounds to the brine shrimp *A. salina* was evaluated according to the reported method [32]. Each title compound (10 mg) was dissolved in 1 mL of DMSO to give a 10 mg/mL stock solution, which was then diluted to a series of working concentrations with 1% Tween 20—artificial seawater (5%). Subsequently, approximately 50 hatched (10 h instar) brine shrimp were transferred into each pore of a 24-well plate filled with the test solutions. Finally, the brine shrimp were reared in an illumination incubator (28 ± 1 °C, 12 h light and 12 h dark alternatively) for 24 h. DMSO—1% Tween 20 was used as a blank control. Each treatment was conducted in triplicate. The dead insects were recorded under a stereoscope, and the corrected mortalities of *A. salina* treated with the compounds were calculated. Toxicity regression equations with a correlation coefficient and lethal concentration that killed brine shrimp by 50% (LC_50_) were obtained using toxicity regression equation software. The experimental data are detailed in the Supplementary Materials (Supporting Information, Table S2).

### 3.5. AChE Inhibition

The effects of the title compounds on the in vitro AChE activity were evaluated using ELISA technology [23]. AChE derived from electric eels was purchased from Sigma–Aldrich Chemie GmbH (Buchs, Switzerland). Briefly, the AChE was preincubated with different concentrations (500, 250, 125, 62.5, 31.25, 15.625, and 7.8125 μg/mL) of the compounds in PBS solution. Then, the AChE activities after incubation were measured using the corresponding ELISA kit according to the manufacturer’s instructions (Jiancheng, Nanjing, China). The detection was performed using a multifunctional microplate reader (SpectraMax^®^ M2e, Thermo Scientific, Waltham, MA, USA). The experimental data are detailed in the Supplementary Materials (Supporting Information, Table S1).

### 3.6. Molecular Docking

Molecular docking was studied on the Yinfo Cloud Platform (http://cloud.yinfotek.com, accessed on 25 March 2022). The chemical structures of compounds **4j**, **5a**, and **5j** were drawn by JSME and converted to 3D structures with energy minimization using the MMFF94 force field. A crystal structure of *Anopheles gambiae* AChE in complex with the AChE inhibitor PRC1214 (PDB accession no. 6ARY) was automatically downloaded from the RCSB Protein Data Bank (http://www.rcsb.org/, accessed on 25 March 2022) [33]. All redundant atoms except chain A were deleted, and the protein structure was then carefully treated in several steps, including residue repair, protonation, and partial charge assignment, with the AMBER ff14SB force field. The DMS tool was employed to build the molecular surface of the receptor using a probe atom with a 1.4 Å radius. The binding pocket was defined by the crystal ligand, and spheres were created to fill the site by employing the Sphgen module in UCSF Chimera. A box enclosing the spheres was set with its center at (−59.984, 57.429, 17.731) and dimensions of (36.137, 28.151, 32.027), within which grids necessary for rapid score evaluation were created by the grid module. Finally, the DOCK 6.7 program was utilized to execute semiflexible docking, where 10,000 different orientations were produced. A clustering analysis was performed (RMSD threshold was set 2.0 Å) for candidate poses, and the best scored poses were output. The experimental data are detailed in the Supplementary Materials (Supporting Information, Tables S3–S5).

## 4. Conclusions

**Figure 5:**
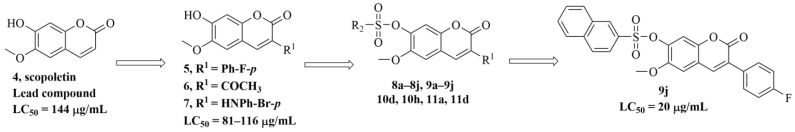
Molecular structure modification roadmap for insecticidal activity.

As described above, several scopoletin analogs synthesized by introducing various substitutes at the C3 and substituted-phenyl sulfate groups at the C7 of the coumarin skeletal lead were acaricidally active against *T. cinnabarinus*. Acetyl, 4-fluorophenyl, and 4-bromoaniline formyl at the C3 of scopoletin could all improve the acaricidal activity, but the effect of substitution at the C7 of scopoletin depended on the phenyl sulfate group. Specifically, 3-(4-F-phenyl) and 7-(naphthalene-2-sulfonate) seem to be the best substitutes for the acaricidal activity of scopoletin, which may be attributed to their large volume. Most of the target compounds exhibited moderate to strong inhibition of mite AChE in vitro, and the degree of AChE inhibition was consistent with acaricidal activity. Molecular docking showed that compounds with higher acaricidal activity could conjugate more tightly with the target protein AChE by binding more main amino acid residues at the active site. The above results imply that AChE may be one main action site of the title compounds. The present data show that this design idea (Figure 5) is feasible and provides a reference for later structural modification. It is worth mentioning that this study has been granted Chinese patents (No. CN 110590725 A).

## Data Availability

Not applicable.

## Supplementary Materials

The following supporting information can be downloaded at: https://www.mdpi.com/article/10.3390/molecules28020530/s1.

## Sample Availability

Samples of the compounds are not available from the authors.
