# Supplementary material for: Design and Synthesis of Scopoletin Sulfonate Derivatives as Potential Insecticidal Agents
Source: Molecules. 2023 Jan 5;28(2):530. doi: 10.3390/molecules28020530 (PMC9865501; doi:10.3390/molecules28020530)
Supplement: Supplementary file 1 [file molecules-28-00530-s001.zip › molecules-2083348-supplementary.pdf]

Supporting Information

Design and synthesis of scopoletin sulfonate derivatives as potential insecticidal agents

Congmin Liu[#], Pan-Yuan Zheng[#], Hong-Mei Wang[#], Yan Wei, Chuan-Ping Wang, Shuang-Hong Hao*

Research Center of Bio-Pesticides Engineering & Tech. of Shandong Province, College of Chemistry and Pharmaceutical Sciences, Qingdao Agricultural University, Qingdao 266109, China;

[#] These authors contributed equally to this work.

* Correspondence to: Shuang-Hong Hao, hsh@qau.edu.cn; Tel.: +86-532-8803-0522

Table of Contents

1. Physical and Chemical Properties Data of the Synthesized Compounds…………………………….3

2. NMR Spectra and MS of Synthesized Compounds……………………………………………….9

3. Acaricidal activities………………………………………………………………………………..44

4. Lethal activities of title compounds to Artemia……………………………………………….….45

5. The molecular docking scores of compounds and the acetylcholinesterase………………………46

Characterization of products 8-11.

6-methoxy-2-oxo-2H-chromen-7-yl-benzenesulfonate (4a).

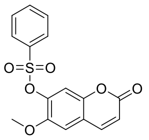

White solid; yield 74.2%; mp:201-203 °C; ^{1}H NMR (500 MHz, $CDCl_3$) δ 7.91 (d, J = 8.6 Hz, 2H), 7.71 (t, J = 7.5 Hz, 1H), 7.62 (d, J = 9.6 Hz, 1H), 7.56 (t, J = 7.9 Hz, 2H), 7.09 (s, 1H), 6.89 (s, 1H), 6.42 (d, J = 9.6 Hz, 1H), 3.66 (s, 3H); ^{13}C NMR (125 MHz, $CDCl_3$) δ 160.13, 149.12, 147.78, 142.42, 140.92, 135.94, 134.45, 129.11, 128.51, 117.80, 117.28, 112.83, 109.87, 56.26; HRMS (ESI): calcd for $C_{16}H_{13}O_6S$ ($[M+H]^+$), 333.0427, found, 333.0424.

6-methoxy-2-oxo-2H-chromen-7-yl-4-methoxybenzenesulfonate (4b).

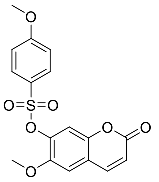

White solid; yield 77.5%; mp:180-183 °C; ^{1}H NMR (500 MHz, $CDCl_3$) δ 7.74 (d, J = 9.0 Hz, 2H), 7.54 (d, J = 9.6 Hz, 1H), 6.98 (s, 1H), 6.92 (d, J = 9.0 Hz, 2H), 6.82 (s, 1H), 6.33 (d, J = 9.6 Hz, 1H), 3.82 (s, 3H), 3.64 (s, 3H); ^{13}C NMR (125 MHz, $CDCl_3$) δ 164.36, 160.26, 149.23, 147.74, 142.54, 141.06, 130.84, 126.99, 117.66, 117.14, 114.31, 112.69, 109.86, 56.35, 55.82; HRMS (ESI): calcd for $C_{17}H_{15}O_7S$ ($[M+H]^+$), 363.0533, found, 363.0535.

6-methoxy-2-oxo-2H-chromen-7-yl-4-fluorobenzenesulfonate (4c).

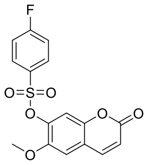

Yellow solid; yield 66.9%; mp:173-175 °C; ^{1}H NMR (500 MHz, DMSO-d_6) δ 8.03 – 7.92 (m, 3H), 7.52 (t, J = 8.8 Hz, 2H), 7.43 (s, 1H), 7.30 (s, 1H), 6.53 (d, J = 9.6 Hz, 1H), 3.55 (s, 3H); ^{13}C NMR (125 MHz, DMSO-d_6) δ 165.31 (d, J = 252.9 Hz), 160.06, 148.52, 147.50, 143.88, 139.82, 132.24 (d, J = 10.4 Hz), 131.21 (d, J = 3.2 Hz), 118.78, 117.44, 117.36 (d, J = 21.5 Hz), 112.70, 111.70, 56.53; HRMS (ESI): calcd for $C_{16}H_{12}FO_6S$ ($[M+H]^+$), 351.0333, found, 351.0337.

6-methoxy-2-oxo-2H-chromen-7-yl-4-chlorobenzenesulfonate (4d).

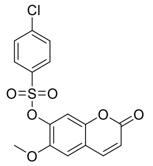

White solid; yield 66.9%; mp:164-165 °C; ^{1}H NMR (500 MHz, $CDCl_3$) δ 7.76 (d, J = 8.7 Hz, 2H), 7.56 (d, J = 9.6 Hz, 1H), 7.45 (d, J = 8.7 Hz, 2H), 7.08 (s, 1H), 6.83 (s, 1H), 6.35 (d, J = 9.6 Hz, 1H), 3.59 (s, 3H); ^{13}C NMR (125 MHz, $CDCl_3$) δ 160.08, 148.81, 147.71, 142.47, 141.27, 140.50, 134.19, 129.96, 129.41, 117.98, 117.37, 112.86, 109.94, 56.19; HRMS (ESI): calcd for $C_{16}H_{12}ClO_6S$ ($[M+H]^+$), 367.0037. found, 367.0038.

6-methoxy-2-oxo-2H-chromen-7-yl-4-bromobenzenesulfonate (4e).

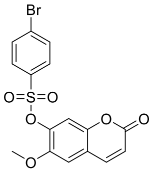

Yellow solid; yield 82.3%; mp:188-190 °C; ^{1}H NMR (500 MHz, $CDCl_3$) δ 7.76 (s, 2H), 7.70 (d, J = 8.6 Hz, 2H), 7.63 (d, J = 9.6 Hz, 1H), 7.16 (s, 1H), 6.90 (s, 1H), 6.44 (d, J = 9.6 Hz, 1H), 3.67 (s, 3H); ^{13}C NMR (125 MHz, $CDCl_3$) δ 160.01, 148.88, 147.81, 142.32, 140.63, 134.91, 132.41,

129.97, 129.85, 117.95, 117.45, 112.91, 109.93, 56.23; HRMS (ESI): calcd for $C_{16}H_{12}BrO_6S$ ($[M+H]^+$), 412.9512 found, 412.9510.

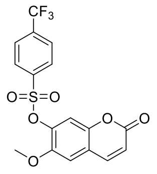

6-methoxy-2-oxo-2H-chromen-7-yl-4-(trifluoromethyl)-benzenesulfonate (4f). White solid; yield 81.5%; mp:169-171 °C; ^{1}H NMR (500 MHz, $CDCl_3$) δ 8.06 (d, *J* = 8.2 Hz, 2H), 7.84 (d, *J* = 8.3 Hz, 2H), 7.62 (d, *J* = 9.6 Hz, 1H), 7.21 (s, 1H), 6.89 (s, 1H), 6.45 (d, *J* = 9.6 Hz, 1H), 3.62 (s, 3H); ^{13}C NMR (125 MHz, $CDCl_3$) δ 159.91, 148.71, 147.85, 142.22, 140.44, 139.58, 135.98(q, *J* = 34.5 Hz), 129.12, 126.18(q, *J* = 3.59 Hz), 123.12 (q, *J* = 274.8 Hz), 118.08, 117.61, 113.07, 109.87, 56.09; HRMS (ESI): calcd for $C_{17}H_{12}F_3O_6S$ ($[M+H]^+$), 401.0301, found, 401.0300.

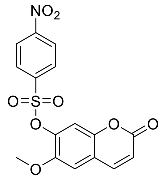

6-methoxy-2-oxo-2H-chromen-7-yl-4-nitrobenzenesulfonate (4g).
Yellow solid; yield 68.3%; mp:178-181 °C; ^{1}H NMR (500 MHz, DMSO-d_6) δ 8.46 (d, *J* = 8.9 Hz, 2H), 8.16 (d, *J* = 8.9 Hz, 2H), 8.00 (d, *J* = 9.6 Hz, 1H), 7.45 (s, 1H), 7.39 (s, 1H), 6.55 (d, *J* = 9.6 Hz, 1H), 3.52 (s, 3H); ^{13}C NMR (125 MHz, DMSO-d_6) δ 160.00, 151.56, 148.38, 147.55, 143.82, 140.56, 139.68, 130.52, 125.18, 119.03, 117.58, 112.75, 111.90, 56.55; HRMS (ESI): calcd for $C_{16}H_{12}NO_8S$ ($[M+H]^+$), 378.0278, found, 378.0277.

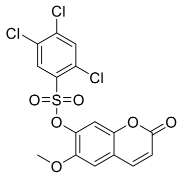

6-methoxy-2-oxo-2H-chromen-7-yl-2,4,5-trichlorobenzenesulfonate (4h). White solid; yield 66.1%; mp:186-188 °C; ^{1}H NMR (500 MHz, $CDCl_3$) δ 7.97 (s, 1H), 7.69 (s, 1H), 7.56 (d, *J* = 9.6 Hz, 1H), 7.08 (s, 1H), 6.88 (s, 1H), 6.37 (d, *J* = 9.6 Hz, 1H), 3.66 (s, 3H); ^{13}C NMR (125 MHz, $CDCl_3$) δ 159.95, 148.79, 147.79, 142.29, 140.50, 139.67, 134.09, 133.33, 132.81, 132.32, 131.83, 118.21, 117.65, 113.00, 110.13, 56.36; HRMS (ESI): calcd for $C_{16}H_9Cl_3O_6S$ ($[M+H]^+$), 436.9229, found, 436.9228.

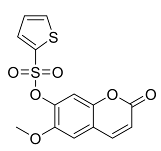

6-methoxy-2-oxo-2H-chromen-7-yl-thiophene-2-sulfonate (4i).
White solid; yield 81.2%; mp:188-189 °C; ^{1}H NMR (500 MHz, DMSO-d_6) δ 8.26 (d, *J* = 4.9 Hz, 1H), 8.00 (d, *J* = 9.6 Hz, 1H), 7.83 (d, *J* = 3.8 Hz, 1H), 7.45 (s, 1H), 7.34 – 7.28 (m, 1H), 7.23 (s, 1H), 6.54 (d, *J* = 9.6 Hz, 1H), 3.64 (s, 3H); ^{13}C NMR (125 MHz, DMSO-d_6) δ 160.03, 148.80, 147.40, 143.82, 139.90, 137.88, 137.14, 133.72, 128.77, 118.79, 117.41, 112.44, 111.63, 56.68; HRMS (ESI): calcd for $C_{14}H_{11}O_6S_2$ ($[M+H]^+$), 338.9991, found, 338.9995.

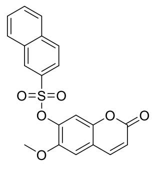

6-methoxy-2-oxo-2H-chromen-7-yl-naphthalene-2-sulfonate (4j).
White solid; yield 57.2%; mp:164-166 °C; ^{1}H NMR (500 MHz, $CDCl_3$) δ 8.42 (s, 1H), 8.04 – 7.85 (m, 4H), 7.65 (m, *J* = 24.7, 15.8, 8.5 Hz, 3H), 7.14 (s, 1H), 6.85 (s, 1H), 6.38 (d, *J* = 9.5 Hz,

1H), 3.52 (s, 3H);[13] C NMR (125 MHz, $CDCl_3$) δ 160.13, 149.02, 147.74, 142.49, 140.90, 135.53, 132.66, 131.73, 130.44, 129.83, 129.43, 129.36, 128.03, 127.99, 122.91, 117.81, 117.17, 112.81, 109.93, 56.14; HRMS (ESI): calcd for $C_{20}H_{15}O_6S$ ($[M+H]^+$), 383.0584, found, 383.0586.

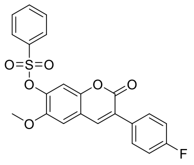

3-(4-fluorophenyl)-6-methoxy-2-oxo-2H-chromen-7-yl-benzenesulfonat-e (5a). White solid; yield 88.4%; mp:129-130 °C; ^{1}H NMR (500 MHz, DMSO-d_6) δ 8.15 (s, 1H), 7.87 (dd, *J* = 11.2, 10.3 Hz, 3H), 7.75 (dd, *J* = 10.6, 3.6 Hz, 2H), 7.69 (t, *J* = 7.8 Hz, 2H), 7.43 (s, 1H), 7.32 (t, *J* = 8.8 Hz, 2H), 7.27 (s, 1H), 3.53 (s, 3H); ^{13}C NMR (125 MHz, DMSO-d_6) δ 162.79(d, *J* = 248.6 Hz), 159.86, 148.74, 146.92, 140.08, 139.84, 135.61, 135.13, 131.33(d, *J* = 3.16 Hz), 131.19(d, *J* = 9.06 Hz), 130.05, 128.80, 126.93, 119.36, 115.72(d, *J* =21.3 Hz), 112.17, 111.58, 56.51; HRMS (ESI): calcd for $C_{22}H_{16}FO_6S$ ($[M+H]^+$), 427.0646, found, 427.0649.

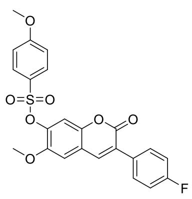

3-(4-fluorophenyl)-6-methoxy-2-oxo-2H-chromen-7-yl-4-methoxybenze-nesulfonate (5b). White solid; yield 74.4%; mp:148-150 °C; ^{1}H NMR (500 MHz, $CDCl_3$) δ 7.81 (d, *J* = 7.5 Hz, 2H), 7.70 (s, 1H), 7.65 (dd, *J* = 7.1, 5.5 Hz, 2H), 7.14 – 7.06 (m, 3H), 7.00 – 6.96 (m, 3H), 3.90 (s, 3H), 3.70 (s, 3H); ^{13}C NMR (125 MHz, $CDCl_3$) δ 164.40, 163.16 (d, *J* = 251.4 Hz),160.11, 149.31, 147.08, 140.74, 138.82, 130.88 (d, *J* = 7.8 Hz), 130.83, 130.44 (d, *J* = 3.5 Hz), 127.53, 126.88, 118.47, 115.53 (d, *J* = 21.7 Hz), 114.33, 112.22, 109.86, 56.30, 55.84; HRMS (ESI): calcd for $C_{23}H_{18}FO_7S$ ($[M+H]^+$), 457.0751, found, 457.0756.

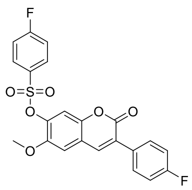

3-(4-fluorophenyl)-6-methoxy-2-oxo-2H-chromen-7-yl 4-fluorobenzenes-ulfonate (5c). Yellow solid; yield 84.1%; mp:155-157 °C; ^{1}H NMR (500 MHz, DMSO-d_6) δ 8.17 (s, 1H), 7.97 (dd, *J* = 8.8, 5.0 Hz, 2H), 7.76 (dd, *J* = 8.7, 5.6 Hz, 2H), 7.53 (t, *J* = 8.8 Hz, 2H), 7.44 (s, 1H), 7.32 (dd, *J* = 14.5, 5.3 Hz, 3H), 3.56 (s, 3H); ^{13}C NMR (125 MHz, DMSO-d_6) δ 166.18 (d, *J* = 260.1 Hz), 162.8 (d, *J* = 247.1 Hz,), 159.85, 148.62, 146.96, 140.07, 139.70, 132.26 (d, *J* = 10.18Hz), 131.39 (d, *J* = 3.2 Hz), 131.32 (d, *J* = 3.2 Hz), 131.19 (d, *J* = 8.75 Hz), 126.98, 119.46, 117.28 (d, *J* = 22.6 Hz), 115.72(d, *J* = 21.65 Hz), 112.37, 111.59, 56.51; HRMS (ESI): calcd for $C_{22}H_{15}F_2O_6S$ ($[M+H]^+$), 445.0556, found, 445.0556.

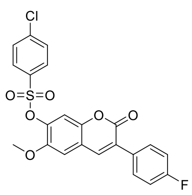

3-(4-fluorophenyl)-6-methoxy-2-oxo-2H-chromen-7-yl-4-chlorobenzenesu-lfonate (5d). White solid; yield 88.4%; mp:137-140 °C; ^{1}H NMR (500 MHz, $CDCl_3$) δ 7.85 (d, *J* = 8.5 Hz, 2H), 7.70 (s, 1H), 7.67 (dd, *J* = 8.4, 5.5 Hz, 2H), 7.54 (d, *J* = 8.5 Hz, 2H), 7.18 (s, 1H), 7.14 (t, *J* = 8.6 Hz, 2H), 6.95 (s, 1H), 3.69 (s, 3H); ^{13}C NMR (125 MHz, $CDCl_3$) δ 163.27 (d, *J* =

246.7 Hz), 159.97, 149.06, 147.18, 141.31, 140.39, 138.50, 134.30, 130.44 (d, J = 8.5 Hz),130.35 (d, J = 3.4 Hz) 129.98, 129.44, 128.01, 118.72, 115.62 (d, J = 21.9 Hz), 112.52, 109.75, 56.23; HRMS (ESI): calcd for $C_{22}H_{15}ClFO_6S$ ($[M+H]^+$), 461.0256, found, 461.0258.

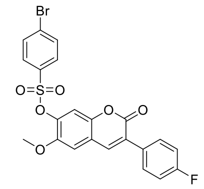

3-(4-fluorophenyl)-6-methoxy-2-oxo-2H-chromen-7-yl-4-bromobenzenes-ulfonate (5e). Yellow solid; yield 63.1%; mp:142-146 °C; ^{1}H NMR (500 MHz, $CDCl_3$) δ 7.77 (d, J = 8.5 Hz, 2H), 7.74 – 7.69 (m, 3H), 7.67 (dd, J = 8.5, 5.5 Hz, 2H), 7.18 (s, 1H), 7.13 (t, J = 8.6 Hz, 2H), 6.95 (s, 1H), 3.69 (s, 3H). (s, 3H); ^{13}C NMR (125 MHz, $CDCl_3$) δ 163.21(d, J = 249.4 Hz), 159.97, 149.04, 147.17, 140.36, 138.51, 134.84, 132.44, 130.44 (d, J = 8.6 Hz), 130.35 (d, J = 3.5 Hz), 129.99, 129.90, 128.01, 118.73, 115.62 (d, J = 21.8 Hz), 112.52, 109.75, 56.23; HRMS (ESI): calcd for $C_{22}H_{15}BrFO_6S$ ($[M+H]^+$), 506.9731, found, 506.9730.

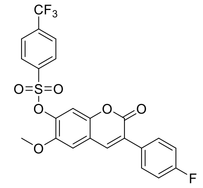

3-(4-fluorophenyl)-6-methoxy-2-oxo-2H-chromen-7-yl4-(trifluoromethyl)-benzenesulfonate (5f).

White solid; yield 78.4%; mp:156-160 °C; ^{1}H NMR (500 MHz, DMSO-d_6) δ 8.17 (s, 1H), 8.11 (d, J = 8.3 Hz, 2H), 8.07 (d, J = 8.4 Hz, 2H), 7.79 – 7.73 (m, 2H), 7.44 (d, J = 3.4 Hz, 2H), 7.32 (t, J = 8.8 Hz, 2H), 3.48 (s, 3H); ^{13}C NMR (125 MHz, DMSO-d_6) δ 162.75 (d, J = 245.2 Hz), 159.82, 148.43, 147.00, 140.00, 139.52, 139.12, 134.83 (q, J =32.4 Hz), 131.29 (d, J =3.1 Hz), 131.19 (d, J =8.4 Hz), 129.93, 127.19 (q, J =3.47 Hz), 127.09, 123.71 (q, J =274.1 Hz), 119.65,115.73 (d, J =21.4 Hz) , 112.57, 111.60, 56.33; HRMS (ESI): calcd for $C_{23}H_{15}F_4O_6S$ ($[M+H]^+$), 495.0519, found, 495.0518.

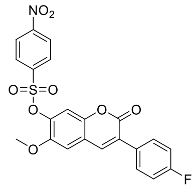

3-(4-fluorophenyl)-6-methoxy-2-oxo-2H-chromen-7-yl-4-nitrobenzenes-ulfonate (5g). White solid; yield 77.9%; mp:150-151 °C; ^{1}H NMR (500 MHz, DMSO-d_6) δ 8.46 (d, J = 8.8 Hz, 2H), 8.17 (d, J = 9.0 Hz, 3H), 7.75 (dd, J = 8.6, 5.6 Hz, 2H), 7.45 (d, J = 12.3 Hz, 2H), 7.32 (t, J = 8.8 Hz, 2H), 3.53 (s, 3H); ^{13}C NMR (125 MHz, DMSO-d_6) δ 162.82 (d, J =245.6 Hz), 159.83, 151.57, 148.47, 147.00, 140.49, 140.01, 139.50, 131.29 (d, J =3.3 Hz), 131.20 (d, J =8.5 Hz), 130.56, 127.13, 125.21, 119.72, 115.75 (d, J =21.6 Hz), 112.47, 111.74, 56.50; HRMS (ESI): calcd for $C_{22}H_{15}FNO_8S$ ($[M+H]^+$), 472.0496, found, 472.0496.

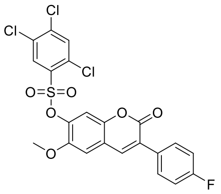

3-(4-fluorophenyl)-6-methoxy-2-oxo-2H-chromen-7-yl-2,4,5-trichlorobe-nzenesulfonate (5h).

White solid; yield 68.7%; mp:197-199 °C; ^{1}H NMR (500 MHz, $CDCl_3$) δ 8.05 (s, 1H), 7.76 (s, 1H), 7.71 (s, 1H), 7.67 (dd, J = 8.6, 5.4 Hz, 2H), 7.17 (s, 1H), 7.13 (t, J = 8.6 Hz, 2H),

7.00 (s, 1H), 3.74 (s, 3H); ^{13}C NMR (125 MHz, $CDCl_3$) δ 163.25 (d, *J* =250.4 Hz), 159.87, 148.95, 147.15, 140.24, 139.69, 138.42, 134.07, 133.35, 132.83, 132.33, 131.83, 130.45 (d, *J* =8.5 Hz), 130.28 (d, *J* =3.5 Hz), 128.18, 118.99, 115.62 (d, *J* =21.4 Hz), 112.58, 110.04, 56.38; HRMS (ESI): calcd for $C_{22}H_{13}Cl_{3}FO_6S$ ($[M+H]^+$), 530.9448, found, 530.9448.

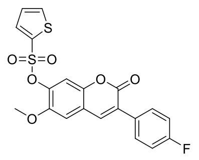

3-(4-fluorophenyl)-6-methoxy-2-oxo-2H-chromen-7-yl-thiophene-2-sulfonate (5i). White solid; yield 76.1%; mp:135-136 °C; ^{1}H NMR (500 MHz, DMSO-d_6) δ 8.25 (d, *J* = 4.9 Hz, 1H), 8.17 (s, 1H), 7.82 (d, *J* = 3.7 Hz, 1H), 7.79 – 7.72 (m, 2H), 7.48 (s, 1H), 7.31 (dd, *J* = 19.6, 11.0 Hz, 4H), 3.63 (s, 3H); ^{13}C NMR (125 MHz, DMSO-d_6) δ 162.71 (d, *J* =246.5 Hz), 159.87, 148.93, 146.89, 140.07, 139.78, 137.98, 137.21, 133.67, 131.26 (d, *J* =3.3 Hz), 131.21 (d, *J* =8.7 Hz), 128.84, 127.04, 119.51, 115.72 (d, *J* =21.7 Hz), 112.14, 111.60, 56.70; HRMS (ESI): calcd for $C_{20}H_{14}FO_6S_2$ ($[M+H]^+$), 433.0210, found, 433.0212.

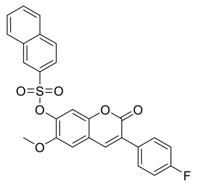

3-(4-fluorophenyl)-6-methoxy-2-oxo-2H-chromen-7-ylnaphthalene-2-sulfonate (5j). White solid; yield 78.8%; mp:140-142 °C; ^{1}H NMR (500 MHz, $CDCl_3$) δ 8.45 (s, 1H), 8.01 (d, *J* = 8.7 Hz, 1H), 7.96 (d, *J* = 8.4 Hz, 2H), 7.91 (d, *J* = 8.7 Hz, 1H), 7.73 – 7.70 (m, 1H), 7.69 – 7.62 (m, 4H), 7.13 (dd, *J* = 17.4, 8.8 Hz, 3H), 6.90 (s, 1H), 3.58 (s, 3H); ^{13}C NMR (125 MHz, $CDCl_3$) δ 163.19 (d, *J* =250.7 Hz), 160.06, 149.27, 147.17, 140.75, 135.56, 132.68, 131.76, 130.48 (d, *J* =7.05 Hz),130.44, 130.41,130.38, 129.83, 129.49, 129.41, 128.00, 128.06, 127.80, 122.95, 118.55, 115.59 (d, *J* =21.2 Hz), 112.46, 109.70, 56.19; HRMS (ESI): calcd for $C_{26}H_{18}FO_6S$ ($[M+H]^+$), 477.0802, found, 477.0800.

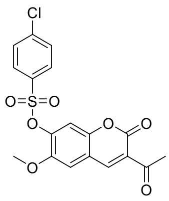

3-acetyl-6-methoxy-2-oxo-2H-chromen-7-yl-4 chlorobenzenesulfonate (6d). White solid; yield 66.2 %; mp:130-133 °C; ^{1}H NMR (500 MHz, $CDCl_3$) δ 8.35 (s, 1H), 7.75 (d, *J* = 8.5 Hz, 2H), 7.47 (d, *J* = 8.5 Hz, 2H), 7.40 (s, 1H), 6.73 (s, 1H), 3.63 (s, 3H), 2.62 (s, 3H); ^{13}C NMR (125 MHz, $CDCl_3$) δ 194.93, 158.94, 157.11, 155.85, 146.93, 141.36, 135.48, 134.12, 129.99, 129.42, 124.51, 122.03, 111.21, 100.54, 56.48, 30.52; HRMS (ESI): calcd for $C_{18}H_{14}ClO_7S$ ($[M+H]^+$), 409.0143, found, 409.0148.

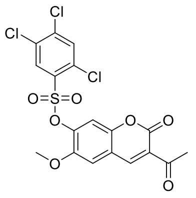

3-acetyl-6-methoxy-2-oxo-2H-chromen-7-yl-2,4,5-trichlorobenzenesulfona-te (6h). White solid; yield 78.3 %; mp:124-128 °C; ^{1}H NMR (500 MHz, $CDCl_3$) δ 8.35 (s, 1H), 7.98 (s, 1H), 7.69 (s, 1H), 7.39 (s, 1H), 6.78 (s, 1H), 3.71 (s, 3H), 2.63 (s, 3H); ^{13}C NMR (125 MHz, $CDCl_3$) δ 194.89, 158.87, 156.97, 155.97, 146.84, 139.74, 135.47, 133.97, 133.31, 132.82, 132.37, 131.89, 124.53, 122.16, 111.28, 100.74, 56.58, 30.52; HRMS (ESI): calcd for $C_{18}H_{12}Cl_{3}O_7S$ ($[M+H]^+$), 478.9335, found, 478.9334.

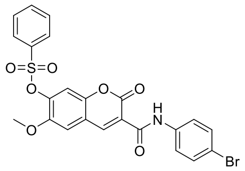

3-((4-bromophenyl)carbamoyl)-6-methoxy-2-oxo-2H-chromen-7-yl- benzenesulfonate (7a). Yellow solid; yield 66.2 %; mp:200-203 °C; ^{1}H NMR (500 MHz, DMSO-d_6) δ 10.75 (s, 1H), 8.86 (s, 1H), 7.98 – 7.89 (m, 3H), 7.77 (d, *J* = 2.26 Hz, 2H), 7.76 (d, *J* = 2.33 Hz, 2H), 7.74 (s, 1H), 7.63 (d, *J* = 8.8 Hz, 2H), 7.48 (s, 1H), 3.60 (s, 3H); ^{13}C NMR (125 MHz, DMSO-d_6) δ 160.45, 165.38, 149.20, 148.14, 146.95, 141.74, 137.69, 135.73, 135.06, 132.31, 130.12, 128.78, 122.42, 120.81, 118.36, 116.56, 112.99, 112.58, 56.70; HRMS (ESI): calcd for $C_{23}H_{16}BrNO_7SNa$ ([M+Na]$^+$), 531.9884, found, 531.9882.

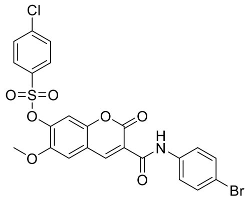

3-((4-bromophenyl)carbamoyl)-6-methoxy-2-oxo-2H-chromen-7-yl-4-chlorobenzenesulfonate (7d). Yellow solid; yield 65.1 %; mp:220-225°C; ^{1}H NMR (500 MHz, DMSO-d_6) δ 10.82 (s, 1H), 8.91 (s, 1H), 7.88 – 7.85 (m, 2H), 7.65 – 7.63 (m, 2H), 7.57 – 7.54 (m, 2H), 7.51 – 7.48 (m, 2H), 7.34 (s, 1H), 7.10 (s, 1H), 3.72 (s, 3H); ^{13}C NMR (125 MHz, DMSO-d_6) δ 160.40, 149.07, 148.21, 146.95, 141.55, 140.80, 137.72, 133.91, 132.32, 130.76, 130.28, 129.14, 122.42, 120.90, 118.53, 116.56, 113.09, 112.80, 56.37; HRMS (ESI): calcd for $C_{23}H_{15}BrClNO_7SNa$ ([M+Na]$^+$), 587.9312, found, 587.9312.

^{1}H NMR, ^{13}C NMR, HRMS data of title compounds 8-11

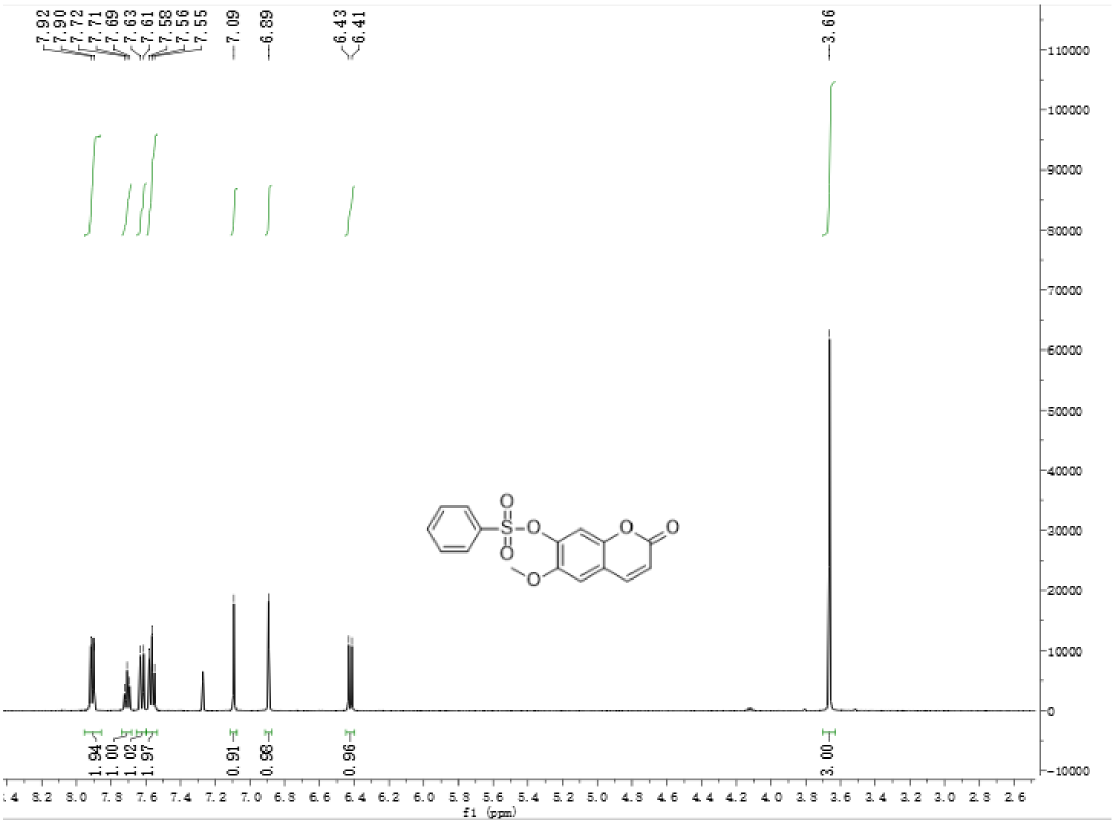

Figure S1 ^{1}H NMR of 4a

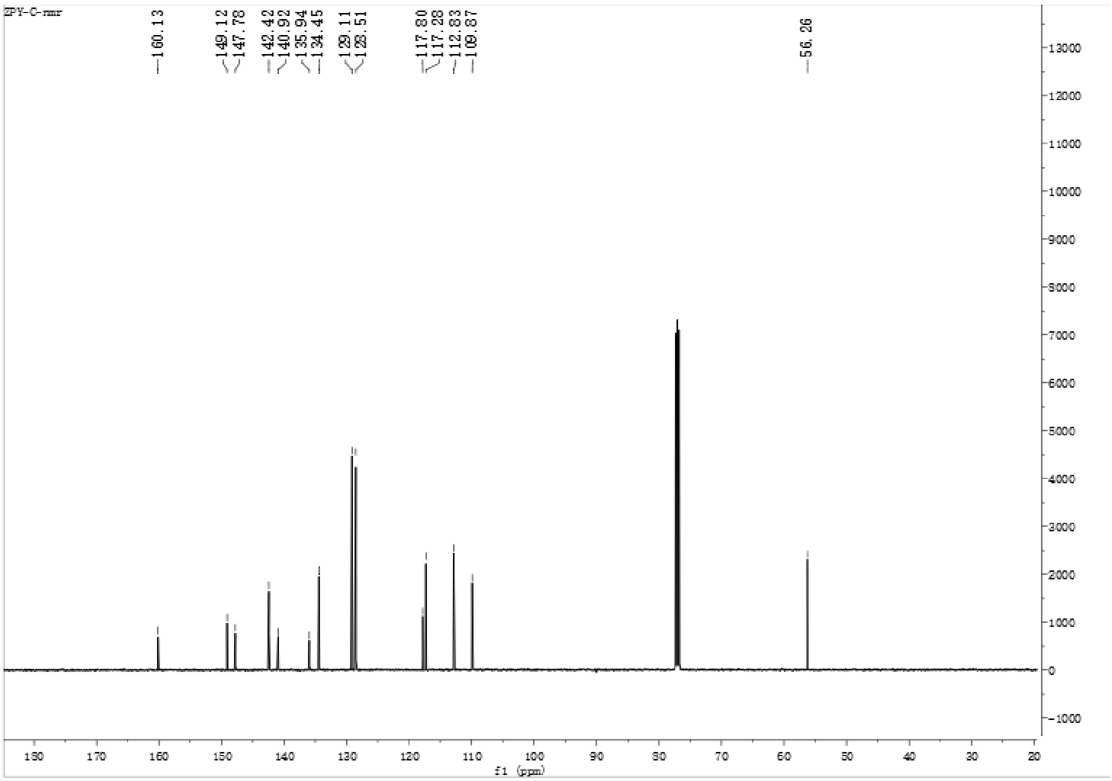

Figure S2 ^{13}C NMR of 4a

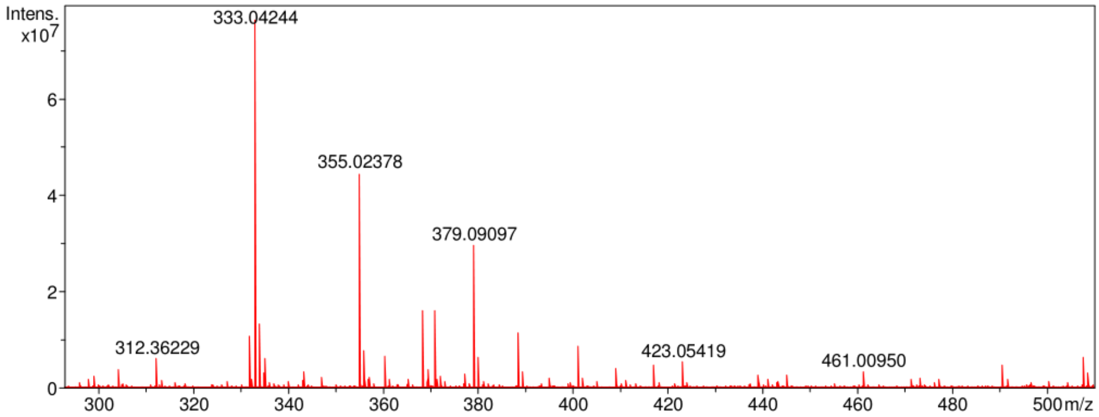

Figure S3 HRMS of 8a

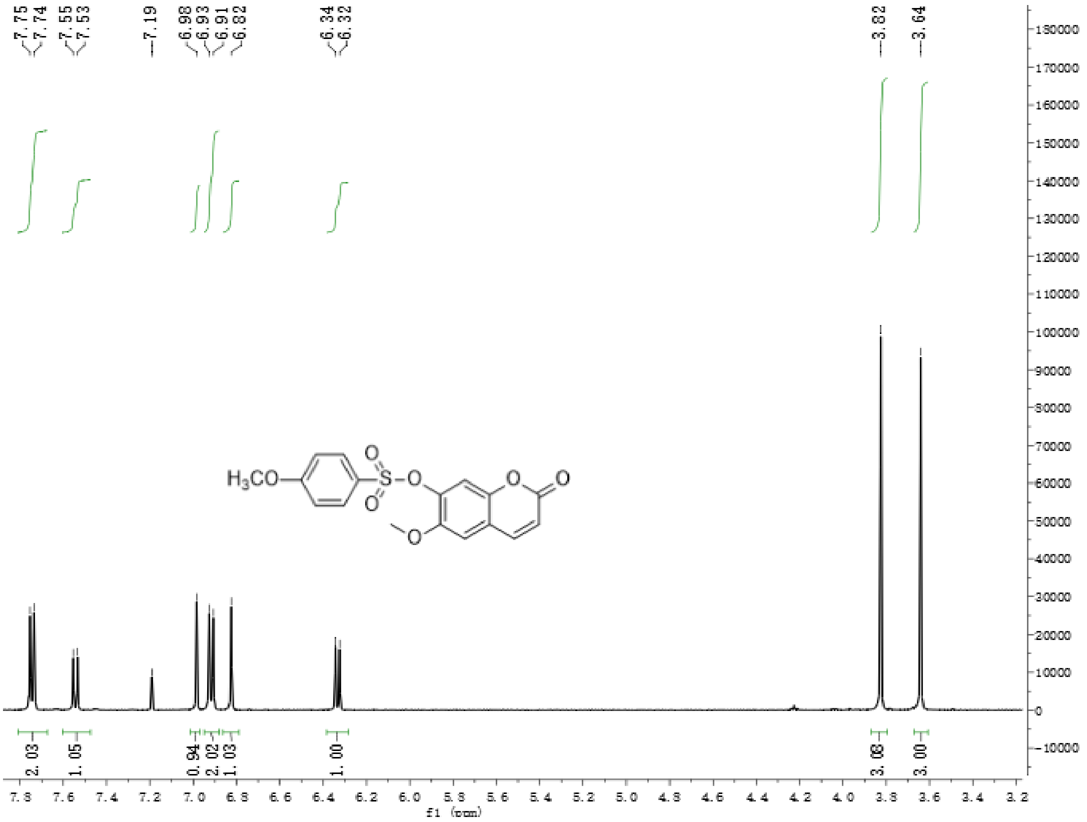

Figure S4 ^{1}H NMR of 8b

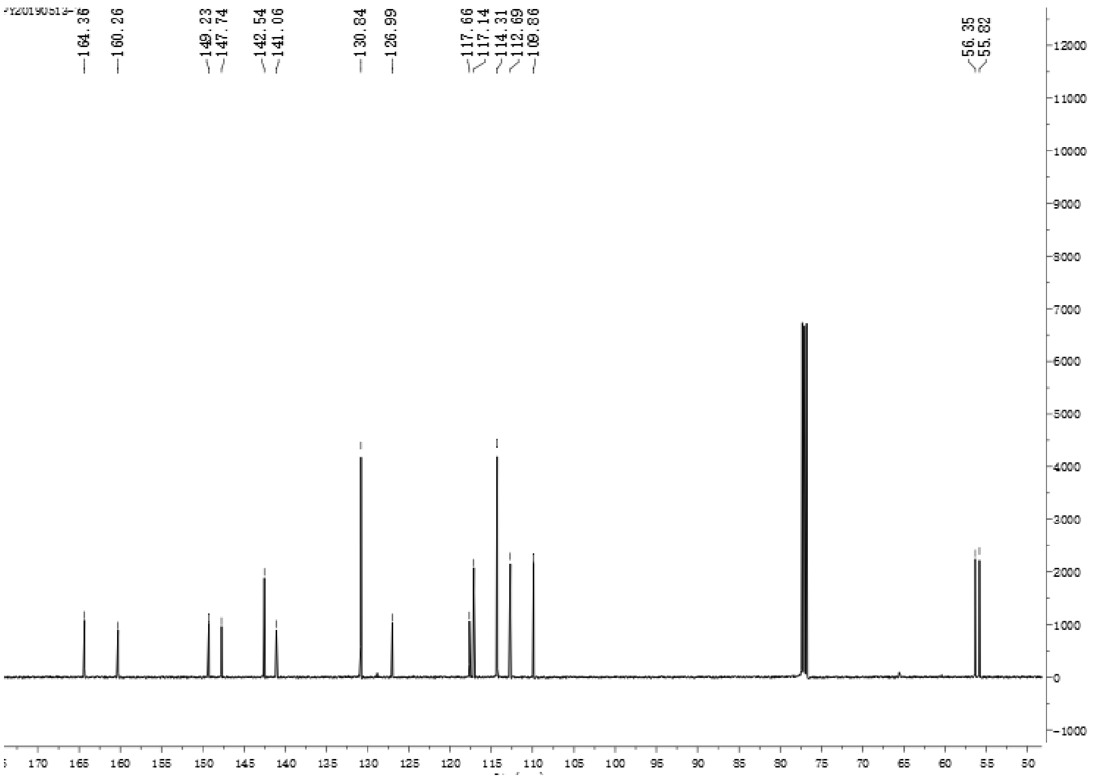

Figure S5 ^{13}C NMR of 8b

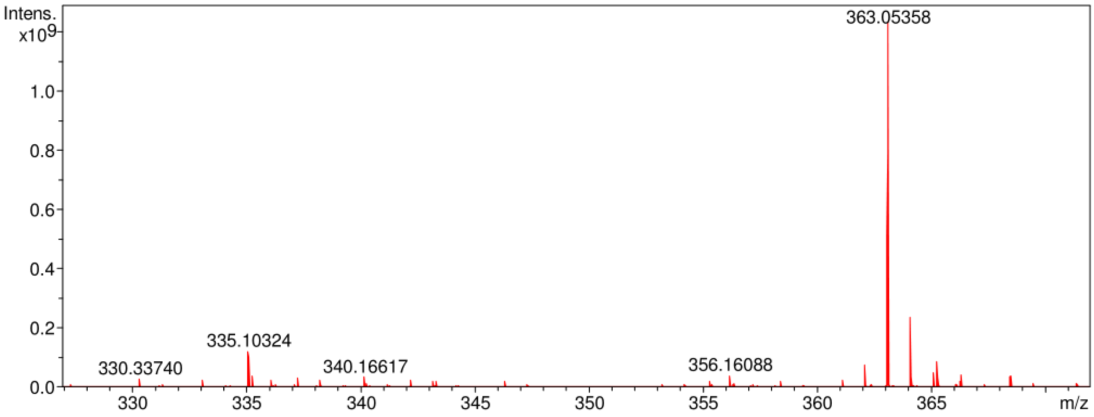

Figure S6 HRMS of 8b

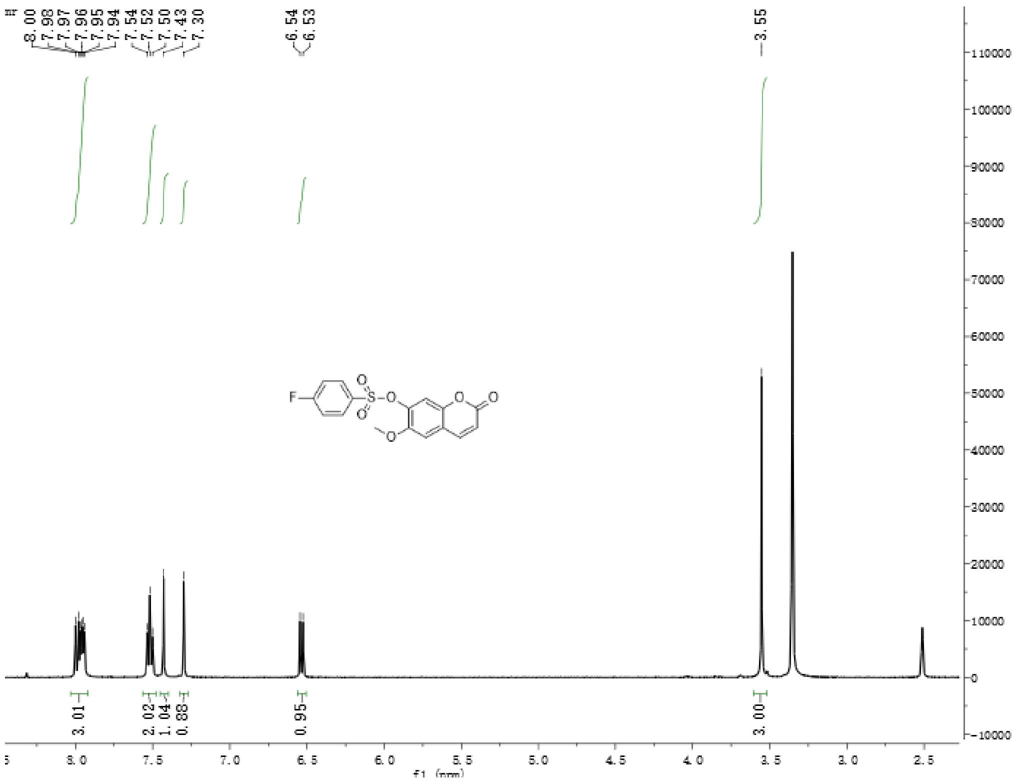

Figure S7 ^{1}H NMR of 8c

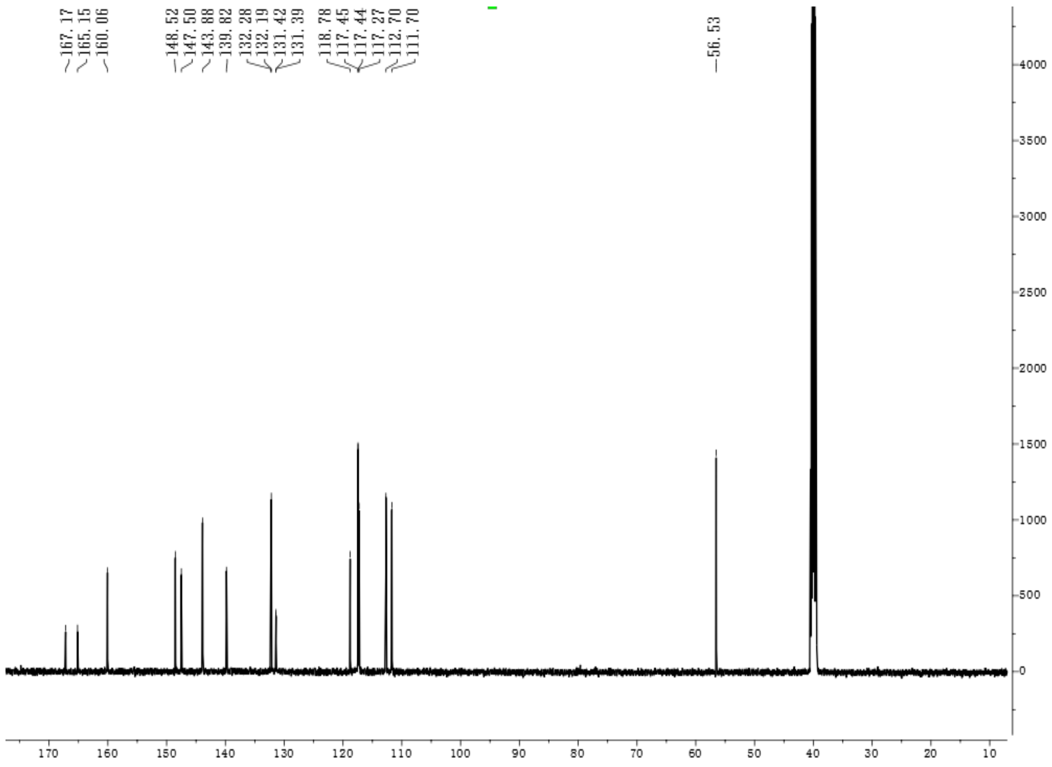

Figure S8 ^{13}C NMR of 8c

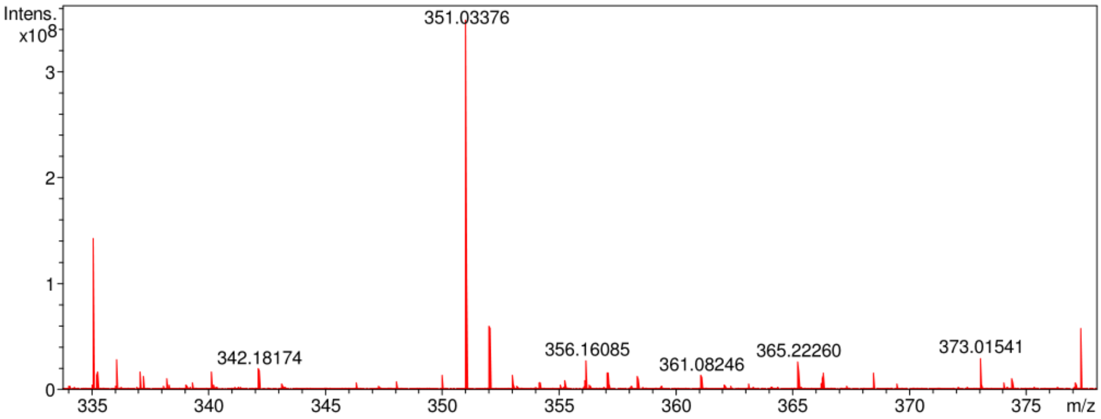

Figure S9　HRMS of 8c

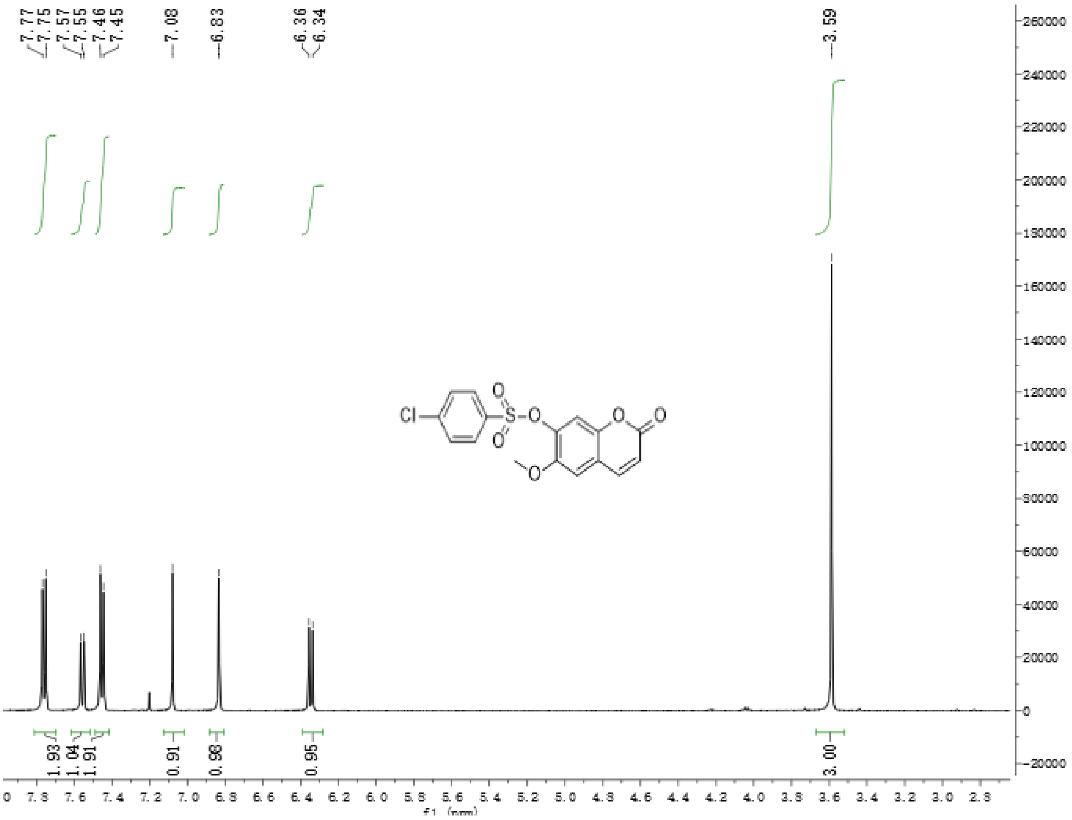

Figure S10　^{1}H NMR of 8d

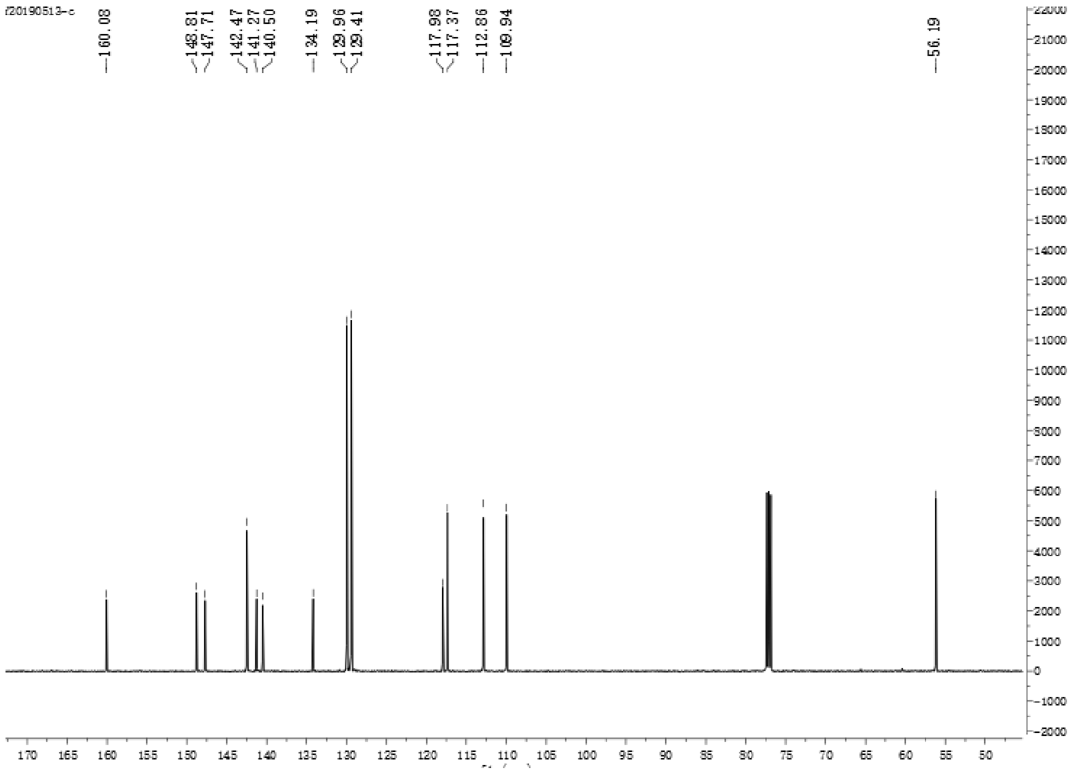

Figure S11 ^{13}C NMR of 8d

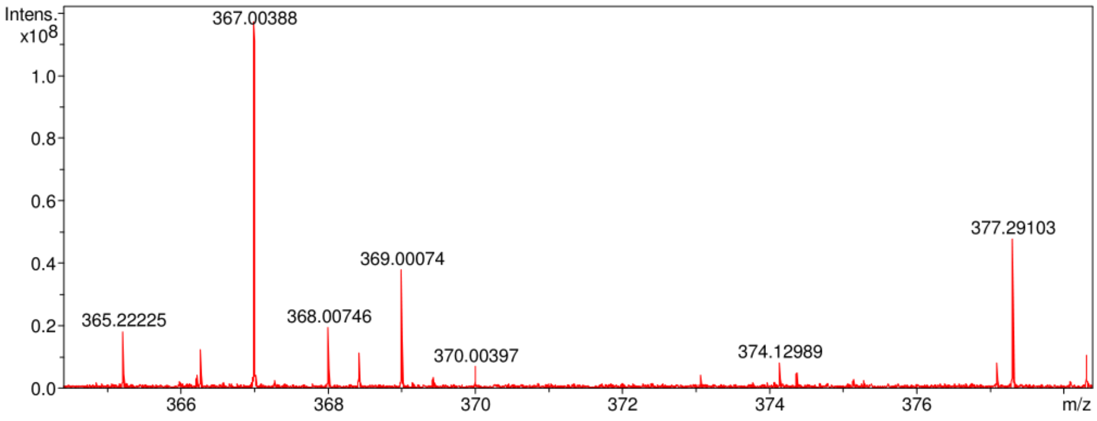

Figure S12 HRMS of 8d

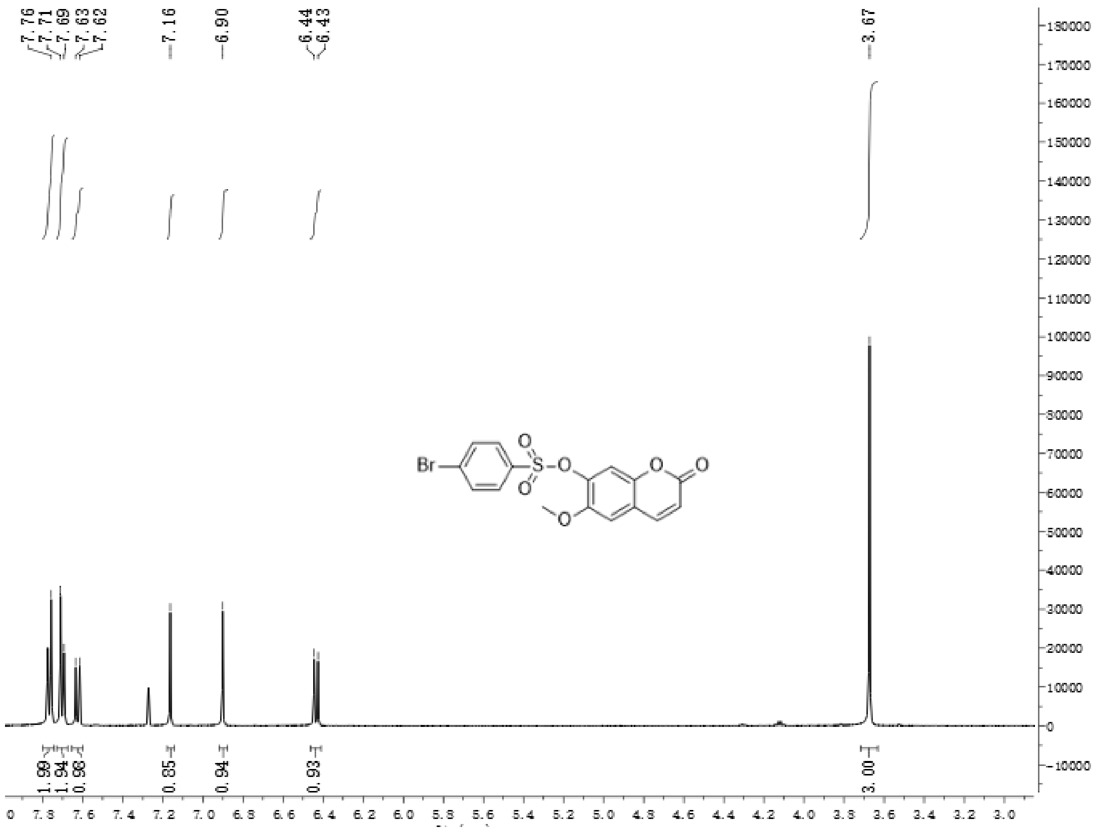

Figure S13 ^{1}H NMR of 8e

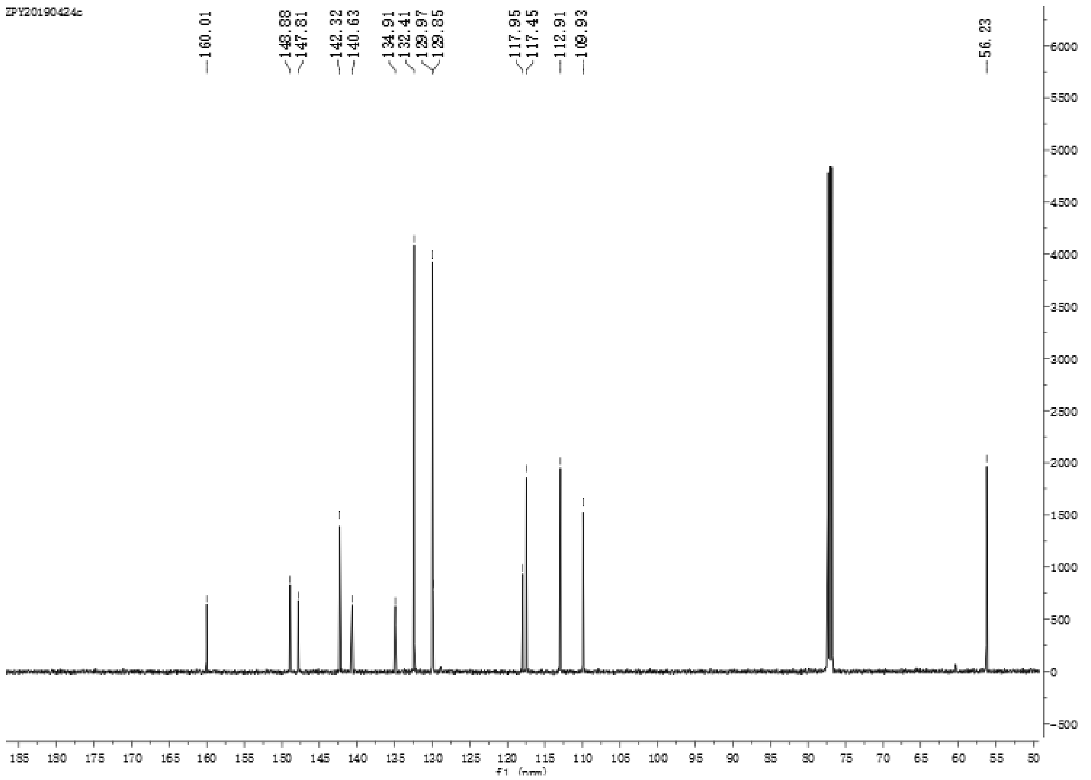

Figure S14 ^{13}C NMR of 8e

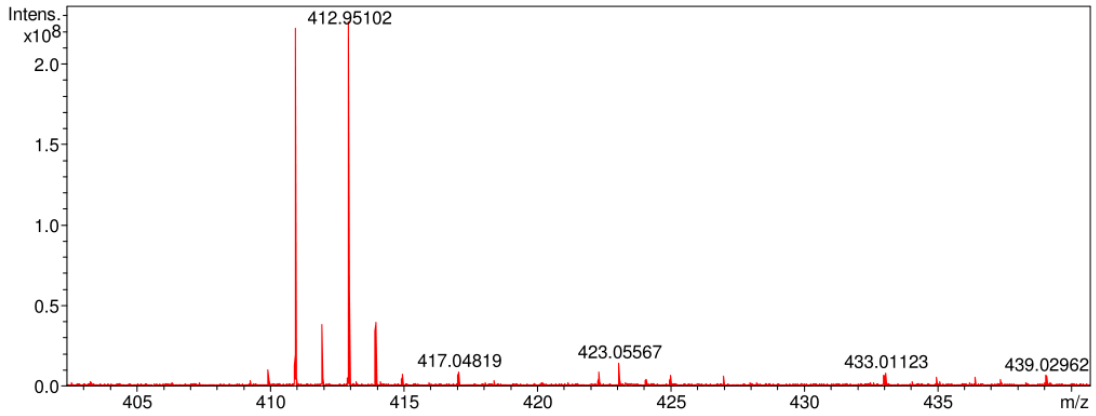

Figure S15 HRMS of 8e

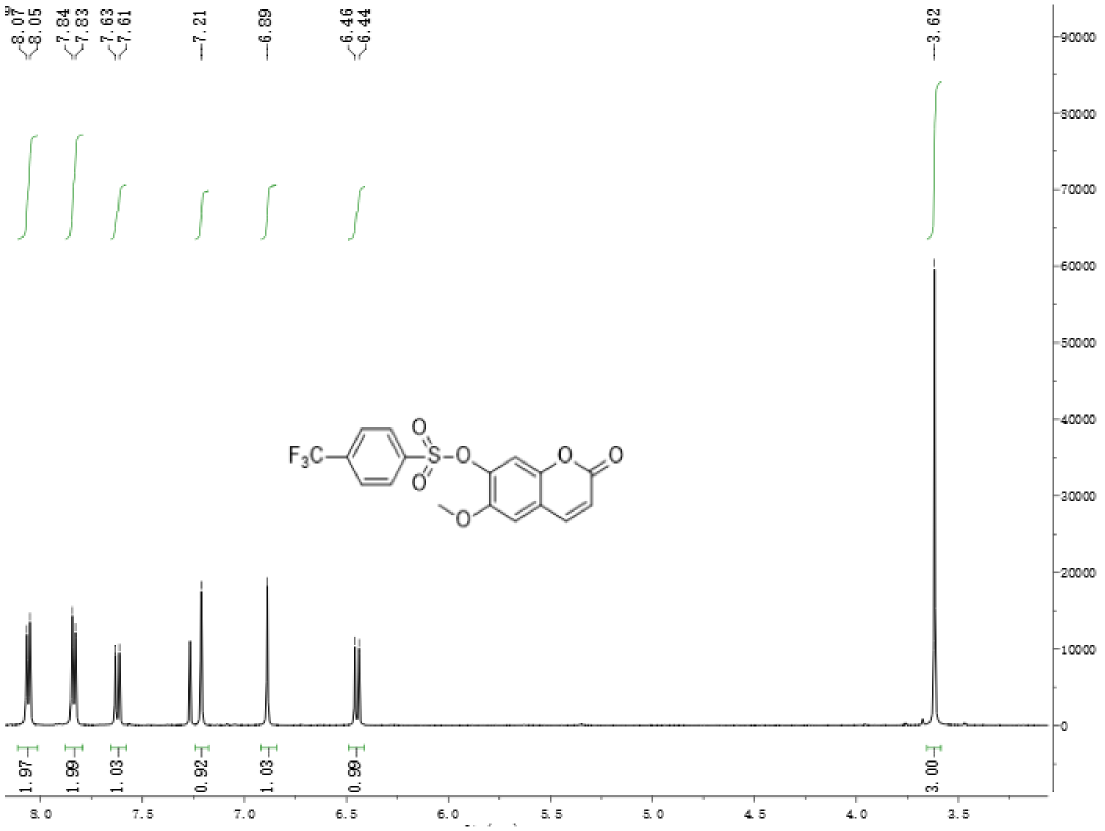

Figure S16 ¹H NMR of 8f

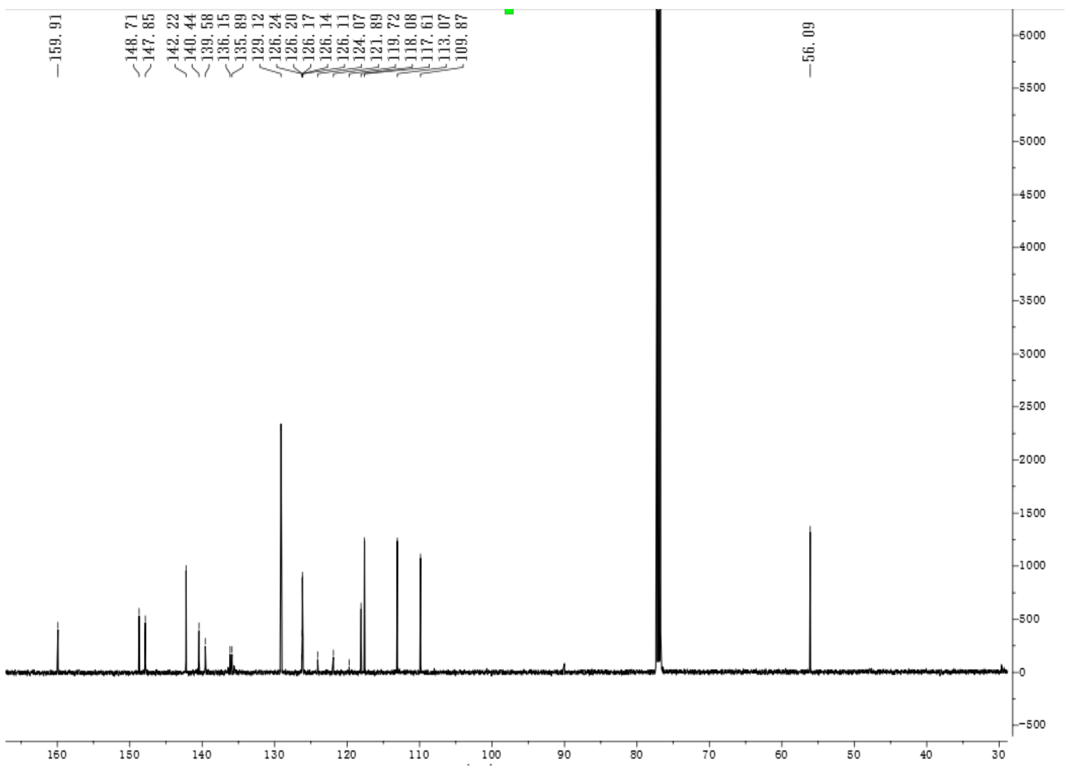

Figure S17 ^{13}C NMR of 8f

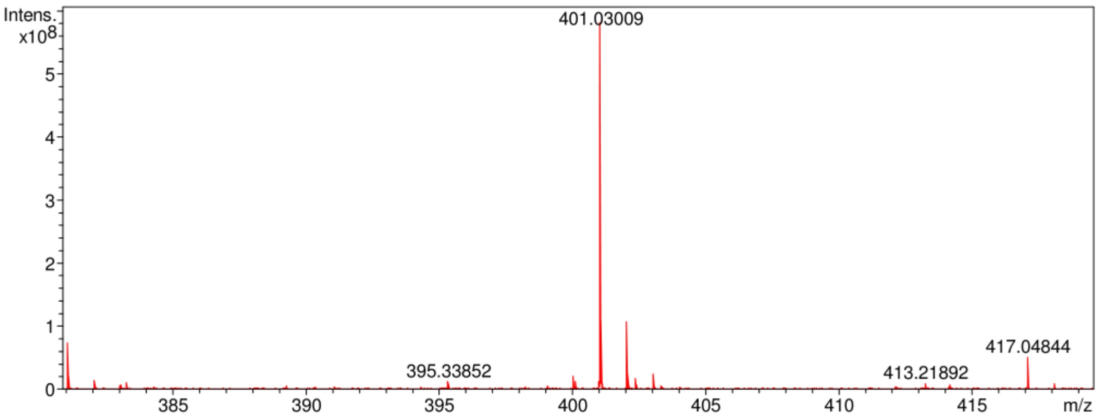

Figure S18 HRMS of 8f

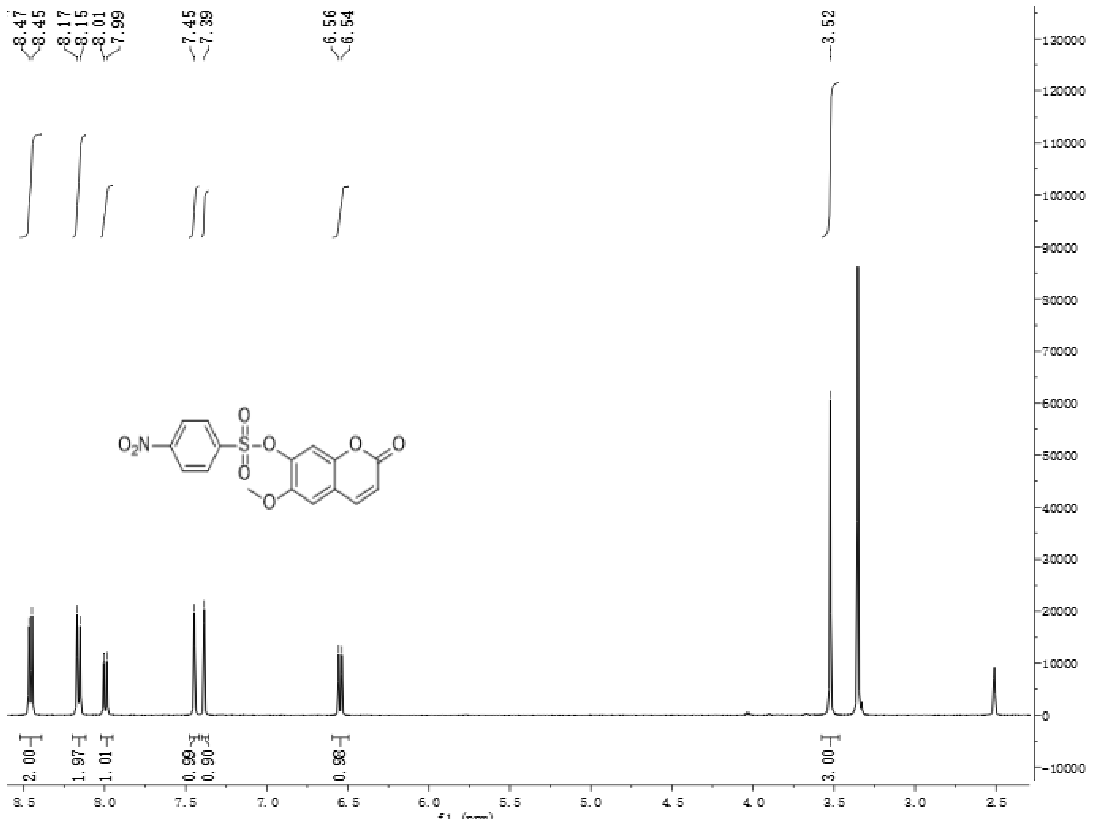

Figure S19 ^{1}H NMR of 8g

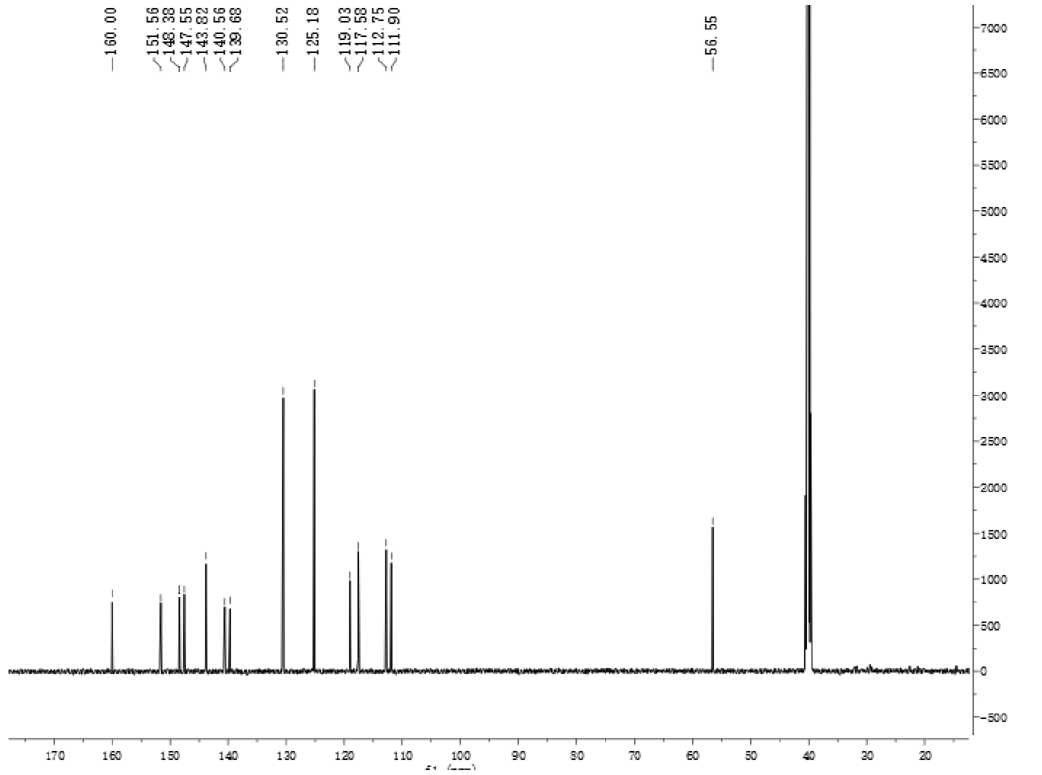

Figure S20 ^{13}C NMR of 8g

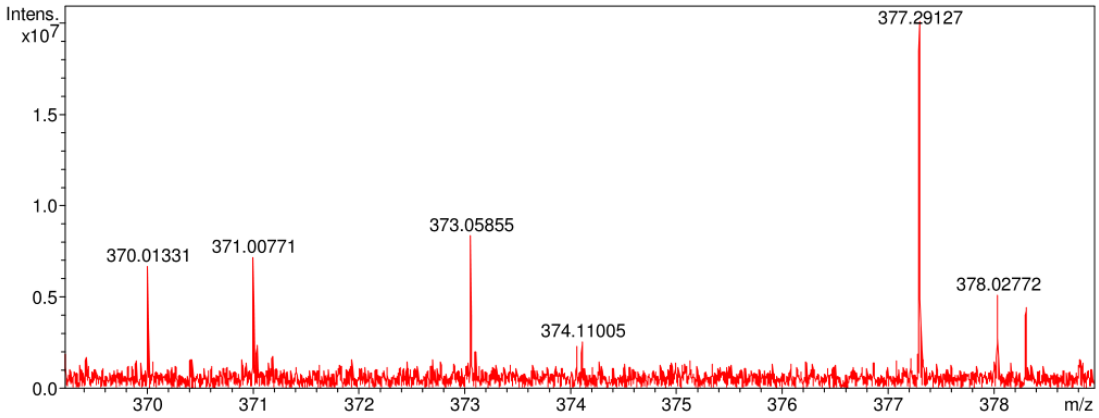

Figure S21 HRMS of 8g

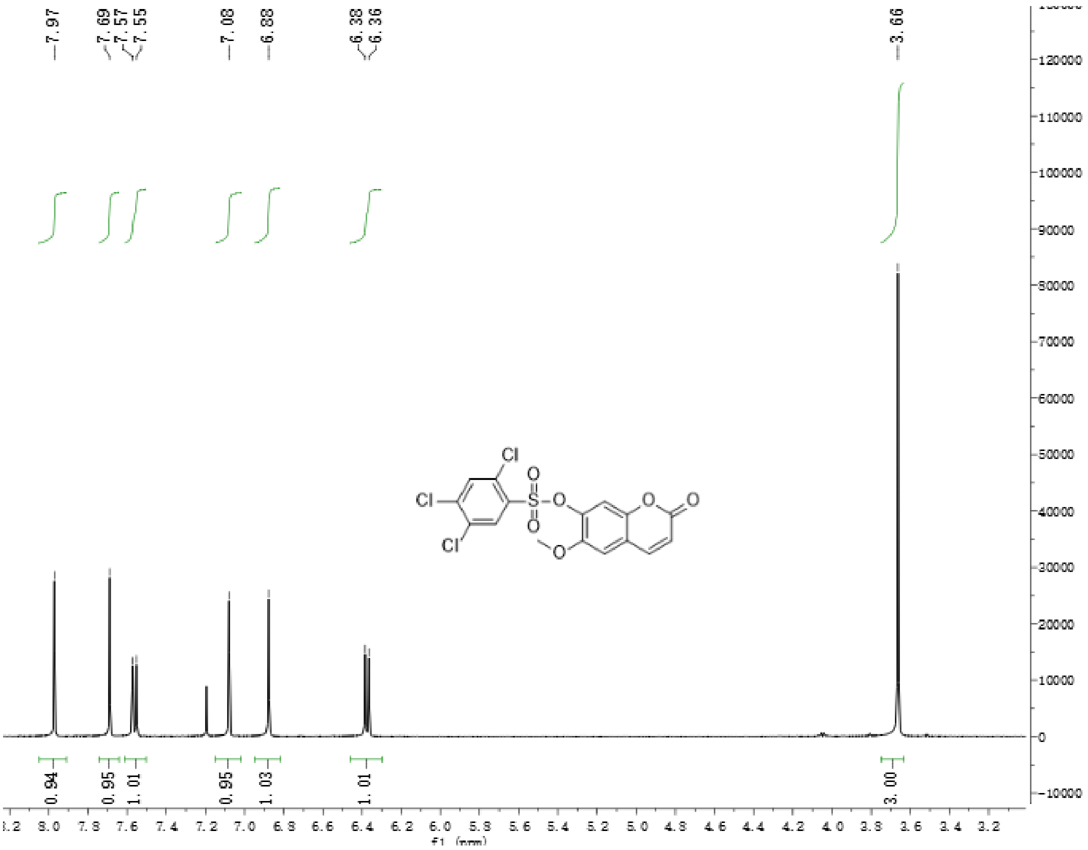

Figure S22 ^{1}H NMR of 8h

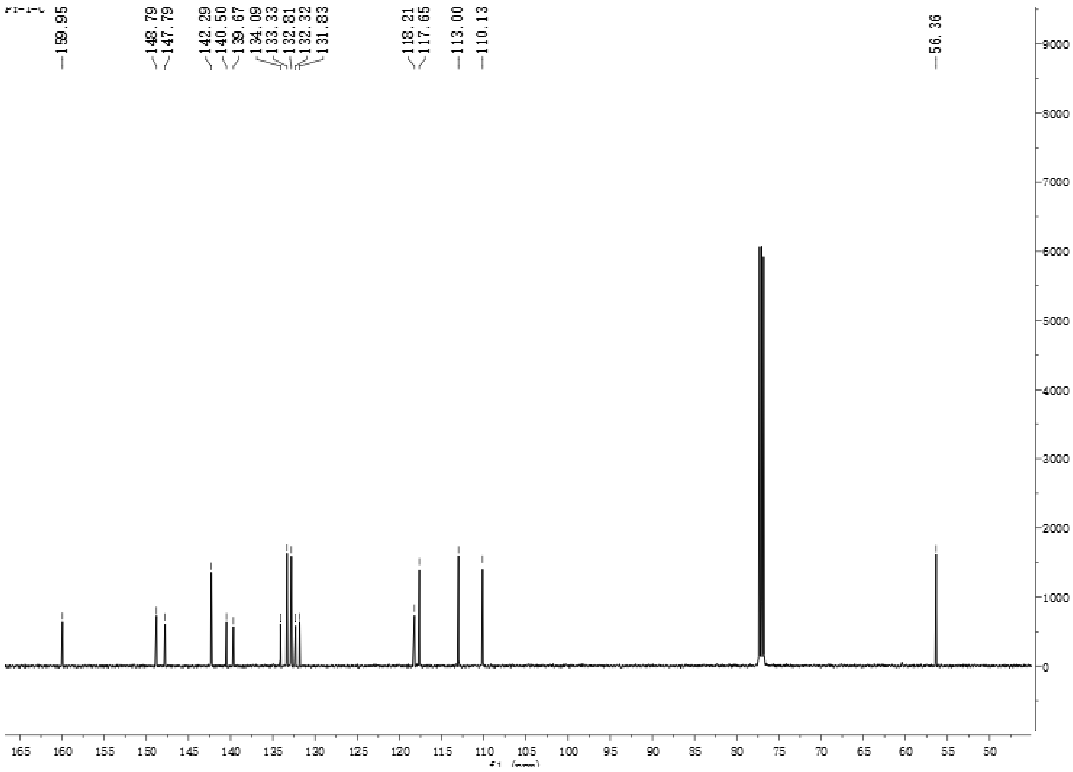

Figure S23 ^{13}C NMR of 8h

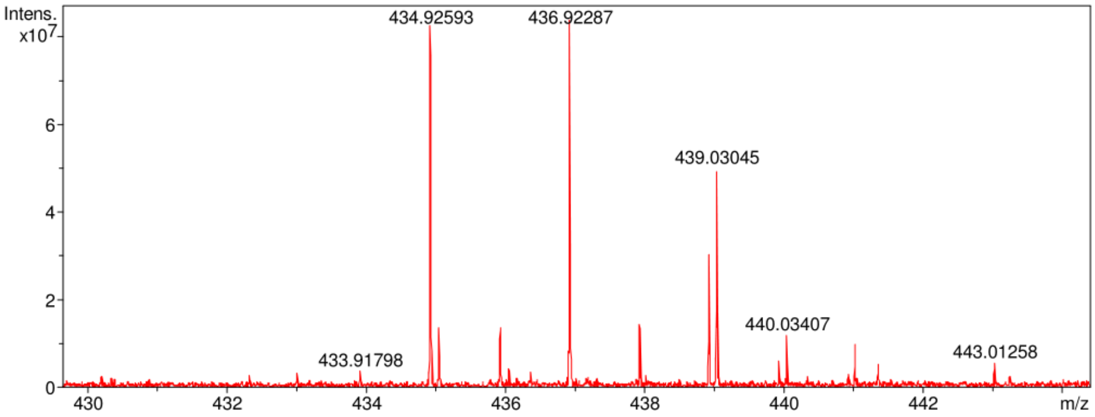

Figure S24 HRMS of 8h

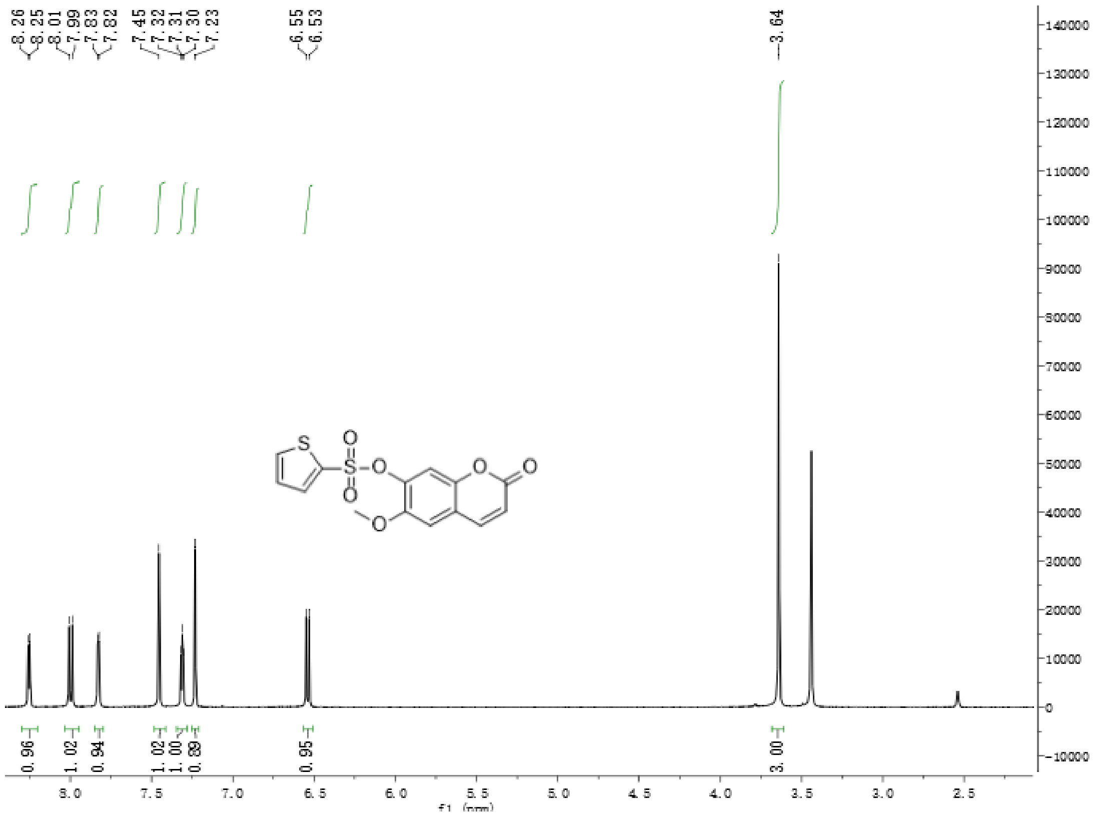

Figure S25 ^{1}H NMR of 8i

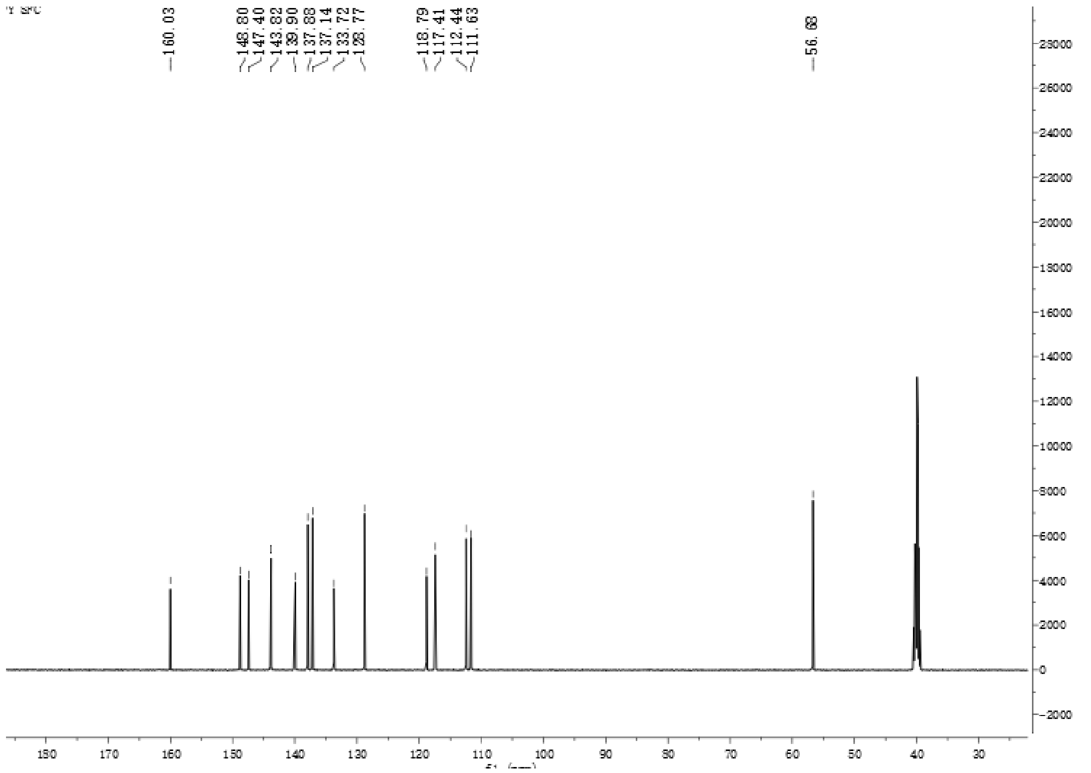

Figure S26 ^{13}C NMR of 8i

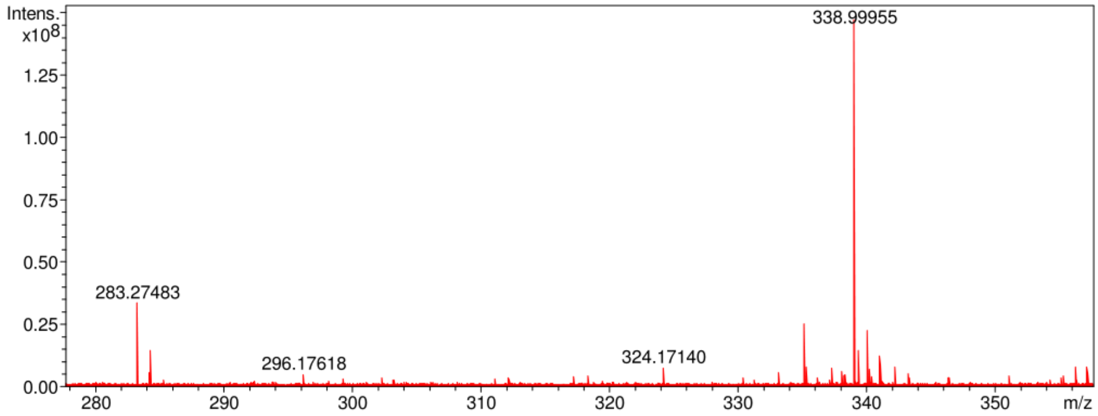

Figure S27 HRMS of 8i

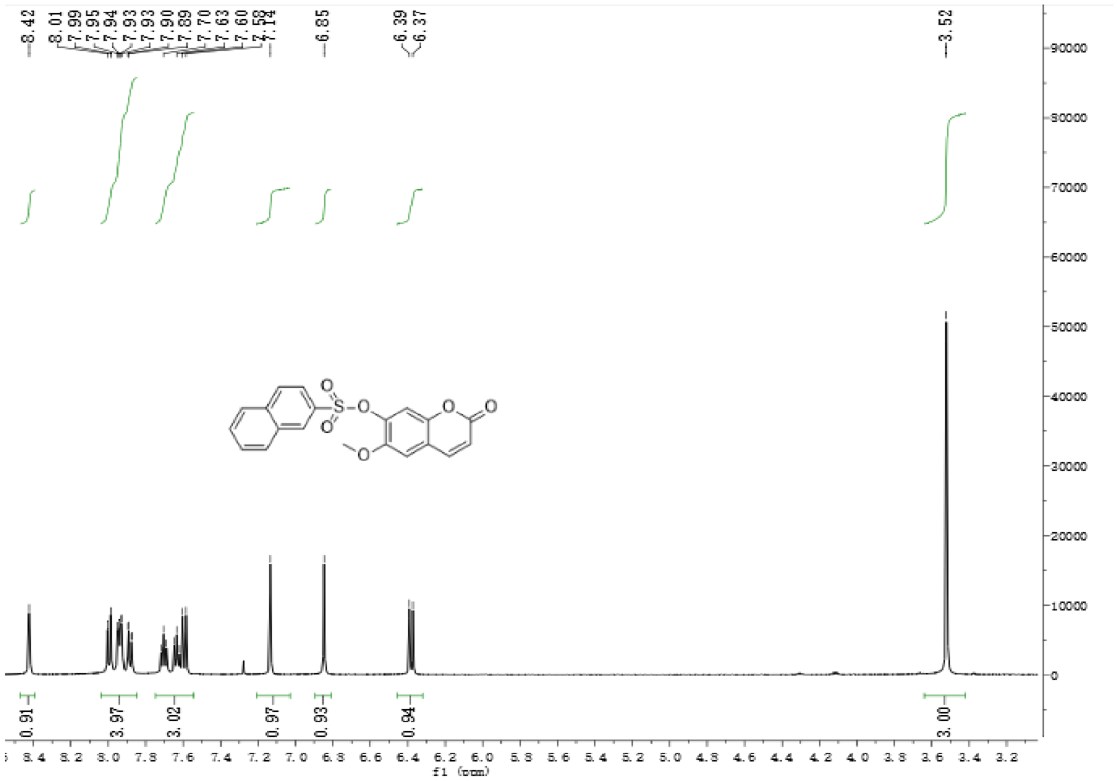

Figure S28 ^{1}H NMR of 8j

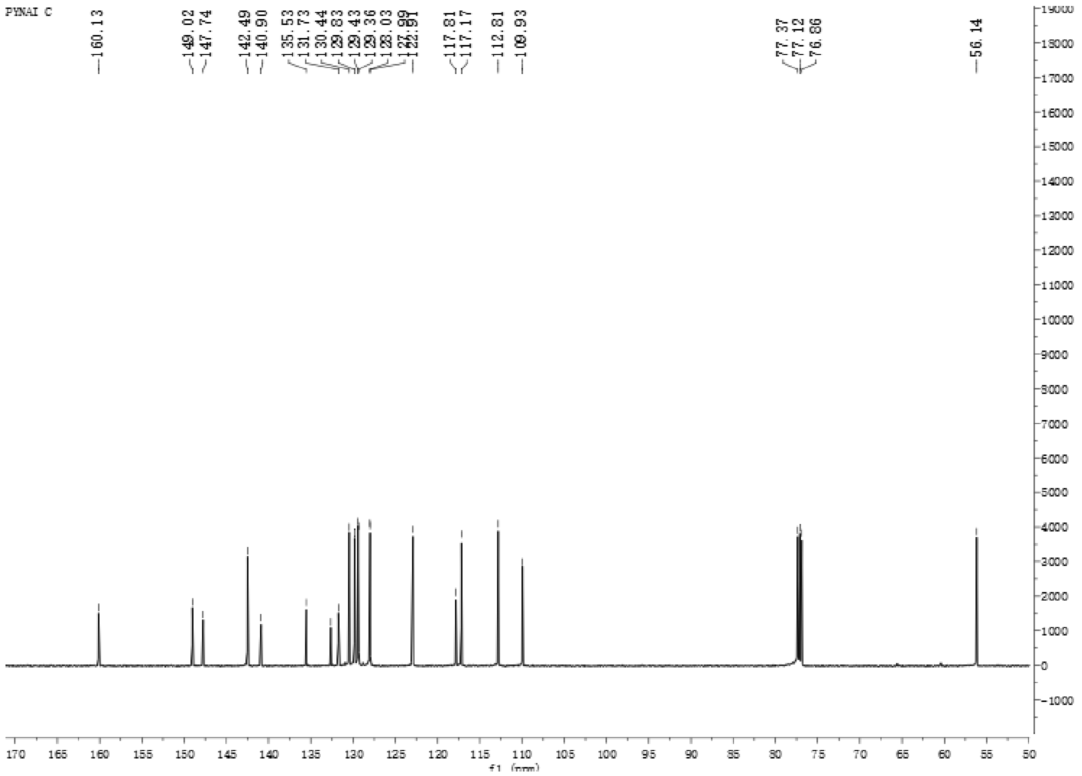

Figure S29 ^{13}**C NMR of 8j**

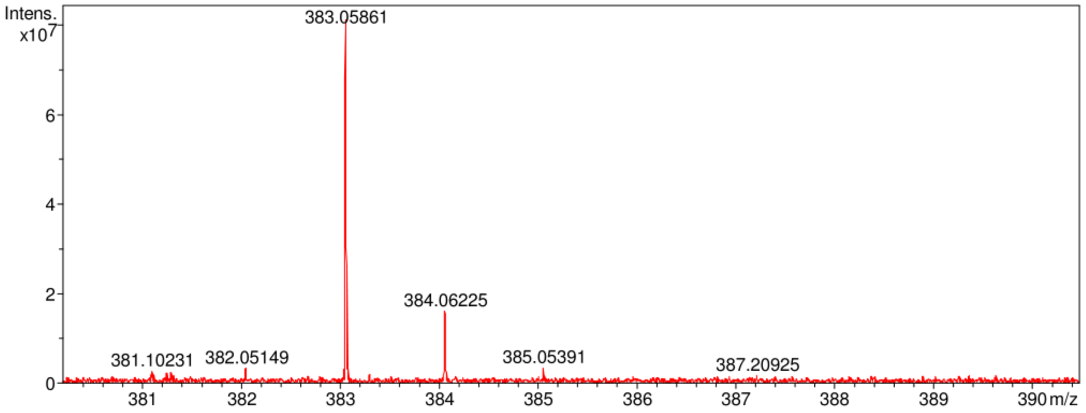

Figure S30 HRMS of 8j

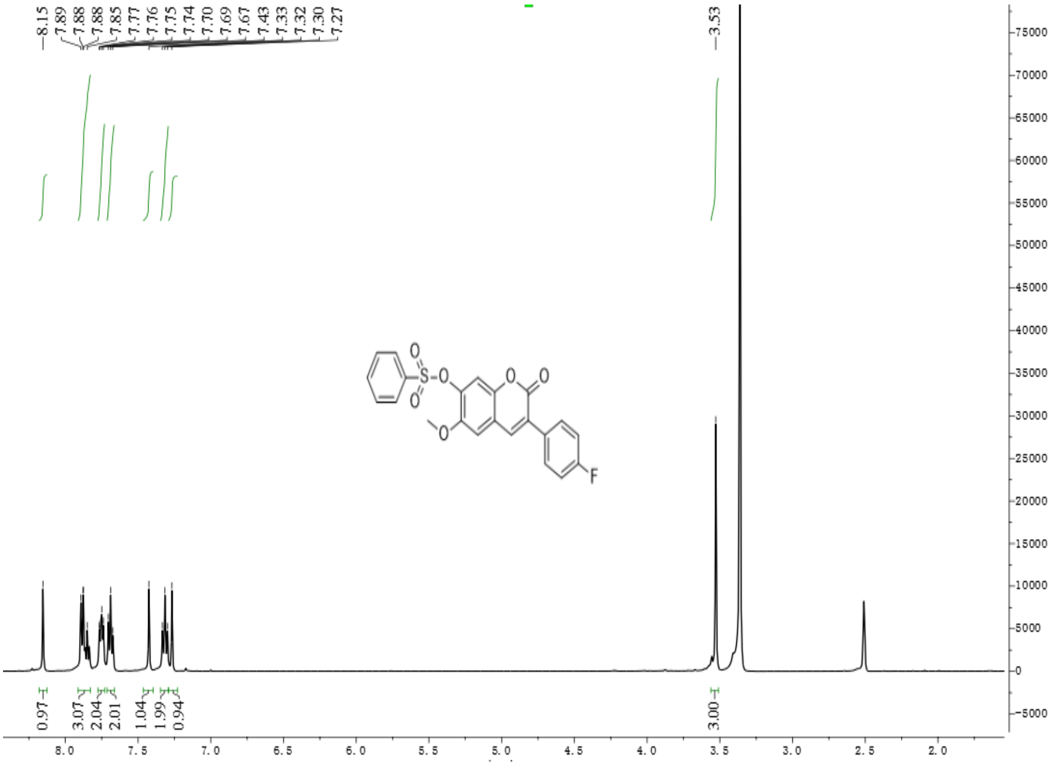

Figure S31 ^{1}H NMR of 9a

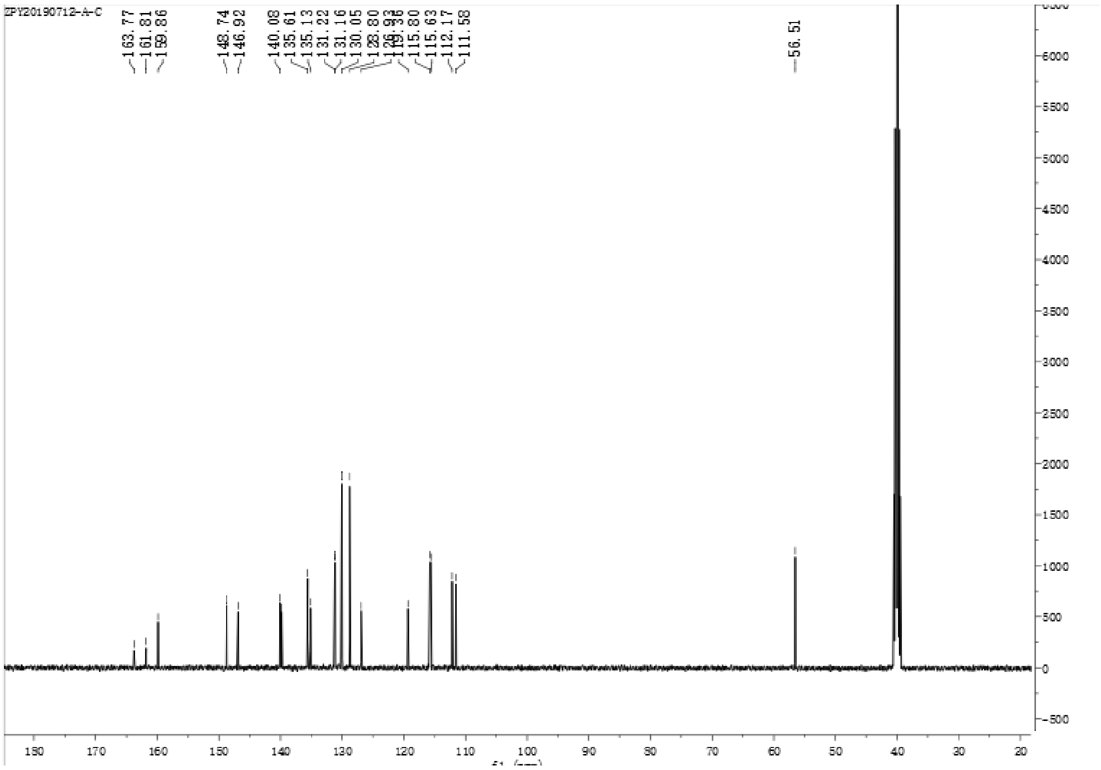

Figure S32 ^{13}C NMR of 9a

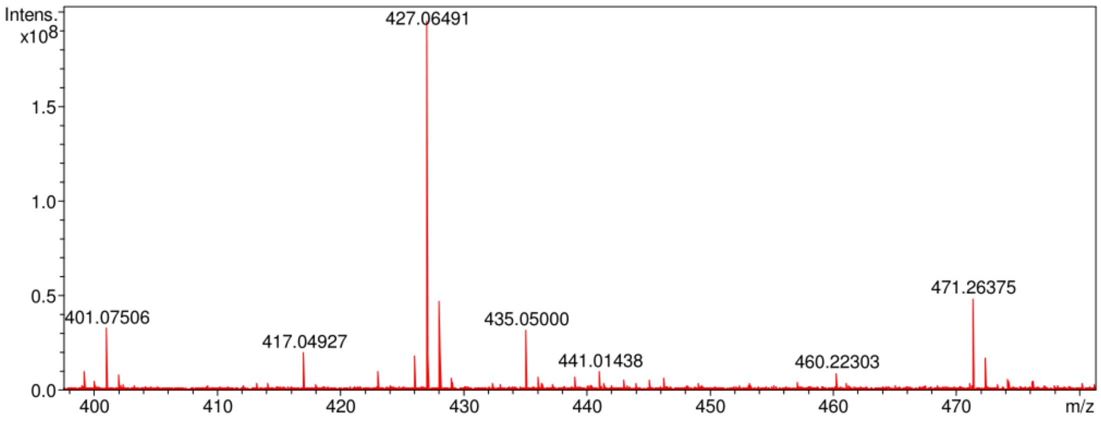

Figure S33 HRMS of 9a

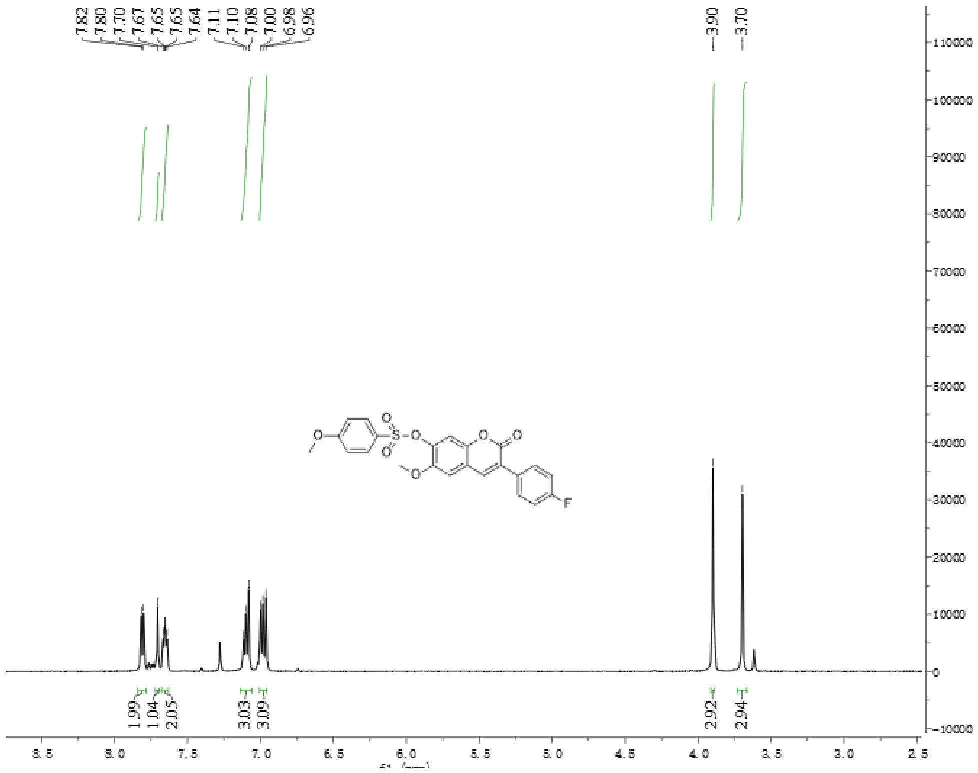

Figure S34 ^{1}H NMR of 9b

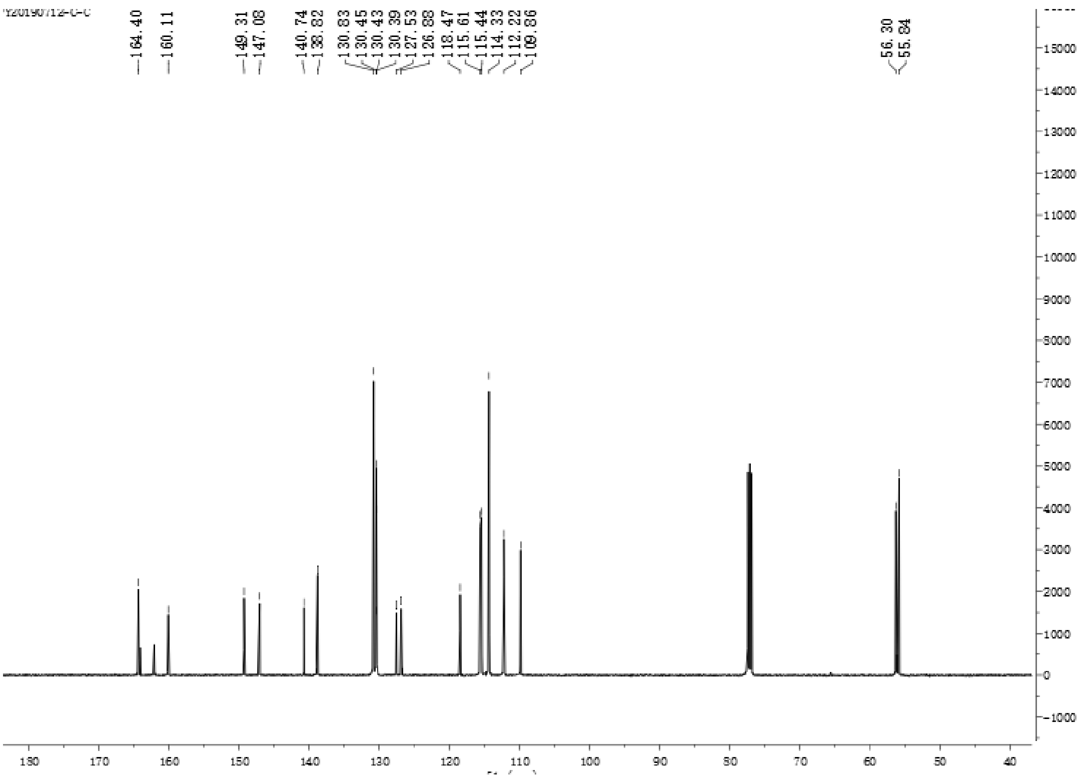

Figure S35 ^{13}C NMR of 9b

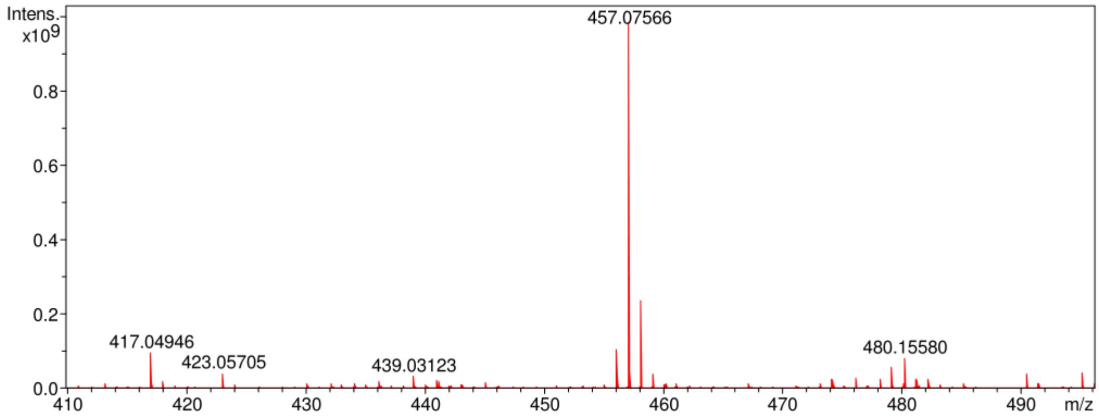

Figure S36 HRMS of 9b

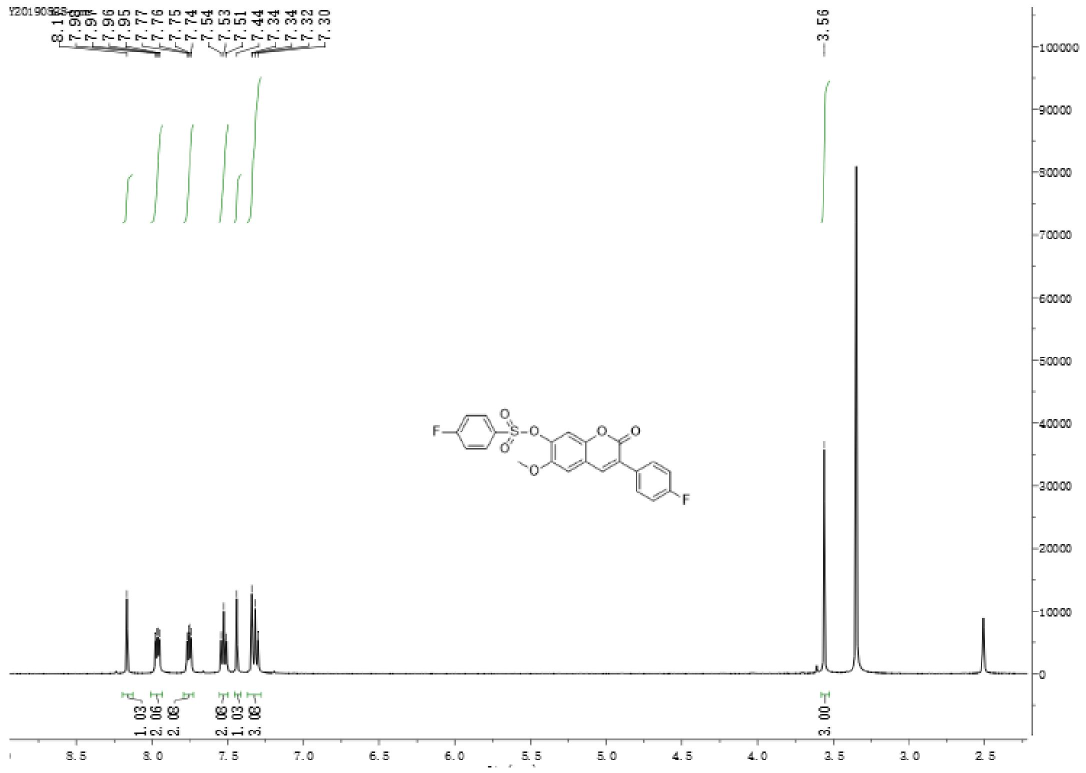

Figure S37 ^{1}H NMR of 9c

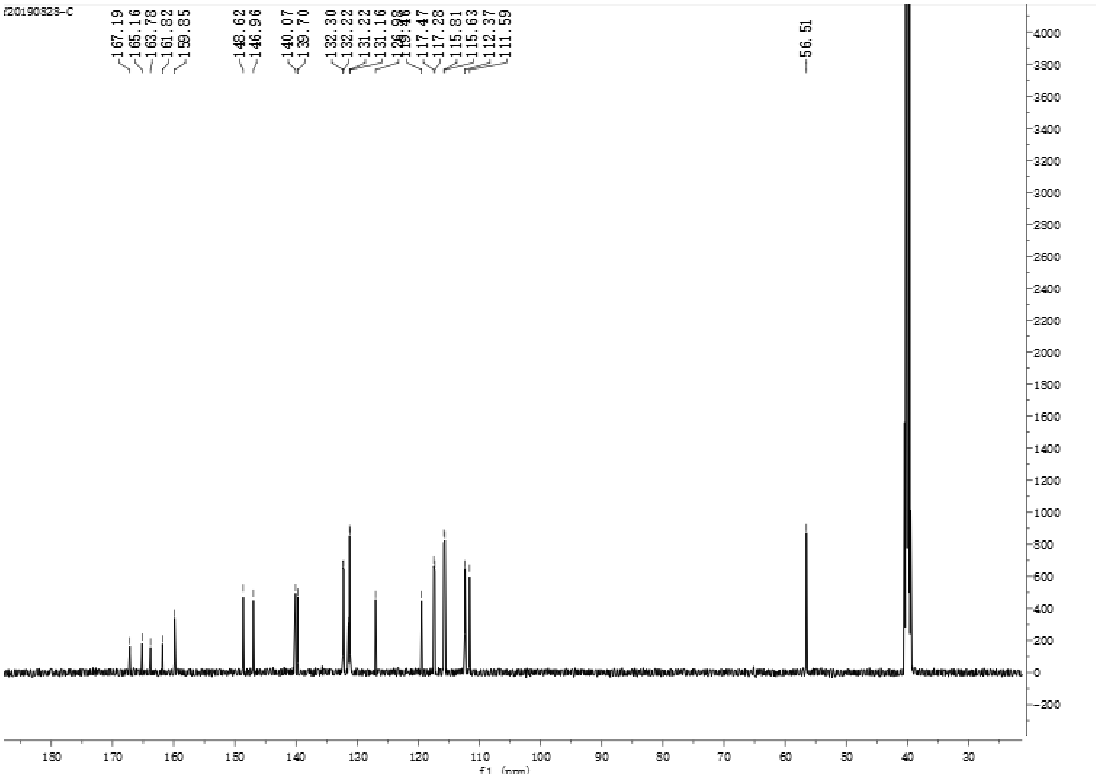

Figure S38 ^{13}C NMR of 9c

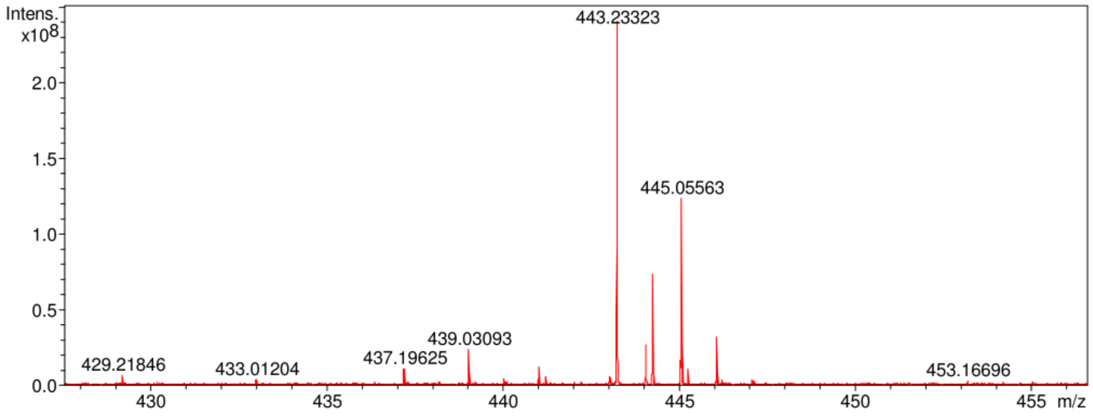

Figure S39 HRMS of 9c

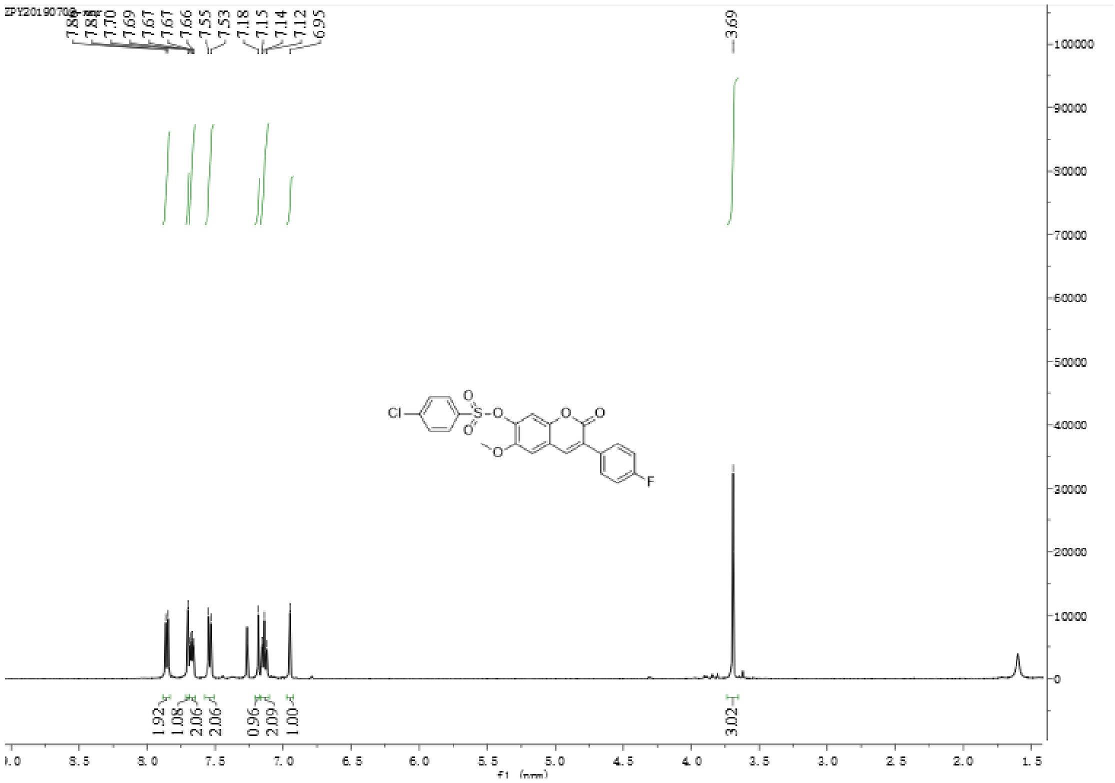

Figure S40 ¹H NMR of 9d

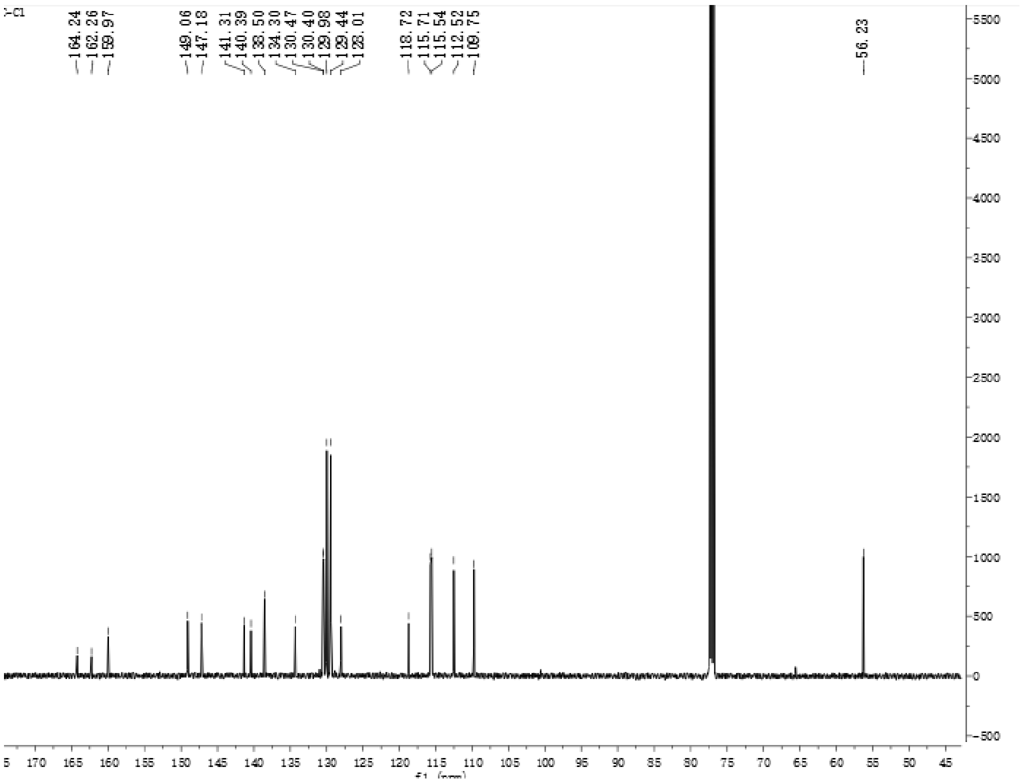

Figure S41 ^{13}C NMR of 9d

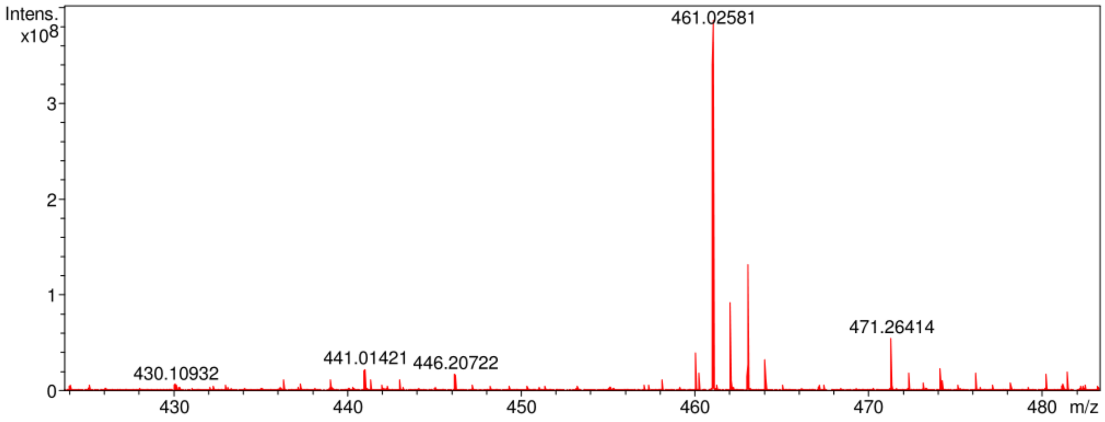

Figure S42 HRMS of 9d

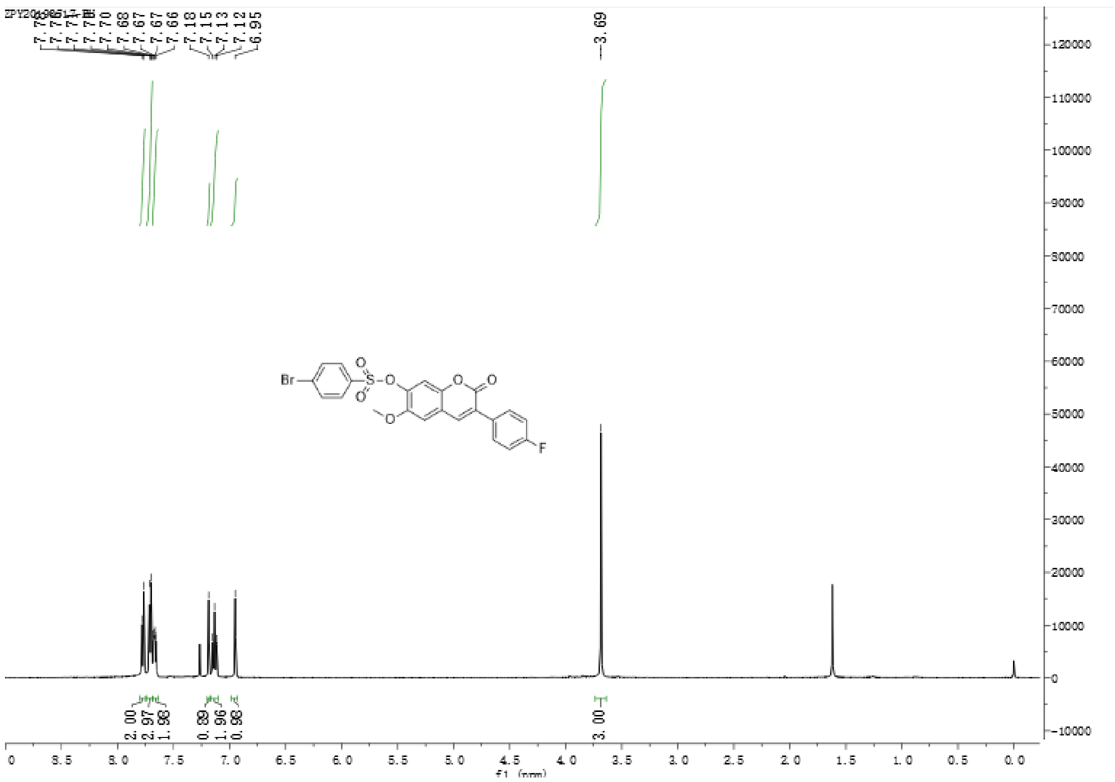

Figure S43 ^{1}H NMR of 9e

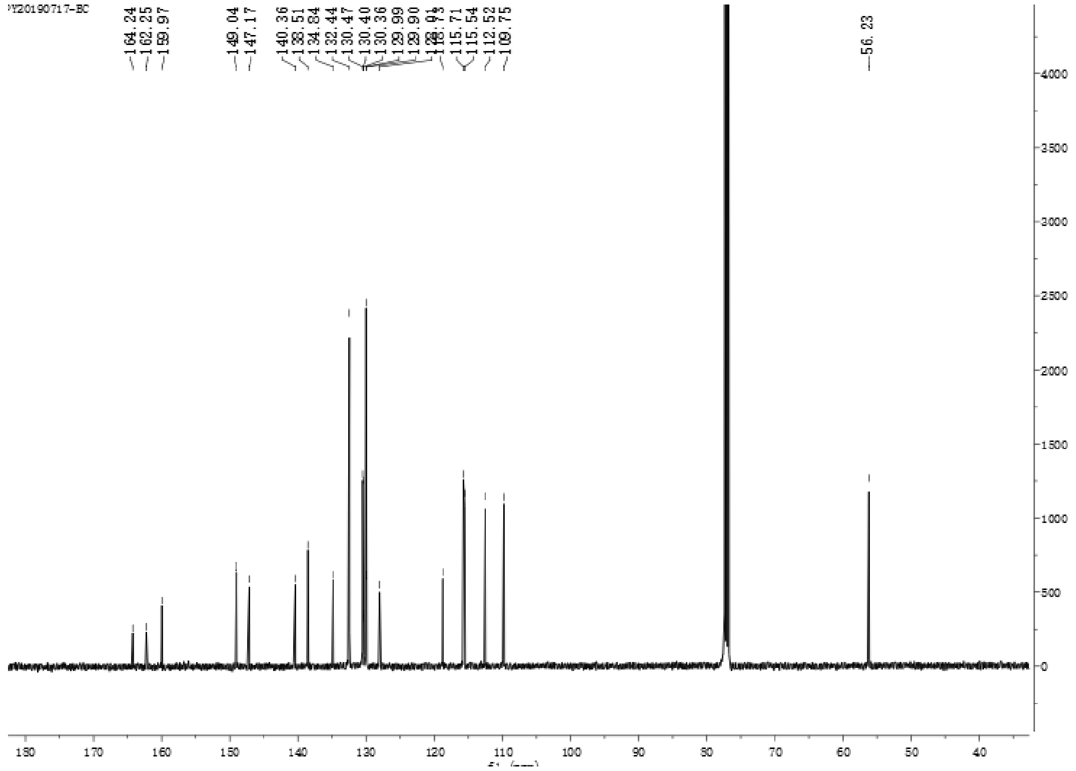

Figure S44 ^{13}C NMR of 9e

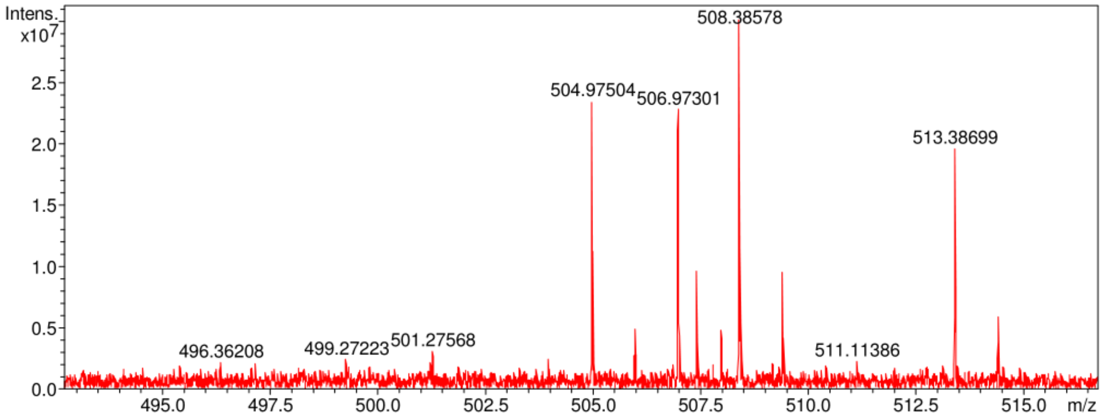

Figure S45 HRMS of 9e

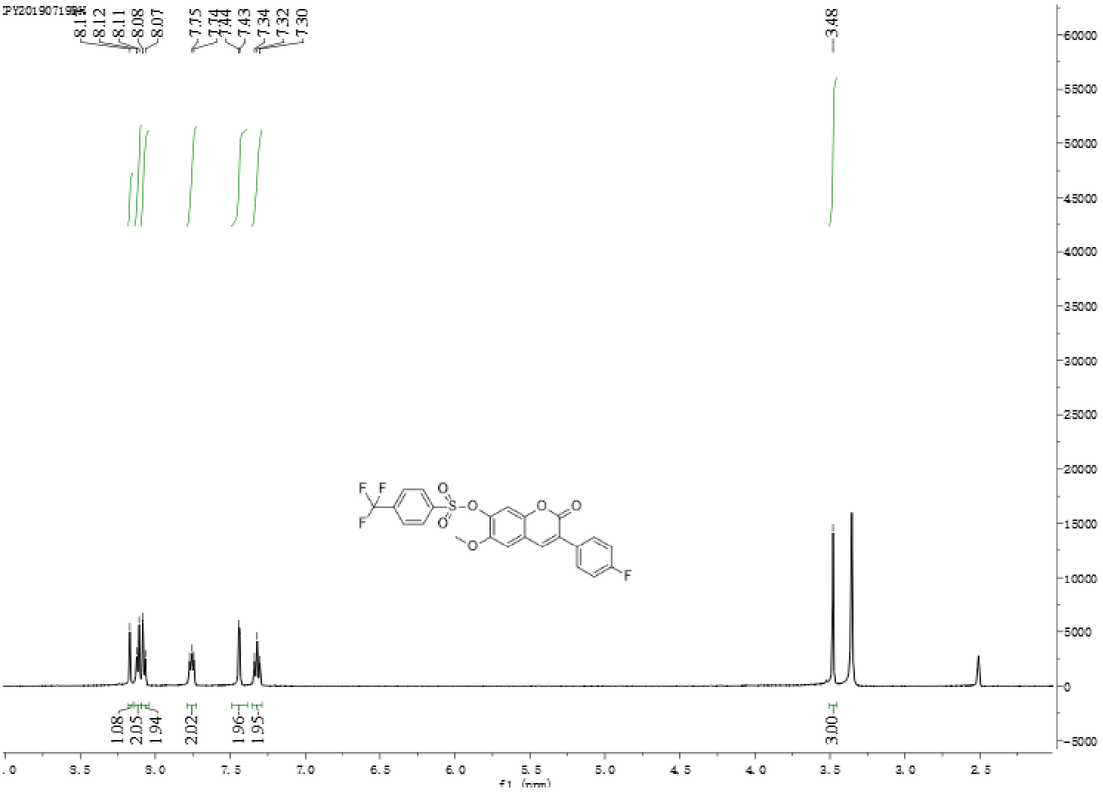

Figure S46 ^{1}H NMR of 9f

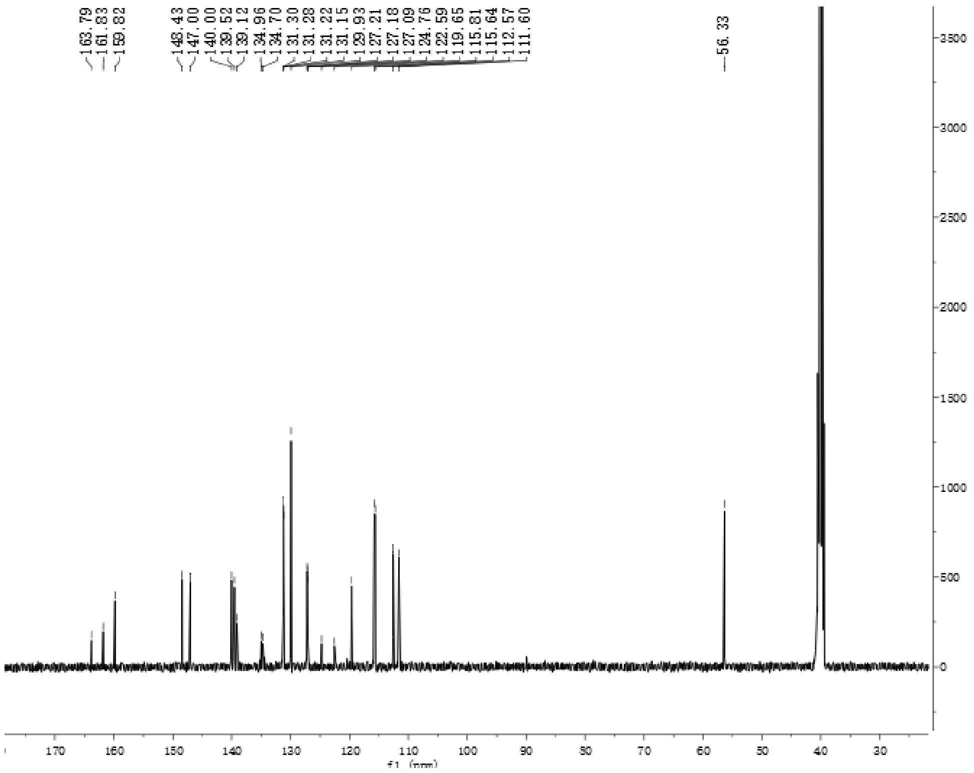

Figure S47 ^{13}C NMR of 9f

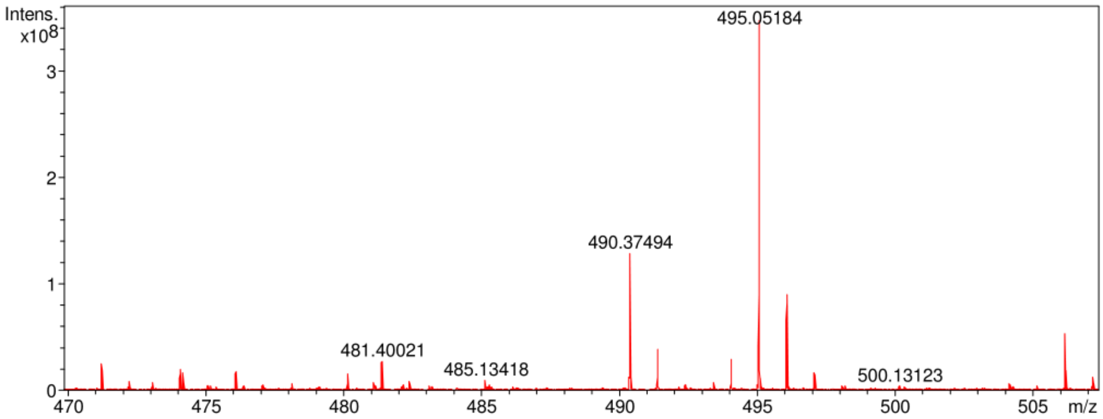

Figure S48 HRMS of 9f

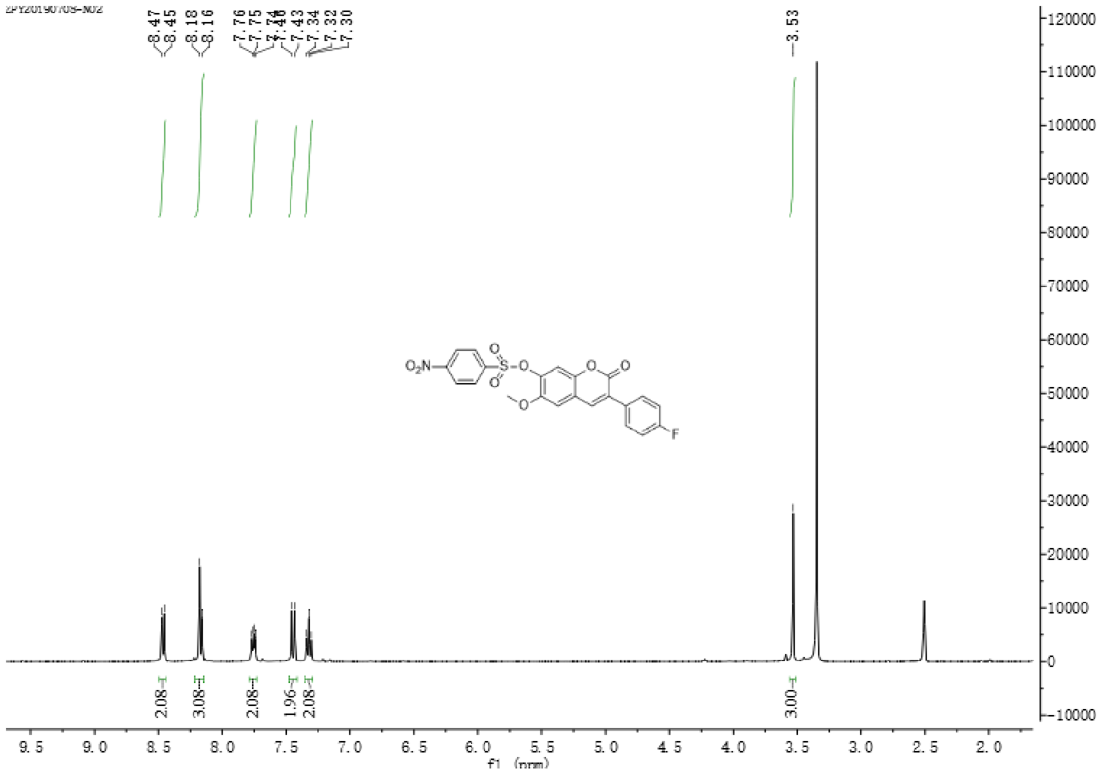

Figure S49 ^{1}H NMR of 9g

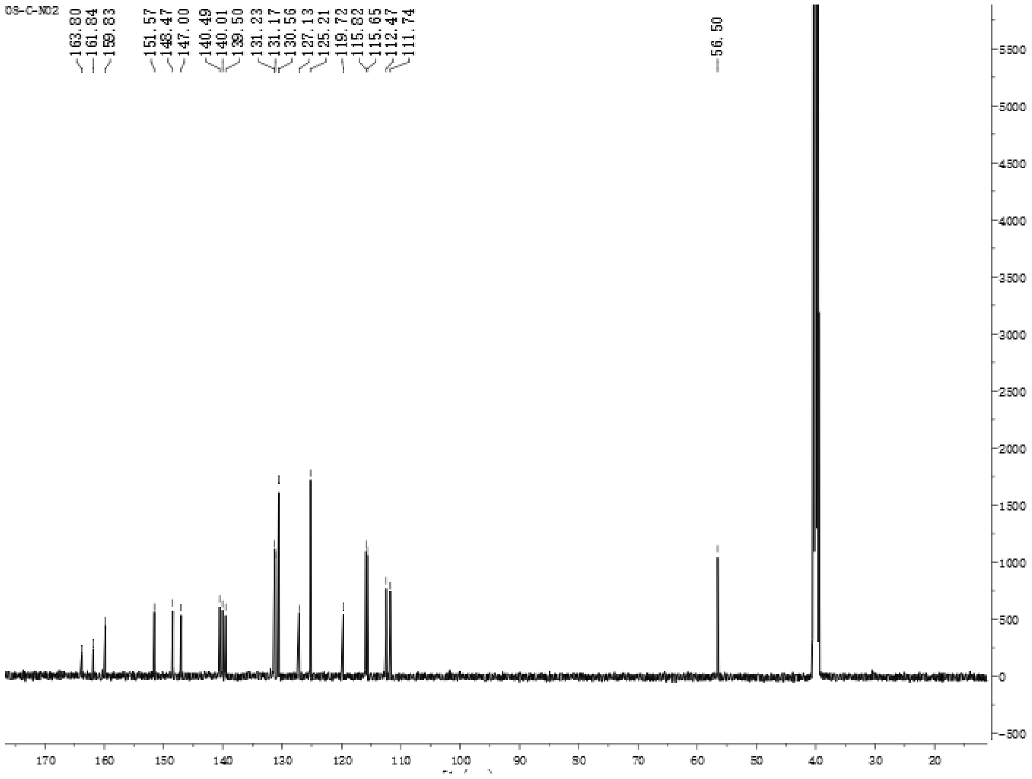

Figure S50 ^{13}C NMR of 9g

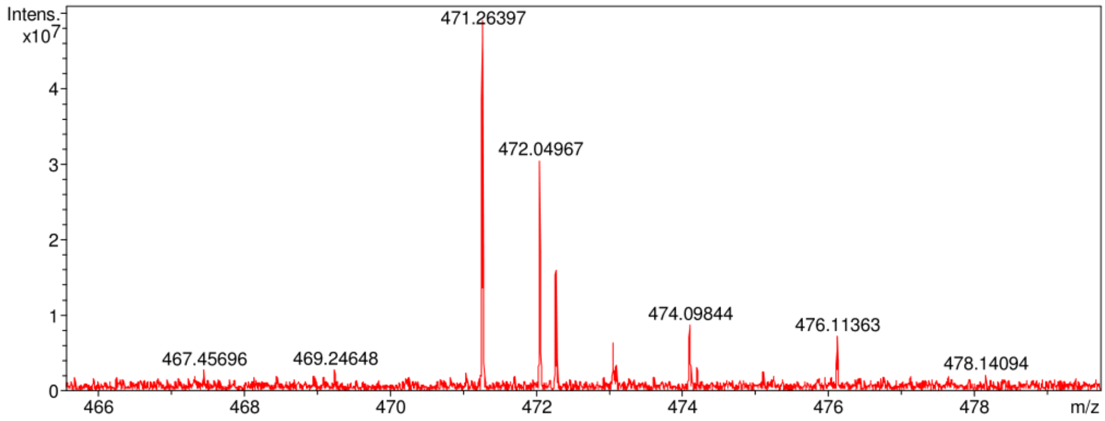

Figure S51 HRMS of 9g

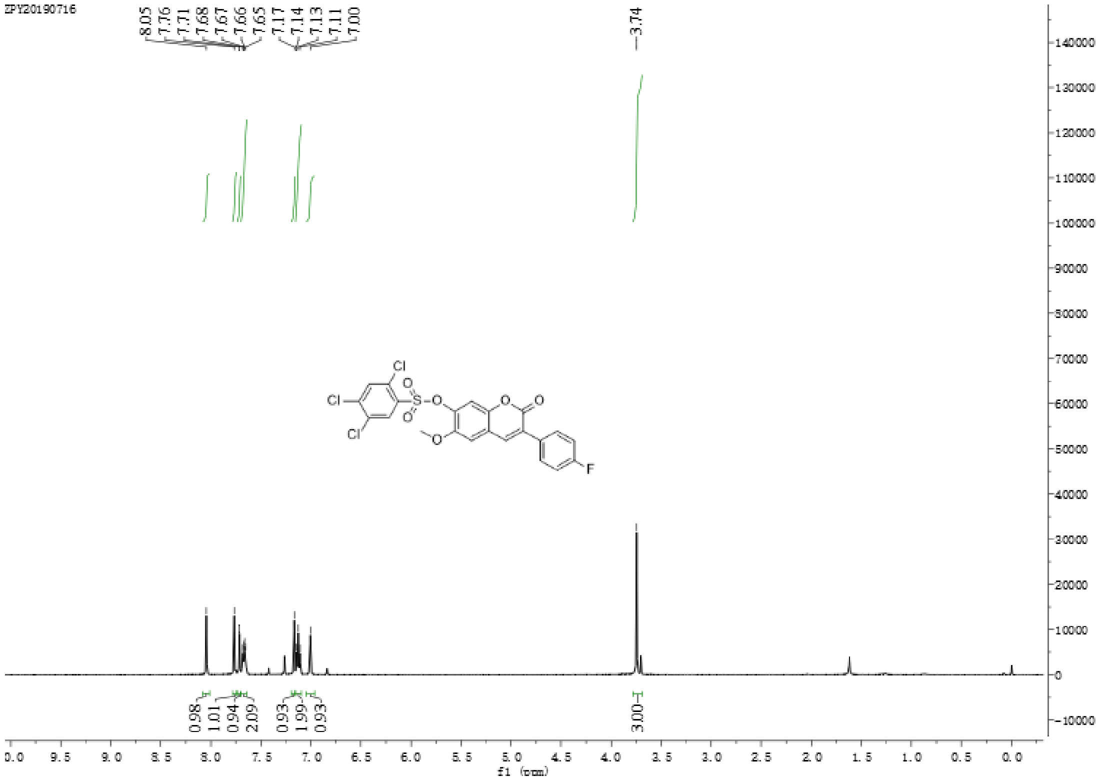

Figure S52 ^{1}H NMR of 9h

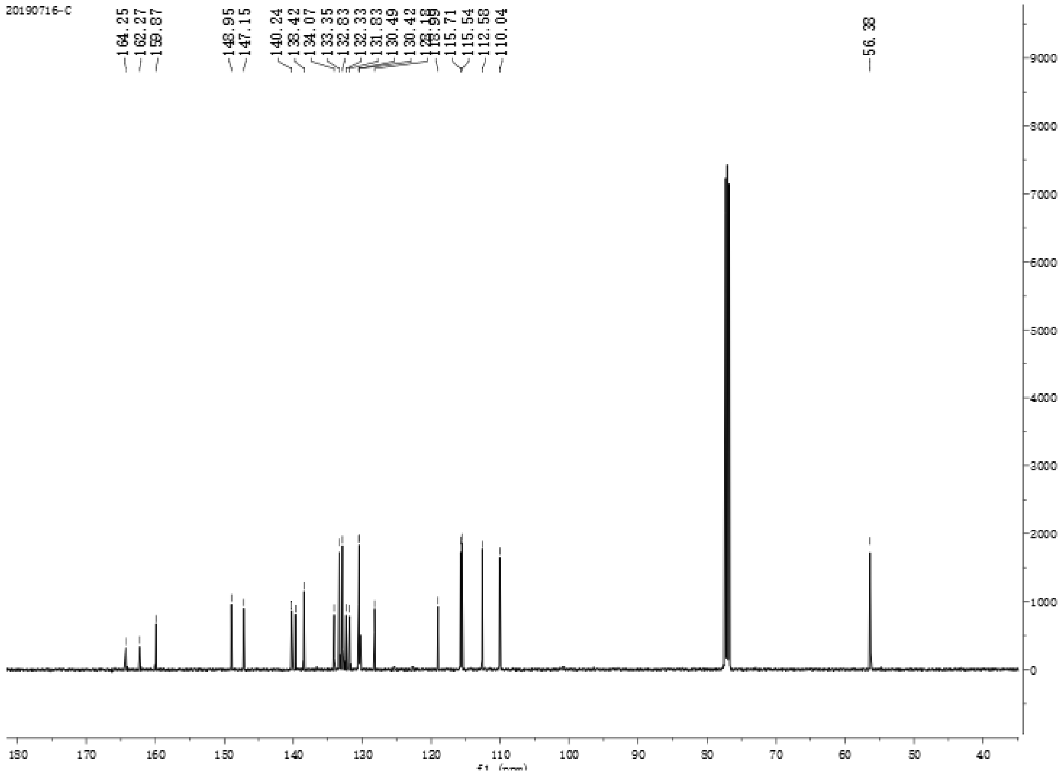

Figure S53 ^{13}C NMR of 9h

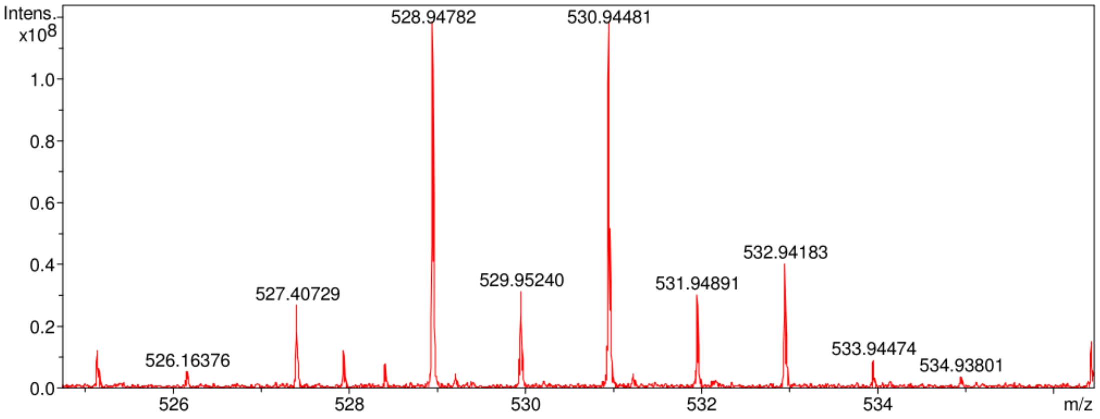

Figure S54 HRMS of 9h

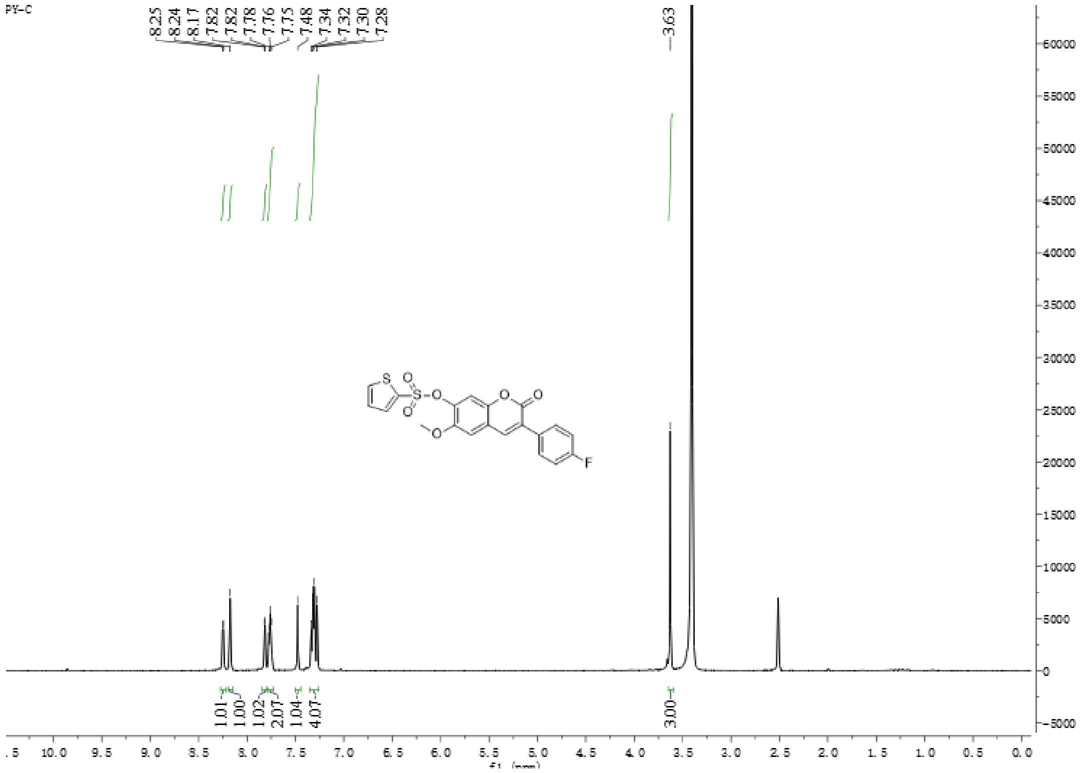

Figure S55 ^{1}H NMR of 9i

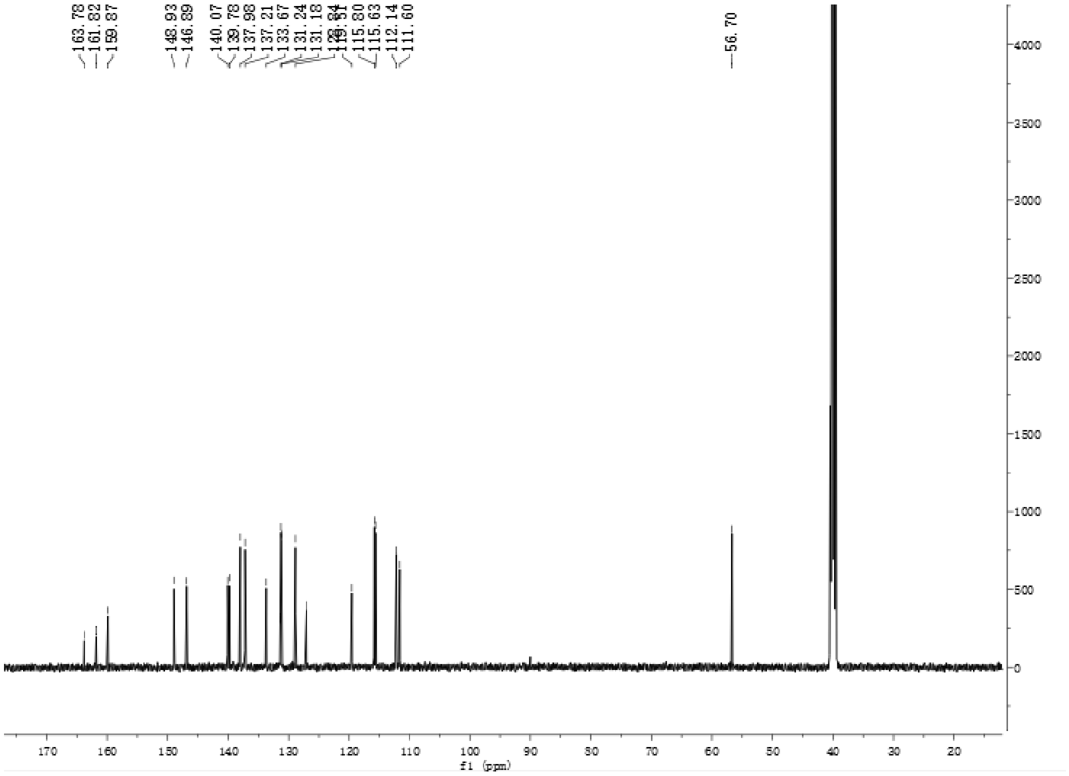

Figure S56 ^{13}C NMR of 9i

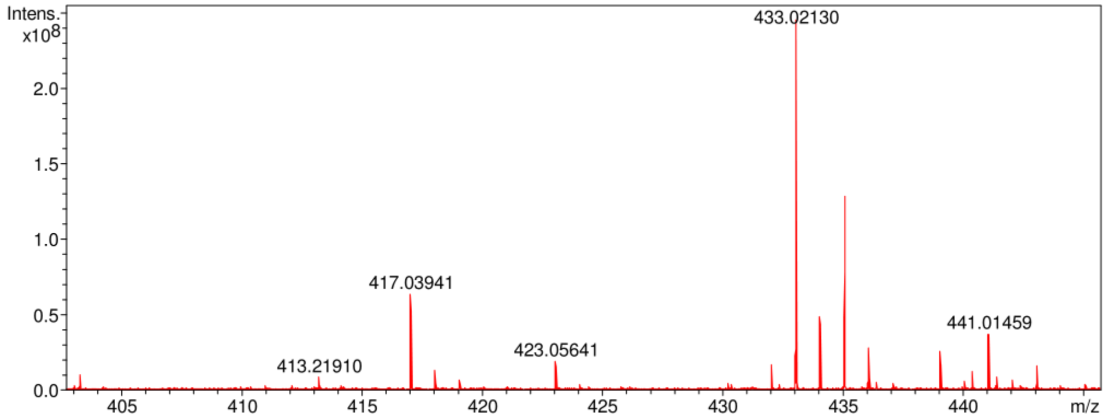

Figure S57 HRMS of 9i

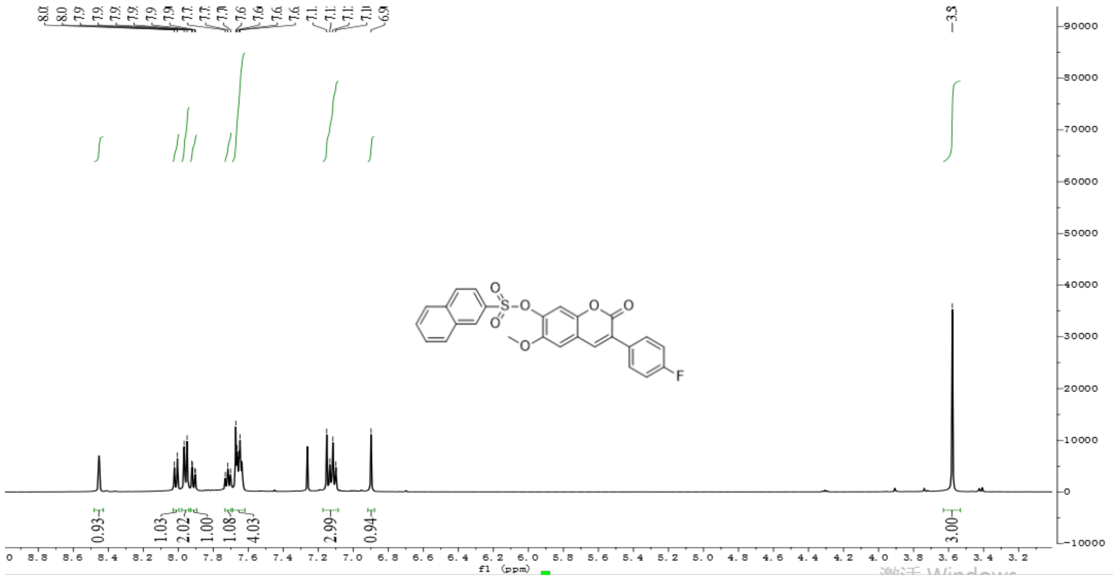

Figure S58 ^{1}H NMR of 9j

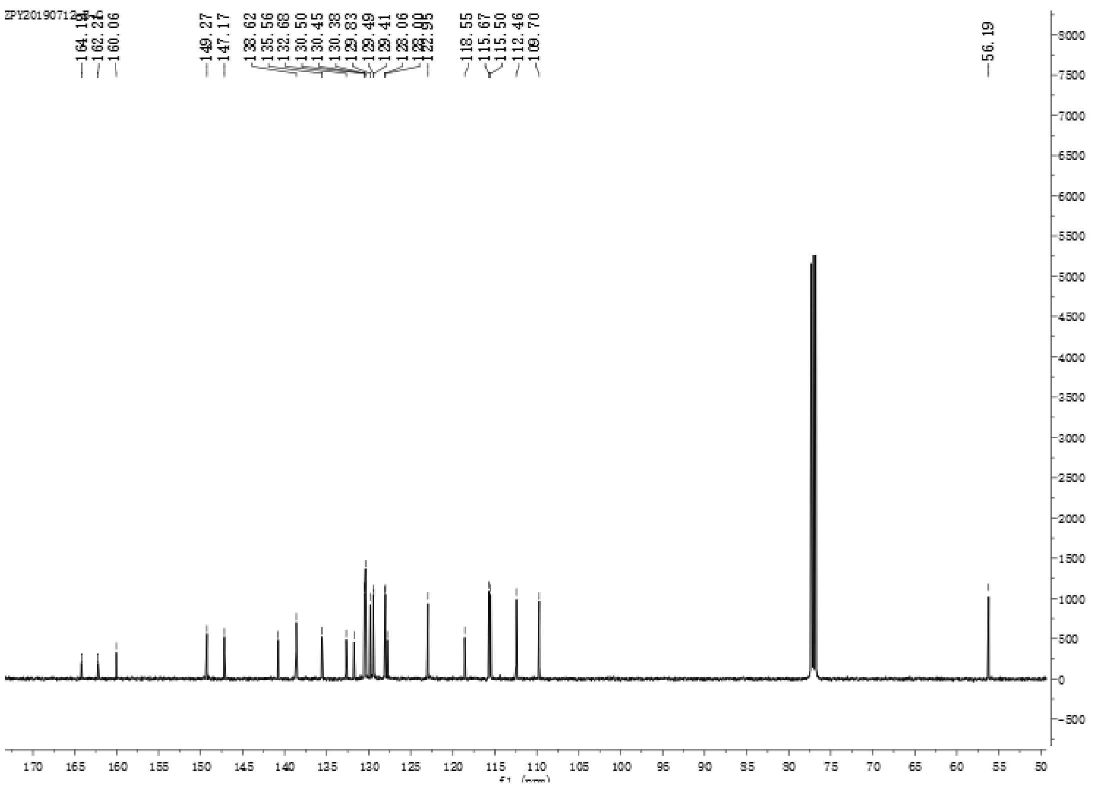

Figure S59 ^{13}C NMR of 9j

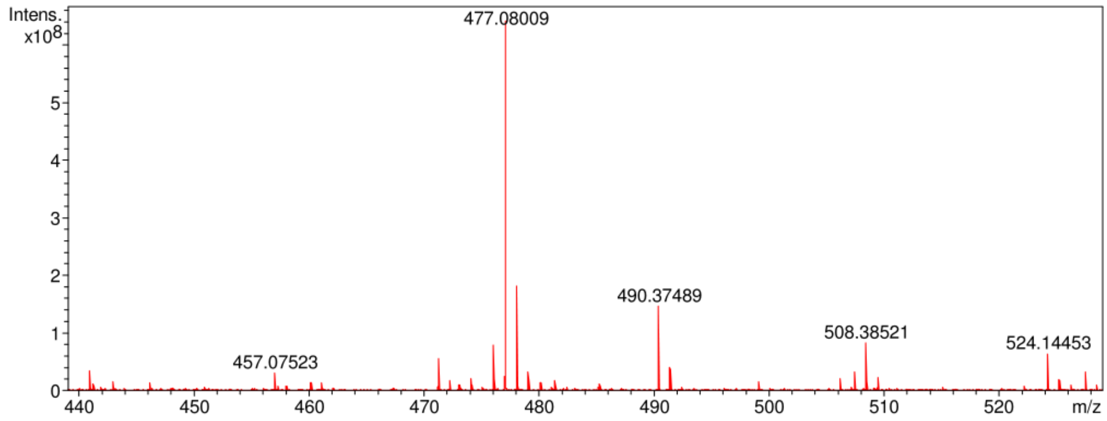

Figure S60 HRMS of 9j

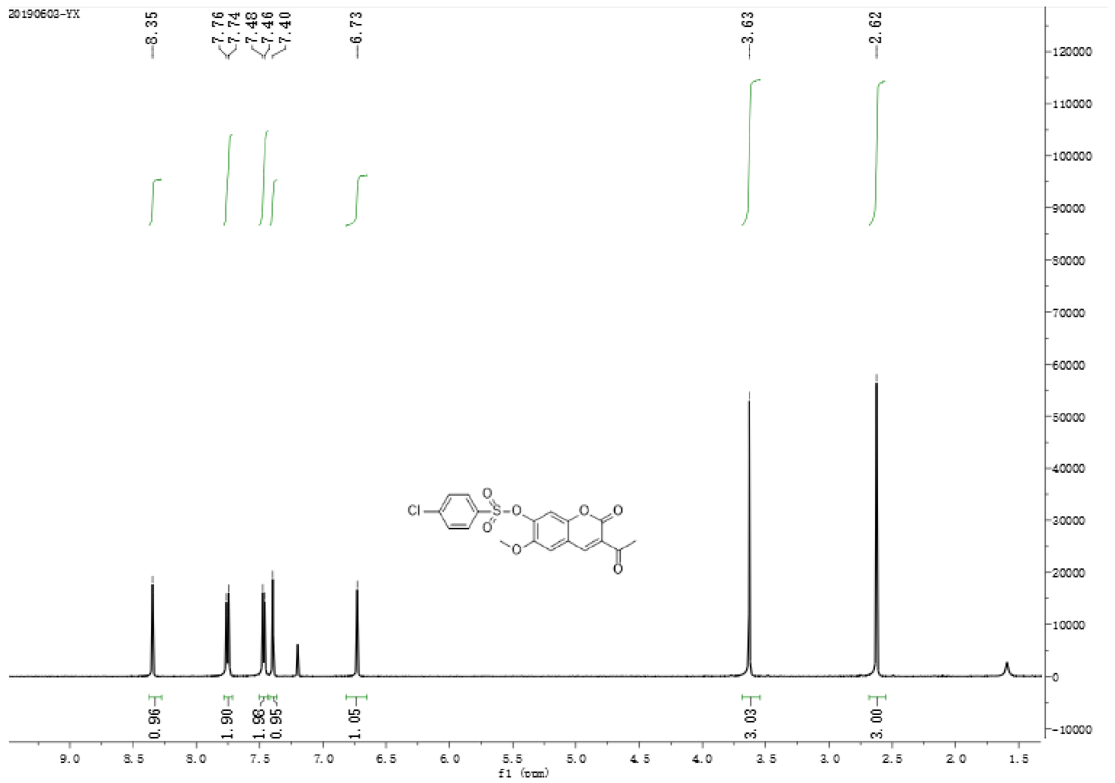

Figure S61 ^{1}H NMR of 10d

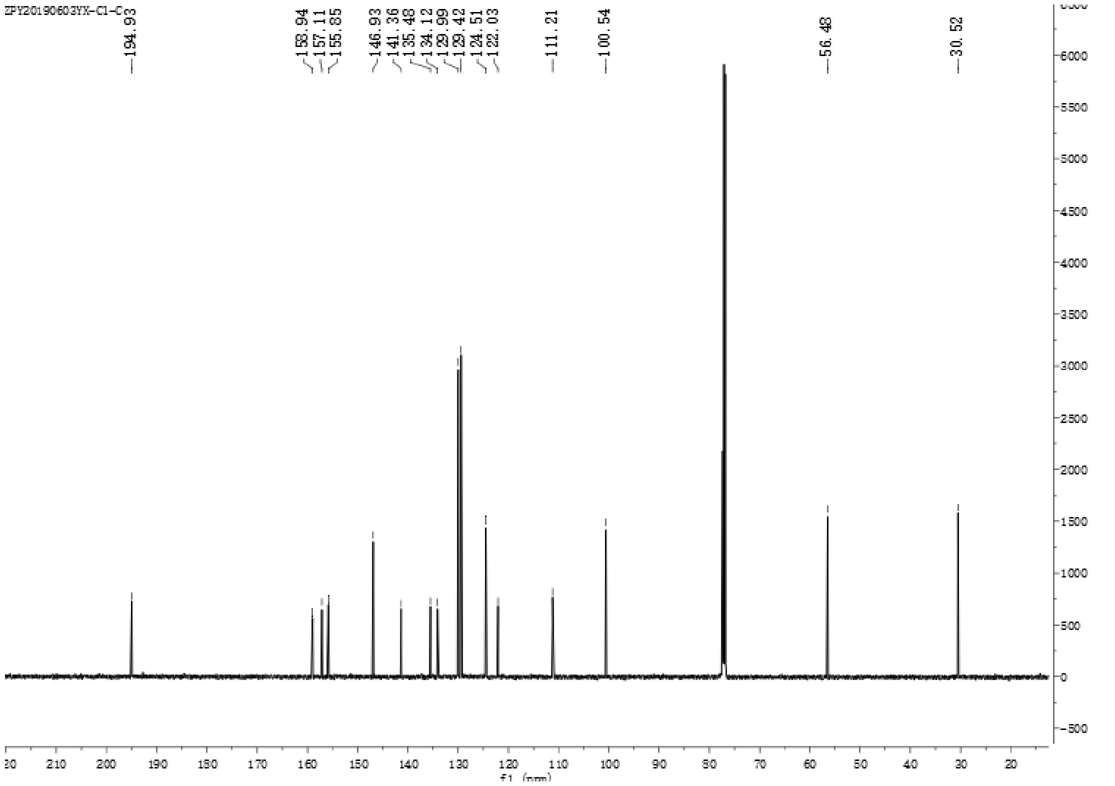

Figure S62 ^{13}C NMR of 10d

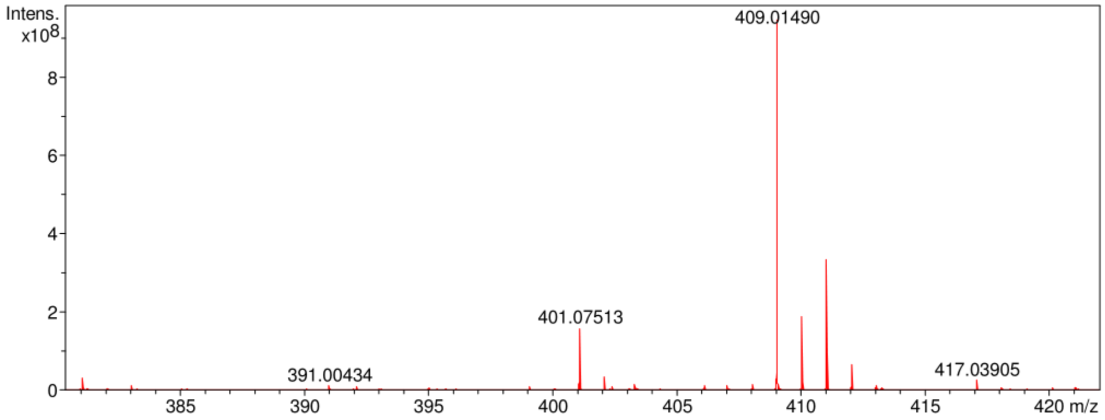

Figure S63 HRMS of 10d

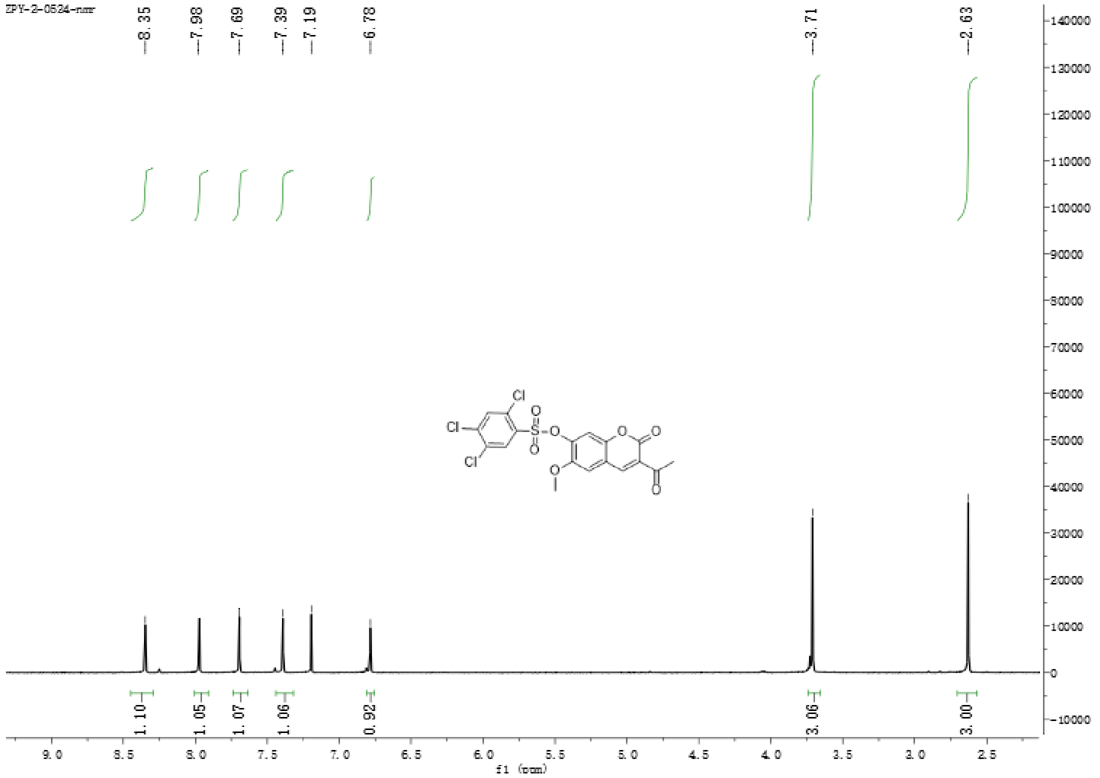

Figure S64 ^{1}H NMR of 10h

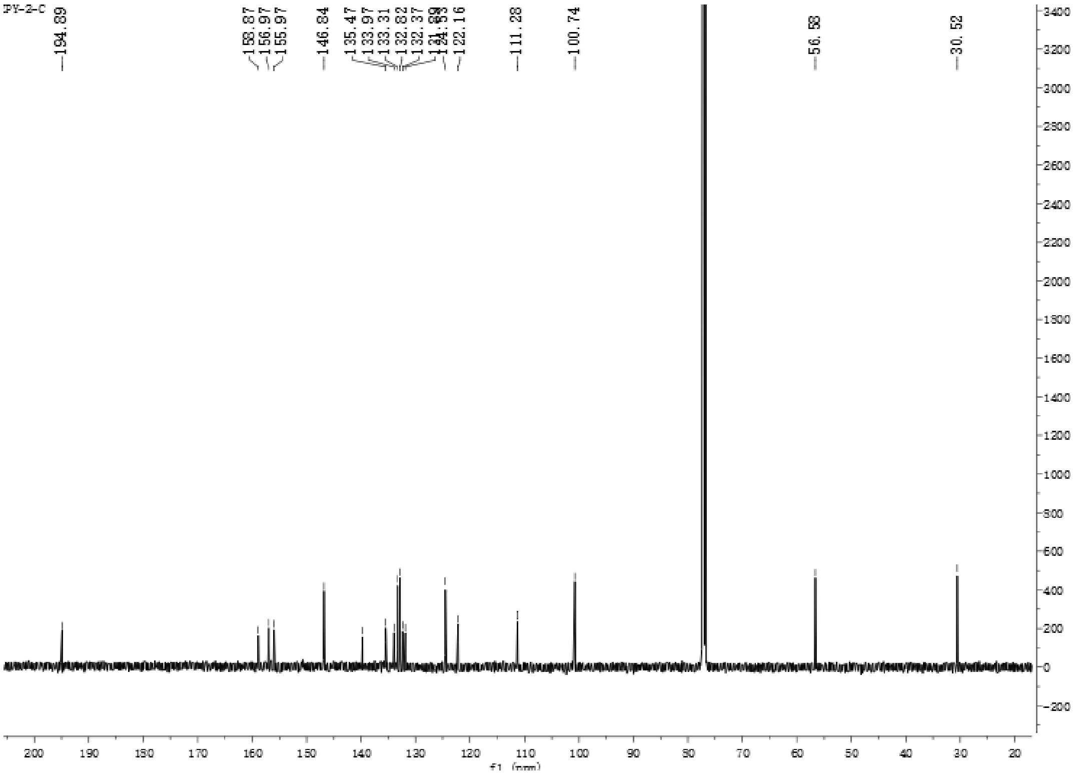

Figure S65 ^{13}C NMR of 10h

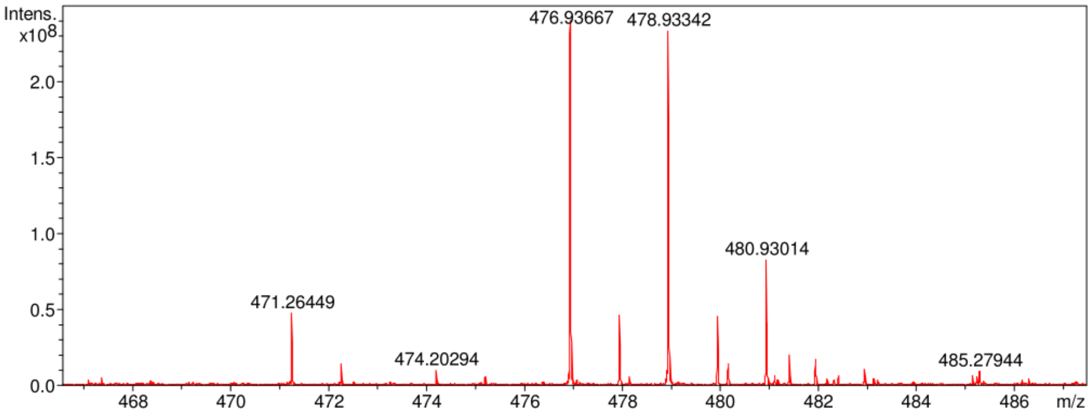

Figure S66 HRMS of 10h

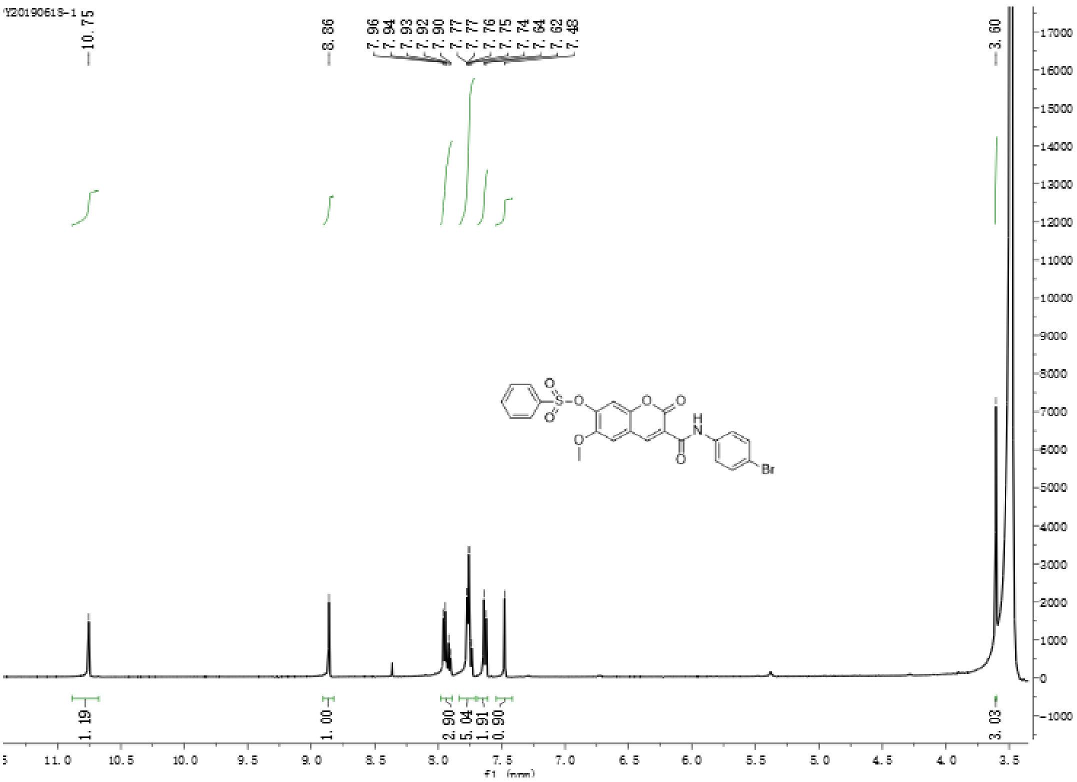

Figure S67 ^{1}H NMR of11a

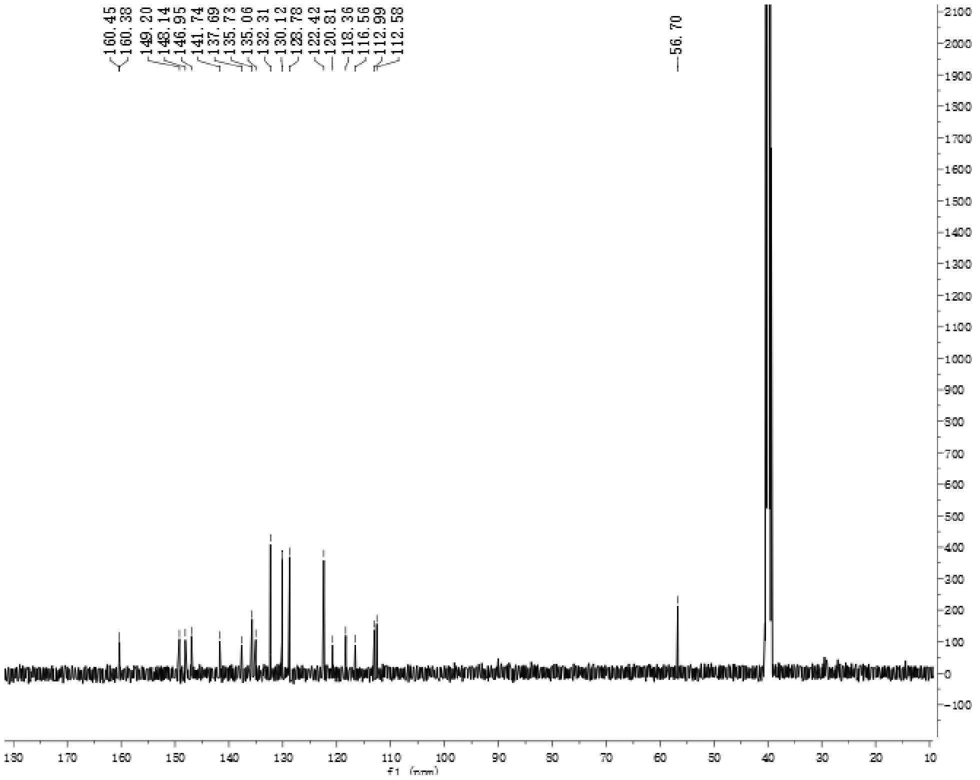

Figure S68 ^{13}C NMR of 11a

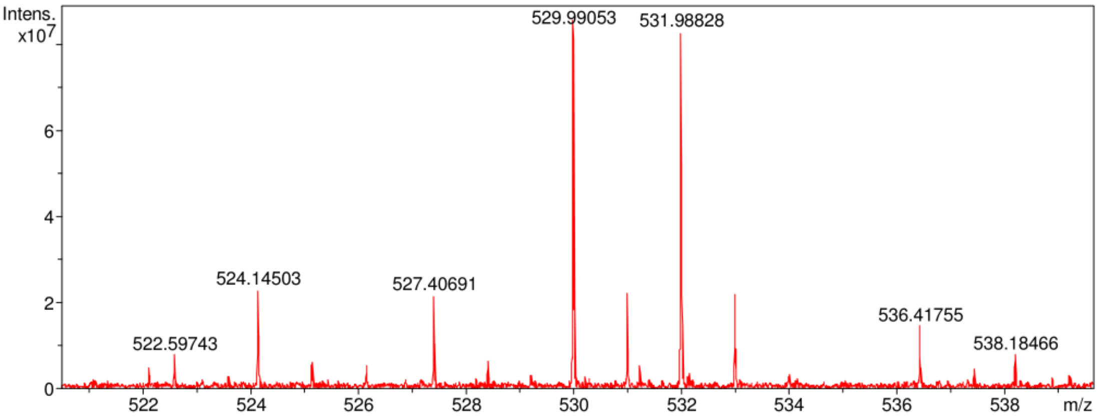

Figure S69　HRMS of 11a

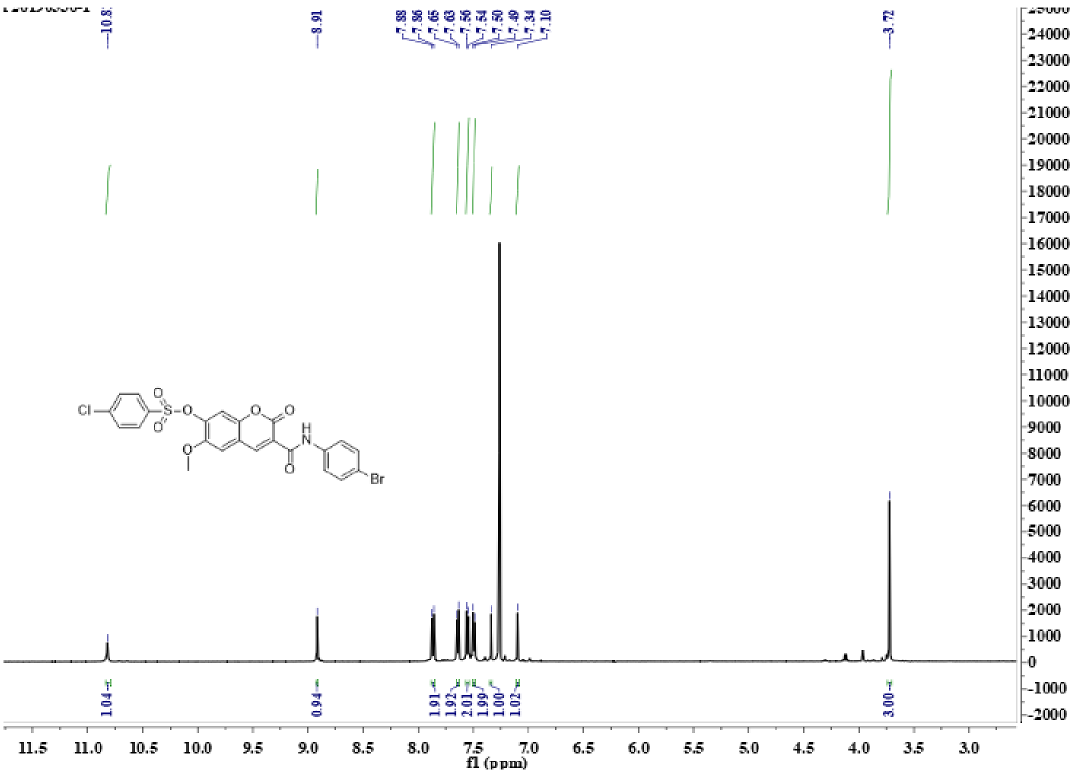

Figure S70　¹H NMR of 11d

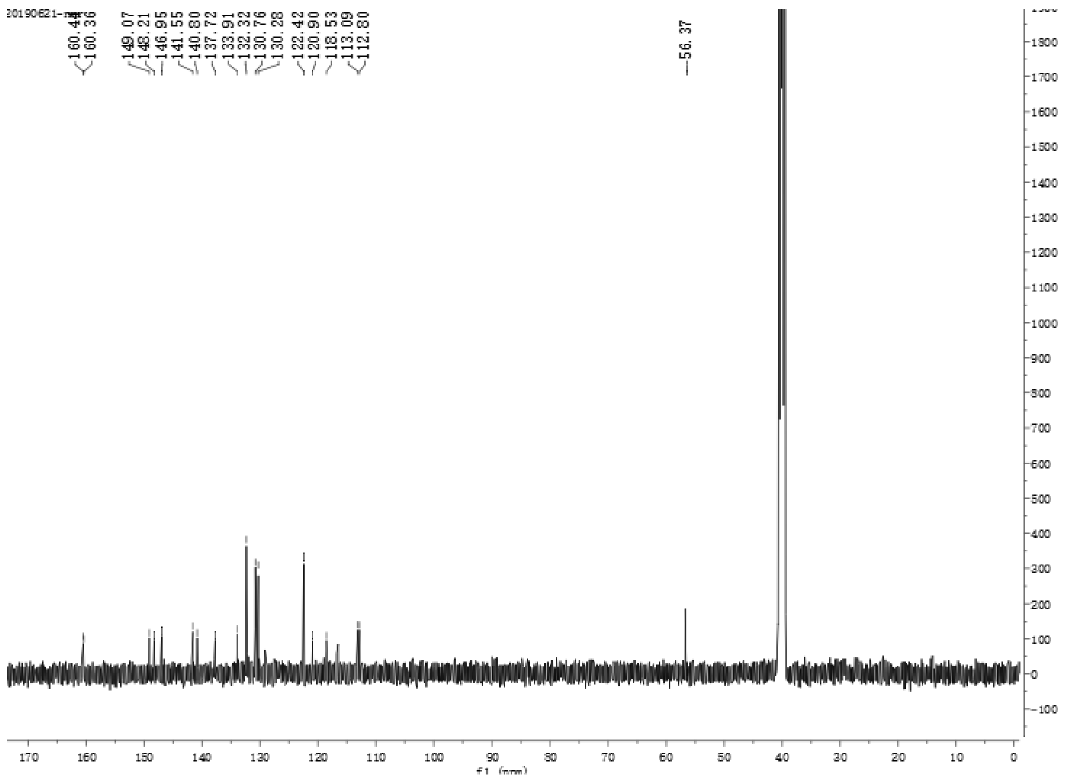

Figure S71 ^{13}C NMR of 11d

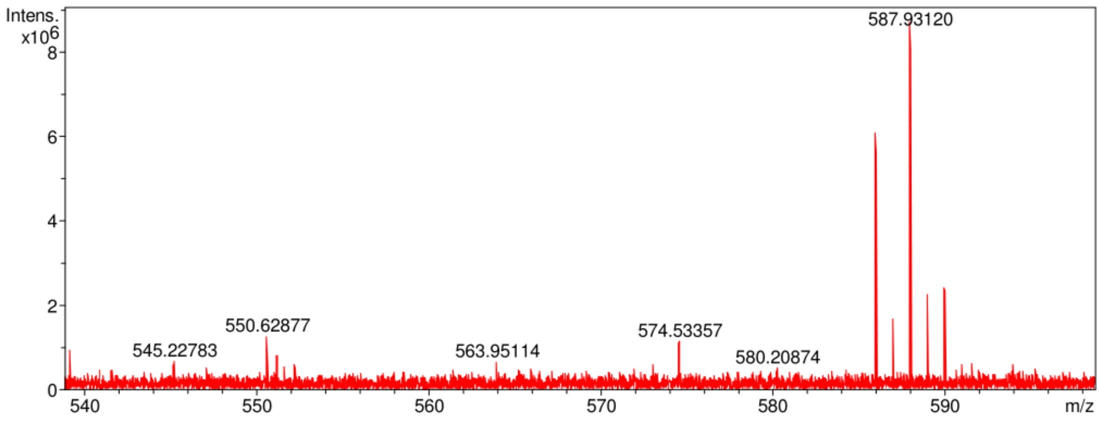

Figure S72 HRMS of 11d

Table S1. Acaricidal activities (LC_{50}, mg/mL, 72 h), AChE inhibitions (IC_{50}, μg/mL) of title compounds and their physicochemical properties

Compd.	LC_{50} (95% CI)	Slope±SE	X^2	IC_{50} values	MW [a]	clog *P* [a]	TPSA [a]
**4**	0.144 (0.0523-0.445)	0.23±0.080	0.213	105.98±21.87	192.2	1.35	55.76
**4a**	0.448 (0.223-0.861)	0.62±0.21	0.625	143.31±9.14	333.0	2.47	78.90
**4b**	0.209 (0.0325-0.377)	0.12±0.060	1.916	110.22±11.32	363.1	2.70	88.13
**4c**	0.318 (0.192-0.519)	0.75±0.16	0.855	126.91±29.39	351.0	2.62	78.90
**4d**	0.529 (0.298-0.966)	0.75±0.22	2.466	81.57±12.35	367.0	3.19	78.9
**4e**	0.636 (0.354-2.188)	0.67±0.16	2.645	141.15±14.41	413.0	3.34	78.90
**4f**	0.441 (0.215-0.930)	0.53±0.14	1.644	168.01±28.07	401.0	3.36	78.90
**4g**	0.183 (0.114-0.325)	0.49±0.090	0.955	133.10±8.44	378.0	2.22	130.71
**4h**	0.832 (0.348-2.025)	0.60±0.33	3.122	183.58±11.26	436.9	4.20	78.90
**4i**	0.489 (0.261-0.873)	0.71±0.25	0.659	146.02±17.18	339.0	2.15	78.90
**4j**	0.0930 (0.0566-0.178)	0.42±0.13	0.213	56.75±2.10	383.1	3.65	78.90
**5**	0.116 (0.0645-0.236)	0.37±0.15	0.315	93.09±15.61	286.1	3.59	55.76
**5a**	0.0570 (0.0489-0.0923)	0.75±0.19	2.448	40.60±4.16	427.1	4.71	78.90
**5b**	0.338 (0.174-0.687)	0.50±0.13	0.525	153.43±15.44	457.1	4.94	88.13
**5c**	0.0940 (0.0756-0.147)	0.82±0.42	1.222	85.52±11.20	445.1	4.86	78.90
**5d**	0.0990 (0.0752-0.143)	0.47±0.26	0.316	68.86±8.32	461.0	5.43	78.90
**5e**	0.0800 (0.0551-0.112)	0.81±0.38	0.415	71.12±14.10	509.0	5.58	78.90
**5f**	0.104 (0.0654-0.162)	0.57±0.17	0.266	119.64±15.70	495.1	5.60	78.90
**5g**	0.0680 (0.0447-0.119)	0.59±0.12	0.919	123.18±24.77	472.0	4.46	130.71
**5h**	0.0960 (0.0645-0.145)	0.66±0.22	0.626	104.83±15.43	530.9	6.43	78.90
**5i**	0.0730 (0.0526-0.124)	0.59±0.24	0.318	110.46±7.82	433.0	4.39	78.90
**5j**	0.0200 (0.0145-0.0421)	0.61±0.16	0.615	54.83±6.39	477.1	5.89	78.90
**6**	0.0810 (0.0563-0.131)	0.58±0.28	0.429	92.59±16.84	243.2	0.84	72.83
**6d**	0.159 (0.0933-0.262)	0.54±0.14	0.327	145.00±11.20	409.0	3.65	95.97
**6h**	0.107 (0.0752-0.161)	0.67±0.33	0.518	131.15±17.52	478.9	2.65	95.97
**7**	0.0820 (0.0544-0.126)	0.68±0.14	1.216	76.88±1.59	390.2	2.82	84.86
**7a**	0.117 (0.0852-0.181)	0.63±0.16	0.589	117.40±7.08	532.0	4.22	108.00
**7d**	0.0890 (0.0645-0.133)	0.68±0.22	0.978	98.17±7.61	587.9	4.93	108.00
**Cyetpyrafen**	0.0150 (0.0126-0.0384)	0.49±0.19	0.325	14.06±10.20	393.2	5.15	65.69

[a] These values were extracted from ChemBioDraw Ultra12.0; MW: molecular weight; clog *P*: partition coefficient; TPSA: topological polar surface area.

Table S2. Lethal activities of title compounds to Artemia（LC_{50}, mg/mL）

Compd.	6 h			12 h		
	LC_{50} (95% CI)	Slope±SE	X^2	LC_{50} (95% CI)	Slope±SE	X^2
**4**	0.0272 （0.0143-0.0481）	0.83±0.19	0.992	0.0142 （0.00825-0.0261）	1.29±0.15	2.923
**4a**	0.0475 （0.0284-0.0773）	1.14±0.26	2.195	0.0291 （0.0175-0.0486）	1.04±0.22	1.832
**4b**	0.0386 （0.0231-0.0660）	1.08±0.14	0.771	0.0182 （0.0112-0.0328）	1.29±0.26	2.215
**4c**	0.0915 （0.0591-0.141）	1.95±0.22	2.555	0.0563 （0.0381-0.0823）	0.28±0.020	1.342
**4d**	0.0336 (0.0221-0.0495)	1.30±0.16	1.958	0.0160 （0.00925-0.0281）	1.36±0.050	2.391
**4e**	0.157 (0.0714-0.351)	1.62±0.33	2.955	0.0898 （0.0521-0.153）	1.59±0.10	8.615
**4f**	0.109 (0.0682-0.173)	2.01±0.36	2.061	0.0756 （0.0481-0.118）	1.45±0.11	0.416
**4g**	0.0467 (0.0321-0.0664)	1.72±0.12	4.324	0.0256 （0.0184-0.0336）	2.26±0.24	1.747
**4h**	0.131 (0.0712-0.243)	1.87±0.23	1.577	0.125 （0.0616-0.254）	1.43±0.030	2.148
**4i**	0.0765 (0.0451-0.129)	1.32±0.14	0.956	0.0451 （0.0315-0.0654）	1.39±0.23	3.214
**4j**	0.0136 (0.00515-0.0345)	0.95±0.03 0	0.235	0.00936 (0.00323-0.0227)	1.37±0.15	1.763
**5**	0.0525 (0.0326-0.0841)	1.02±0.11	1.468	0.0325 (0.0224-0.0436)	1.34±0.13	3.411
**5a**	0.0958 (0.0564-0.159)	1.86±0.12	1.722	0.0558 (0.0399-0.0765)	1.98±0.25	5.715
**5b**	0.0945 (0.0561-0.157)	1.55±0.13	1.471	0.0362 (0.0283-0.0475)	1.65±0.27	7.428
**5c**	0.0947 (0.0514-0.176)	1.45±0.22	2.895	0.0486 (0.0295-0.0781)	1.10±0.17	1.645
**5d**	0.0728 (0.0373-0.139)	0.98±0.15	1.589	0.0337 (0.0233-0.0473)	1.33±0.090	2.314
**5e**	0.114 (0.0687-0.179)	1.92±0.22	0.960	0.0795 (0.0526-0.121)	1.65±0.12	0.636
**5f**	0.0715 (0.0431-0.118)	1.34±0.13	1.533	0.0399 (0.0237-0.0685)	0.89±0.16	0.0690
**5g**	0.0866 (0.0572-0.129)	1.96±0.16	1.217	0.0536 (0.0399-0.0715)	1.87±0.33	1.157
**5h**	0.106 (0.0581-0.195)	1.66±0.18	0.134	0.0576 (0.0401-0.0823)	1.77±0.22	3.085
**5i**	0.0998 (0.0611-0.161)	1.94±0.19	2.018	0.0567 （0.0393-0.0794）	1.70±0.05	5.344
**5j**	0.0779 (0.0534-0.114)	1.99±0.36	2.455	0.0297 （0.0201-0.0423）	1.33±0.12	2.625
**6**	0.0565 (0.0327-0.0976)	1.02±0.12	1.223	0.0356 （0.0173-0.0527）	0.88±0.090	1.669
**6d**	0.0455 (0.0244-0.0823)	0.80±0.22	0.192	0.0279 (0.0142-0.0545)	0.88±0.16	1.388
**6h**	0.0485 (0.0301-0.0776)	1.04±0.36	1.394	0.0339 (0.0214-0.0526)	0.99±0.24	2.156
**7**	0.0523 (0.0331-0.0773)	1.24±0.06 0	2.198	0.0323 (0.0201-0.0463)	1.10±0.040	1.271
**7a**	0.0576 (0.0245-0.139)	0.65±0.11	1.587	0.0336 (0.0188-0.0519)	0.95±0.15	0.652
**7d**	0.0542 (0.0381-0.0762)	1.66±0.32	2.074	0.0394 (0.0327-0.0466)	2.98±0.36	5.325
**Cyetpyrafen**	0.0117 (0.00935-0.0141)	2.05±0.39	3.791	0.00613 (0.00508-0.00727)	1.24±0.20	2.156

Table S3. The molecular docking scores of compound **4j** and the acetylcholinesterase

Name	Pose	Grid Score (kcal/mol)	Grid_vdw[a] (kcal/mol)	Grid_es[b] (kcal/mol)	Internal energy repulsive (kcal/mol)	Cluster Size
**4j**	1	-64.654701	-60.91954	-3.735158	4.798172	4
**4j**	2	-64.482681	-62.427189	-2.055494	6.316545	2
**4j**	3	-64.171875	-64.39386	0.221982	6.168889	3

[a] vdw: van der Waals interaction. [b] es: electrostatic interaction.

Table S4. The molecular docking scores of compound **5a** and the acetylcholinesterase

Name	Pose	Grid Score (kcal/mol)	Grid_vdw[a] (kcal/mol)	Grid_es[b] (kcal/mol)	Internal energy repulsive (kcal/mol)	Cluster Size
**5a**	1	-65.409058	-64.687553	-0.721504	5.695736	8
**5a**	2	-63.488708	-57.522991	-5.965718	11.793267	1

[a] vdw: van der Waals interaction. [b] es: electrostatic interaction.

Table S5. The molecular docking scores of compound **5j** and the acetylcholinesterase

Name	Pose	Grid Score (kcal/mol)	Grid_vdw[a] (kcal/mol)	Grid_es[b] (kcal/mol)	Internal energy repulsive (kcal/mol)	Cluster Size
**5j**	1	-72.95015	-72.541328	-0.40882	8.8318	5
**5j**	2	-69.114731	-68.981514	-0.133219	8.012092	3
**5j**	3	-67.184402	-64.211487	-2.972916	11.853454	1

[a] vdw: van der Waals interaction. [b] es: electrostatic interaction.
